# TREM2 improves neurological dysfunction and attenuates neuroinflammation, TLR signaling and neuronal apoptosis in the acute phase of intracerebral hemorrhage

**DOI:** 10.3389/fnagi.2022.967825

**Published:** 2022-10-24

**Authors:** Sidan Liu, Xuezhao Cao, Zhe Wu, Shumin Deng, Hefei Fu, Yanzhe Wang, Fang Liu

**Affiliations:** ^1^Department of Anesthesiology, the First Hospital of China Medical University, Shenyang, China; ^2^Department of Neurology, the First Hospital of China Medical University, Shenyang, China

**Keywords:** intracerebral hemorrhage, TREM2, TLR4, neuroinflammation, microglia, TAK242

## Abstract

Neuroinflammation contributes to secondary brain injury following intracerebral hemorrhage (ICH). Triggering receptor expressed on myeloid cells 2 (TREM2) confers strong neuroprotective effect by suppressing neuroinflammatory response in experimental ischemic stroke. This study aimed to clarify the neuroprotective role of TREM2 and potential underlying mechanism in a mouse model of ICH and *in vitro*. Adeno-associated virus (AAV) and green fluorescent protein-lentivirus (GFP-LV) strategies were employed to enhance TREM2 expression in the C57/BL6 mice and BV2 cells, respectively. The adult male C57/BL6 mice were subjected to ICH by administration of collagenase-IV in 1 month after the AAV particles injection. An *in vitro* ICH model was performed with oxygen hemoglobin in BV2 cells. Toll-like receptor 4 (TLR4) antagonist TAK242 was applied at 6 h following ICH. Neurological function, TREM2, pro-inflammatory cytokines, brain water content and Terminal deoxynucleotidyl transferase dUTP nick end labeling (TUNEL) staining were evaluated at 24 h following ICH. TLR4, NF-κB and mitogen-activated protein kinases (MAPK) signaling pathways were also determined by Western blot analysis at the same time point. The levels of TREM2 were increased at 12 h, peaked at 24 h and recovered on 7d following ICH. TREM2 overexpression ameliorated ICH induced neurological dysfunction, inhibited neuroinflammation, and attenuated apoptosis and brain edema. Further mechanistic study revealed that TREM2 overexpression inhibited TLR4 activation and NF-κB and MAPK signaling pathways. ICH increased the percentage of TUNEL-positive cells, which was markedly decreased by TREM2 overexpression. A similar improvement was also observed by the administration of TAK242 following ICH. TREM2 improves neurological dysfunction and attenuates neuroinflammation and neuronal apoptosis in the acute phase of ICH, which is, at least in part, mediated by negatively regulating TLR4 signaling pathway. These findings highlight TREM2 as a potential target for early brain injury following ICH.

## Introduction

Intracerebral hemorrhage (ICH) is a common and severe cerebrovascular disease, resulting in neurological deficits, high mortality and disability rates (Li et al., 2018). Increasing evidence indicates that ICH-induced secondary brain injury plays an important role in the deterioration of neurological function (Atangana et al., 2017; Sugiyama et al., 2018). Several mechanisms are involved in the pathogenesis of secondary brain injury following ICH, including neuroinflammation, oxidative stress, blood–brain barrier (BBB) disruption, and neuronal apoptosis (Chen et al., 2016; Bao et al., 2020; Gautam et al., 2020; Xue and Yong, 2020). The ICH-induced neuroinflammation plays a crucial role in the progression of secondary brain injury. Therefore, inhibiting neuroinflammation may be a novel therapeutic target for ICH.

Microglia, the resident innate immune cells in the central nervous system, are demonstrated to exacerbate neuroinflammatory response in the acute phase of ICH (Wang et al., 2018). Emerging evidence indicates that microglia-mediated pro-inflammatory cytokines, including interleukin (IL)-1β, tumor necrosis factor (TNF)-α, chemokines and other toxic chemicals, involve in ICH-induced brain injury, resulting in brain edema, BBB disruption, and neuronal apoptosis (Zhou et al., 2014, Wu et al., 2017a, Li et al., 2018). ICH-induced neuronal apoptosis leads to neutrophils and leukocytes infiltration into the brain, which further exacerbates neuroinflammatory injury (Kono and Rock, 2008; Wang et al., 2018). Therefore, inhibiting microglial activation and the subsequent neuroinflammation is an important therapeutic strategy for ICH-induced neuronal injury.

TREM2 is a pivotal endogenous transmembrane receptor, which is selectively and highly expressed on microglia. TREM2 regulates critical functions of microglia, including promoting cell survival, facilitating phagocytosis, and suppressing pro-inflammatory cytokines production (Ulland et al., 2017). TREM2 deficiency enhanced IL-1β, IL-6, and TNF-α expression in microglia, while TREM2 overexpression decreased the levels of pro-inflammatory cytokines (Jiang et al., 2014; Zheng et al., 2016). Accumulating evidence indicated that TREM2 alleviated neuroinflammation in experimental ischemic stroke (Kawabori et al., 2015; Zhai et al., 2017; Wu et al., 2017b). Recent research indicates the potential neuroprotective role of TREM2 following ICH (Chen et al., 2020). However, its function and mechanism require further study.

Toll-like receptor 4 (TLR4) is critical to modulate microglial activation and neuroinflammation. A study by Akamatsu demonstrated that subarachnoid hemorrhage induced TLR4 activation, which further activated nuclear factor-κB (NF-κB) and mitogen-activated protein kinases (MAPK) signaling pathways (Akamatsu et al., 2020). The NF-κB pathway resulted in microglial activation and the subsequent neuroinflammatory response (Wan et al., 2016, Shi et al., 2019). The MAPK signaling pathway serves as a downstream target of TLR4, which is likely to play a vital role in the process of ICH. Recent data indicated that TREM2 negatively regulated TLR4-mediated neuroinflammation (Ito and Hamerman, 2012). Therefore, we speculated that TREM2 inhibited ICH-induced neuroinflammation by inhibiting TLR4 activation.

This study aimed to clarify the potential role of TREM2 in the ICH mouse model and in the oxygen hemoglobin (OxyHb)- induced ICH cell culture model. Adeno-associated virus (AAV) and green fluorescent protein-lentivirus (GFP-LV)-mediated strategies were employed to enhance TREM2 expression in the present study. We hypothesized that TREM2 overexpression improved neurological dysfunction, inhibited neuroinflammation and attenuated neuronal apoptosis through inhibiting TLR4 signaling pathway in the acute phase of ICH.

## Materials and methods

### Animals

Male adult C57/BL6 mice (8 weeks old, weight 25-35 g) were housed in a temperature and humidity-controlled room with food and water *ad libitum* and exposed to a 12/12 h light/dark cycle. All animals were randomly divided into six groups: sham group, ICH group, ICH + AAV-NC group, ICH + AAV-TREM2 group, ICH + TAK242-NC group and ICH + TAK242 group (n = 6/group). An intracerebroventricular injection of AAV particles encoding the mouse TREM2 gene was performed in the ICH + AAV-TREM2 group and a control AAV vector was applied as a negative control (NC) in the mice of the ICH + AAV-NC group in 1 month before ICH induction. The ICH mouse model was generated under general anesthesia in all the ICH groups. TLR4 inhibitor TAK242 (3 mg/kg, ApexBio, USA, CAS number: 243984-11-4) was intraperitoneally injected in the ICH + TAK242 group at 6 h following ICH and dimethyl sulfoxide (DMSO) at the same volume and concentration was used as a negative control in the ICH + TAK242-NC group. All animal experiments were conducted in compliance with the Care and Use of Laboratory Animals of the National Institutes of Health and approved by the Institutional Animal Care and Use Committee of China Medical University.

### Experimental protocol

The mice received an intracerebroventricular injection of AAV particles encoding the mouse TREM2 gene. The efficacy of TREM2 upregulation by AAV transfection was verified in the sham group, LV-NC group, and LV-TREM2 group. The ICH mouse model was generated under general anesthesia in 1 month after the AAV particles injection. TAK242 was intraperitoneally injected at 6 h following ICH. The levels of TREM2 were measured at 0, 6 h, 12 h, 24 h, 3 days, 7 days, 14 days (*n* = 3/time point) following ICH *in vivo*. The forelimb placement test, the Bederson score and the corner turn test were applied to evaluate the neurological function at 24 h following ICH. The mice were sacrificed for neurochemical analyses following all behavior tests.

### ICH mouse models

The ICH mouse model was generated as previously described (Deng et al., 2020). Briefly, the mice were anesthetized with pentobarbital (40 mg/kg) and positioned prone in a stereotaxic head frame. A 1-mm cranial burr hole was drilled. Collagenase-IV (0.5 U, Solarbio Science & Technology, China) was injected into the right basal ganglia (0.2 mm posterior, 2.2 mm lateral to the right of bregma and 3 mm below the dura) *via* a 26-gauge needle of a 10-μL Hamilton syringe. A similar surgical procedure was performed and an equal volume of sterile saline instead of collagenase-IV was injected into the animals of the sham group. The rectal temperature was maintained at 37.0 ± 0.5°C throughout the surgery and recovery periods.

### Intracerebroventricular Adeno-associated viral infection

TREM2 gene was packaged into the GV626 Vector (Iba1p-EGFP-MCS-SV40 Poly A). A plasmid (pAAV-Iba1p-EGFP-3Flag-SV40 Poly A) not encoding TREM2 was used as a control AAV vehicle (AAV-NC; Genechem, China). The titers of AAV particles were between 1 × 10^12^ and 2 × 10^12^ vg/ml. The AAV-TREM2 and AAV-NC were delivered by bilateral stereotactic injections into the lateral ventricles using a micropipette attached to a 10 μl Hamilton syringe (1 μl/side) following the mouse brain atlas (2.5 mm posterior, 1 mm lateral to the right of bregma and 3 mm below the dura). After the injection, the needle was left *in situ* for 10 min, and then pulled out slowly for 3 min. The efficacy of TREM2 overexpression by AAV transfection was examined in AAV-NC group, and AAV-TREM2 group (*n* = 3/group).

### BV2 microglia culture and treatment

The BV2 microglia cell line was purchased from Cell Bank of the Institute of Biochemistry and Cell Biology (Shanghai, China, RRID: CVCL_0182) and cultured in MEM medium (HyClone, United States) with 10% FBS (Procell Life Science & Technology, China) and 1% penicillin/streptomycin (Solarbio Science & Technology, China). The BV2 cells were maintained at 37°C in a humidified atmosphere containing 95% air and 5% CO_2_.

A green fluorescent protein-TREM2-lentivirus (GFP-TREM2-LV) strategy was employed to enhance TREM2 expression in BV2 cells. As previously described, BV2 cells were seeded in 6-well plates, grown overnight and transfected with GFP-TREM2-LV to generate stable TREM2 overexpression cells (Li et al., 2022). A blank vector lentivirus was used as a negative control. The efficacy of TREM2 overexpression by GFP-TREM2-LV transfection was verified by fluorescence microscopy excitation at wavelengths ranging from 490 to 520 nm and western blot analysis in the normal group, LV-NC group, and LV-TREM2 group.

An ICH *in vitro* model was performed with 10 μM OxyHb (Solarbio Science & Technology, China). BV2 cells in the OxyHb+TAK242 group were pretreated with TAK242 (100 nM) for 1 h before OxyHb induction. The levels of TREM2 were tested at 3, 6, and 12 h following the administration of OxyHb. TREM2, TLR4 and apoptosis were determined by western blot analysis and immunofluorescence in the normal group, OxyHb group, OxyHb+LV-TREM2 group, and OxyHb+TAK242 group (*n* = 3/group).

### Neurological function evaluation

Neurological function was evaluated by the forelimb placement test, the Bederson score and the corner turn test by an assessor who was blind to the experimental groups. For the corner turn test, the mice were allowed to advance into a 30° angle corner and exit by turning either to the left or right. Choice of turning was recorded for a total of 10 trials, and a score was calculated as the number of left turns/all trials × 100%. The Bederson score was graded on a 5-point scale as follows: grade 0 = no observable neurological deficit; grade 1 = unable to extend the contralateral forelimb; grade 2 = flexion of the contralateral forelimb; grade 3 = mild circling to the contralateral side; grade 4 = severe circling; and grade 5 = falling to the contralateral side. For the forelimb placement test, the placement of ipsilateral forelimb on the countertop was recorded when the vibrissa was stimulated. The percent of the ipsilateral forelimb placement out of 10 total trials was calculated.

### Brain water content measurement

Brain edema was evaluated by measuring brain water content using the standard wet-dry method as previously described (Zhao et al., 2017). Briefly, the mice were sacrificed at 24 h following ICH, and the brain without the cerebellum and brain stem was harvested. The wet weight of ipsilateral and contralateral hemispheres was acquired, respectively. Then, the brain samples were dehydrated at 100°C for 24 h to obtain the dry weight. Brain water content was determined according to the following formula: (wet weight-dry weight)/wet weight× 100%.

### Western blot analysis

Brain and BV2 cell protein samples were homogenized and lysed with RIPA lysis buffer (Beyotime, China). Protein concentration was determined by BCA assay (Biosharp, China). Equal amounts of protein (30 μg/lane) were separated on SDS-PAGE and transferred to nitrocellulose membranes. The membrane was blocked with 5% bovine serum albumin (BSA) at room temperature for 1 h and incubated overnight at 4°C with the following primary antibodies: TREM2 (ab86491, Abcam, RRID: AB_1925525), TLR4 (66,350, Proteintech, RRID: AB_2881730), p-ERK (31,779, Cell Signaling Technology, RRID: AB_2095853), total ERK (4,695, Cell Signaling Technology, RRID: AB_390779), p-p38 MAPK (4,511, Cell Signaling Technology, RRID: AB_2139682), p38 MAPK (8,690, Cell Signaling Technology, RRID: AB_10999090), p-NF-κB p-p65 (3,033, Cell Signaling Technology, RRID: AB_331284), NF-κB p65 (8,242, Cell Signaling Technology, RRID: AB_331284), TNF-α (PY19810, Abmart, RRID: AB_2920645), IL-1β (MB0684, Abmart, RRID: AB_2920646), iNOS (18985-1-AP, Proteintech, RRID: AB_2782960), CD206 (TD4149, Abmart, RRID: AB_2920647), β-tubulin (M20005, Abmart, RRID: AB_2920648). Appropriate secondary antibody Goat Anti-Rabbit & Mouse IgG-HRP (M21003, Abmart, RRID: AB_2920649) was incubated with the membrane for 1 h at room temperature. Specific bands were detected using enhanced chemiluminescence (ECL, Bioshrap, China) and analyzed using ImageJ version 1.8.0 software (National Institutes of Health, Bethesda, MD, United States).

### Immunofluorescence

As previously described, the cells were fixed with 4% paraformaldehyde for 15 min, and incubated with the primary antibodies including TLR4 (T62186, Abmart, RRID: AB_2920650) and TREM2 (ab86491, Abcam, RRID: AB_1925525) antibodies. Then, the cells were washed three times with PBS and incubated with the corresponding secondary antibodies for 1 h at room temperature. Finally, the nuclei were counterstained with 4′,6-diamidino-2-phenylindole (DAPI). All images were captured with a fluorescence microscope (Nikon, Japan) and analyzed by using ImageJ software.

### TUNEL staining

The brains were harvested under general anesthesia with isoflurane and postfixed with 4% paraformaldehyde (PFA) for 24 h. Double-immunofluorescence staining of DAPI (blue) and TUNEL (red) was performed to detect apoptosis according to the manufacturer’s instructions at 24 h after ICH. The number of TUNEL-positive neurons was counted at × 200 magnification using ImageJ software. The data were expressed as the ratio of TUNEL-positive cells (%).

### Statistical analysis

Statistical analysis was performed with Graph Pad Prism 9.0 software. One-way analysis of variance (ANOVA) followed by Tukey’s *post hoc* test was used for multiple group comparisons. Comparisons between two groups were analyzed by unpaired *t*-test. The brain edema content and the expression levels of TREM2 and TLR4 in immunofluorescence were analyzed using a repeated two-way ANOVA. ImageJ was applied to quantify fluorescence image or gray density. The data were expressed as the mean ± SEM with *p* < 0.05 considered statistically significant.

## Results

### ICH enhanced TREM2 expression in a time-dependent manner

As indicated in Supplementary Figure 1, the protein levels of TREM2 were significantly upregulated at 12 h, reached a peak at 24 h, and recovered on 7d following ICH when compared with the sham group. Similarly, a high level of TREM2 was observed in BV2 cells at 12 h after OxyHb induction. These data indicated that ICH significantly enhanced microglial TREM2 expression in a time-dependent manner.

The AAV particles and lentivirus containing TREM2 cDNA increased protein levels of TREM2 in the C57/BL6 mice and BV2 cells, respectively, The efficacy of TREM2 upregulation by AAV transfection was confirmed by western blot analysis in the C57/BL6 mice. Comparted to that in the AAV-NC group, the protein level of TREM2 was significantly increased in 1 month after injection with AAV particles (Supplementary Figure 2A). Similarly, the upregulation of TREM2 was also observed in the BV2 cells following GFP-TREM2-LV transfection (Supplementary Figure 2B).

### Upregulation of TREM2 ameliorated ICH-induced neurological deficits

**Figure 1:**
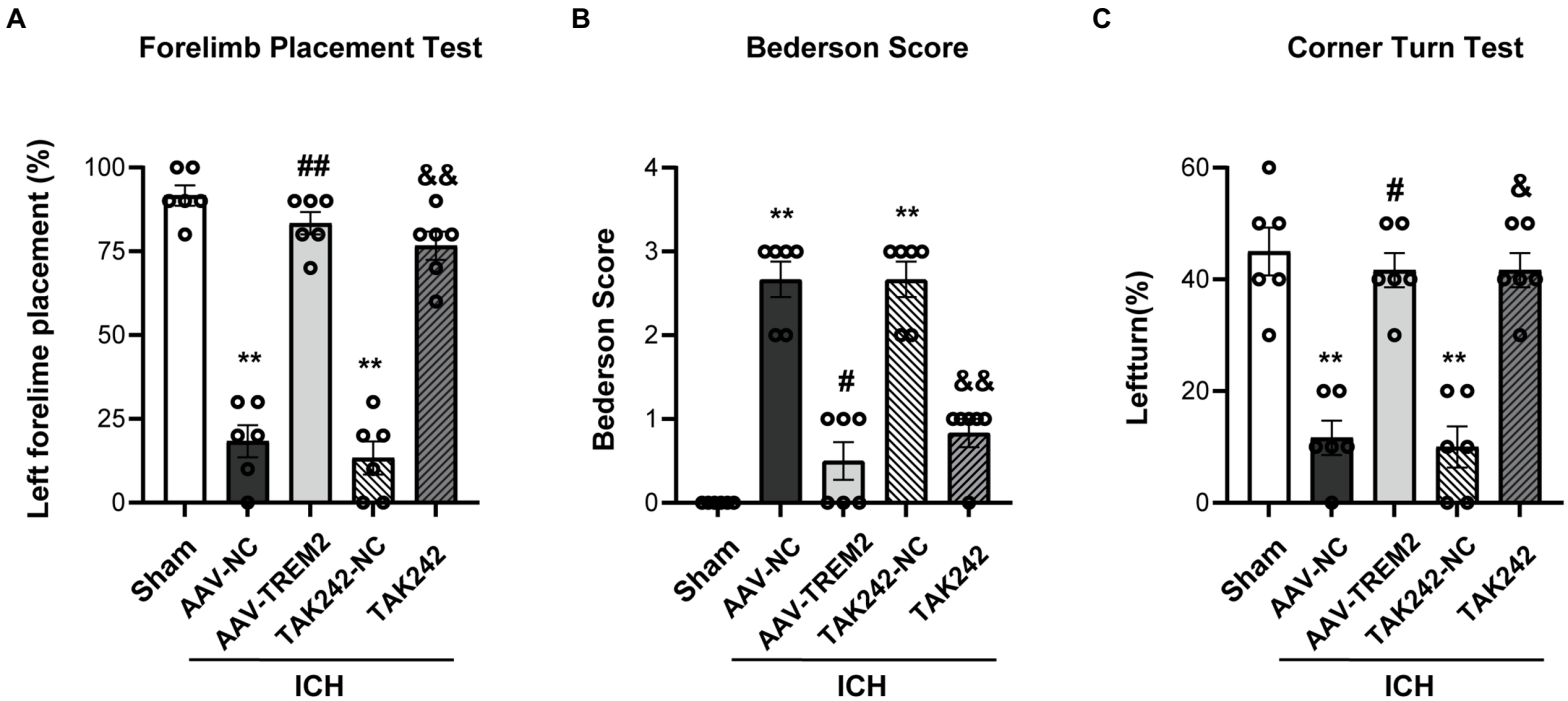
TREM2 overexpression and TLR4 antagonist TAK242 alleviated intracerebral hemorrhage-induced neurological dysfunction. **(A)** Forelimb placement test. **(B)** Bederson score. **(C)** Corner turn test. ^**^p < 0.01, vs. the sham group, ^#^*p* < 0.05, ^##^*p* < 0.01, vs. the AAV-NC + ICH group, ^&^*p* < 0.05, ^&&^*p* < 0.01, vs. the TAK242-NC + ICH group. ICH: intracerebral hemorrhage, AAV, Adeno-associated virus, LV, lentivirusl NC, negative controls. The data were represented as mean ± SEM. *n* = 6 per group.

The forelimb placement test, the Bederson score and the corner turn test were used to investigate the effect of TREM2 on ICH-induced neurological deficits. The significant neurological impairments were observed in the ICH group when compared with the sham group at 24 h following ICH. TREM2 overexpression significantly ameliorated neurological impairment in the acute phase of ICH (Figure 1). The similar improvement was also observed for neurological scores in the TAK242 + ICH group (Figure 1).

### TREM2 overexpression alleviated neuroinflammation following ICH both *in vivo* and *in vitro*

**Figure 2:**
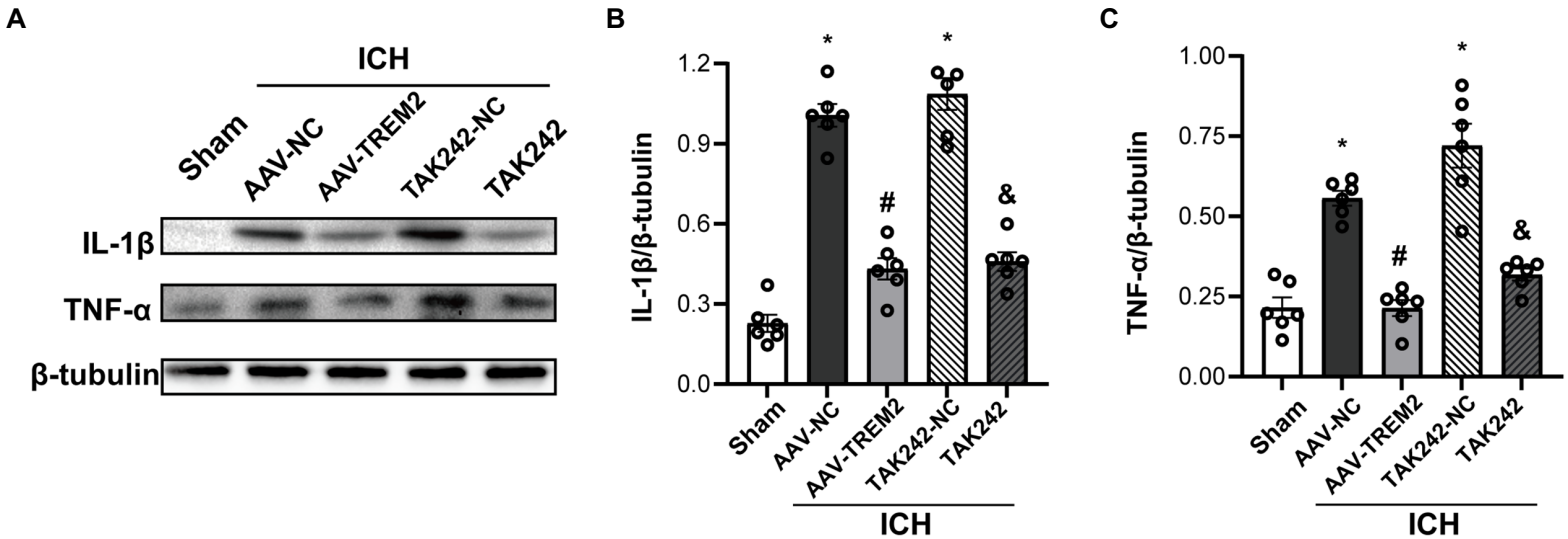
TREM2 overexpression alleviated intracerebral hemorrhage-induced neuroinflammation in the C57/BL6 mice. **(A–C)** The protein levels of IL-1β and TNF-α in the C57/BL6 mice. ^*^p < 0.05, vs. the sham group, ^#^*p* < 0.05, vs. the AAV-NC + ICH group, ^&^*p* < 0.05, vs. the TAK242-NC + ICH group. ICH: intracerebral hemorrhage, AAV, Adeno-associated virus, LV, lentivirus; NC, negative controls. The data were represented as mean ± SEM. *n* = 6 per group.

Neuroinflammation plays a crucial role in the progression of ICH-induced brain injury. As shown in Figure 2, compared with the sham group, the protein levels of IL-1β and TNF-α were significantly higher at 24 h following ICH in the C57/BL6 mice. TREM2 overexpression markedly inhibited IL-1β and TNF-α production. Similarly, ICH significantly enhanced IL-1β and TNF-α expression in BV2 cells and TREM2 overexpression decreased the levels of IL-1β and TNF-α following OxyHb administration.

### TREM2 inhibited TLR4/MAPK signaling pathway

**Figure 3:**
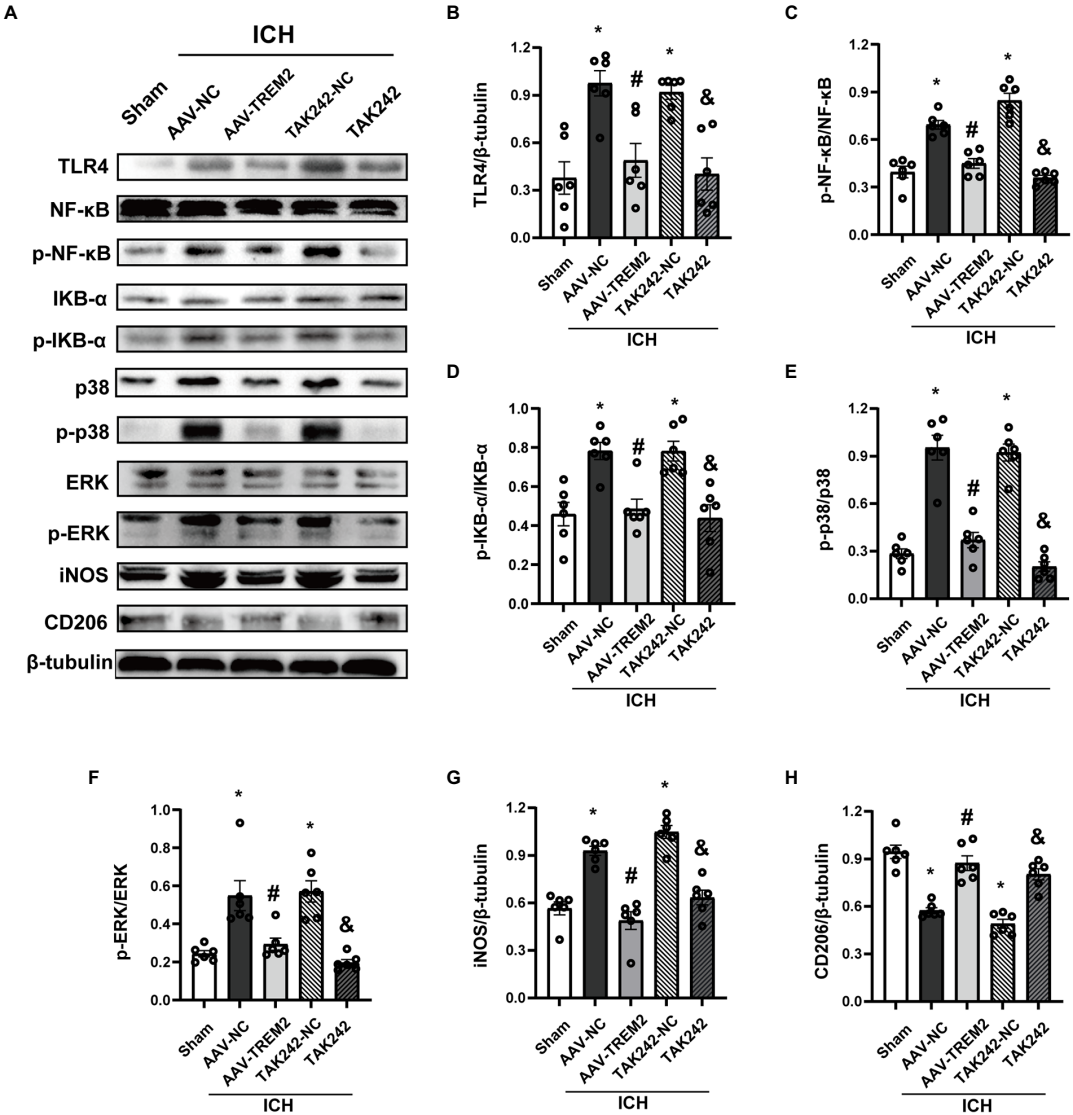
TREM2 overexpression inhibited TLR4-NF-κB and TLR4-MAPK pathways. **(A)** Representative western blot bands. **(B–H)** Quantitative analyses of TLR4, phosphorylated NF-κB, IKB-α, p38 and ERK, iNOS and CD206 in the ipsilateral hemisphere at 24 h after intracerebral hemorrhage. ^*^p < 0.05, vs. the sham group, ^#^*p* < 0.05, vs. the AAV-NC + ICH group, ^&^*p* < 0.05, vs. the TAK242-NC + ICH group. ICH: intracerebral hemorrhage, AAV, Adeno-associated virus; LV, lentivirus; NC, negative controls. The data were represented as mean ± SEM. *n* = 6 per group.

TLR4 pathway contributes to the progression of neuroinflammation following ICH. To characterize the underlying mechanism of the role of TREM2 in this ICH model, the effect of TREM2 on TLR4-mediated neuroinflammatory response was investigated. TLR4 protein was significantly upregulated at 24 h following ICH, which was inhibited by TREM2 overexpression (Figures 3A,B). Similarly, TAK242 reduced TLR4 expression following ICH (Figures 3A,B).

NF-κB inflammatory signaling pathway is the downstream of TLR4 and has been shown to be negatively regulated by TREM2. As shown in Figures 3A,C,D, ICH significantly enhanced the phosphorylation of NF-κB and IKB-α. TREM2 overexpression attenuated the activation of NF-κB. The similar result was observed following TAK242 administration.

MAPK signaling, which has also shown to be one of the downstream of TLR4, activates multiple transcription factors. ICH significantly induced phosphorylation of p38 and ERK, which was significantly inhibited by TREM2 overexpression (Figures 3A,E,F). TAK242 administration also reduced the phosphorylation of p38 and ERK following ICH (Figures 3A,E,F).

iNOS was elevated at 24 h following ICH (Figures 3A,G). Conversely, TREM2 overexpression increased the levels of CD206 and inhibited iNOS expression (Figures 3A,G,H). Consistently, similar results were observed in the TAK242 + ICH group (Figures 3A,G,H). These data indicated that TREM2 and TAK242 suppressed ICH-induced neuroinflammation and enhanced anti-inflammatory cytokine expression.

### The interaction between TREM2 and TLR4 following ICH

**Figure 4:**
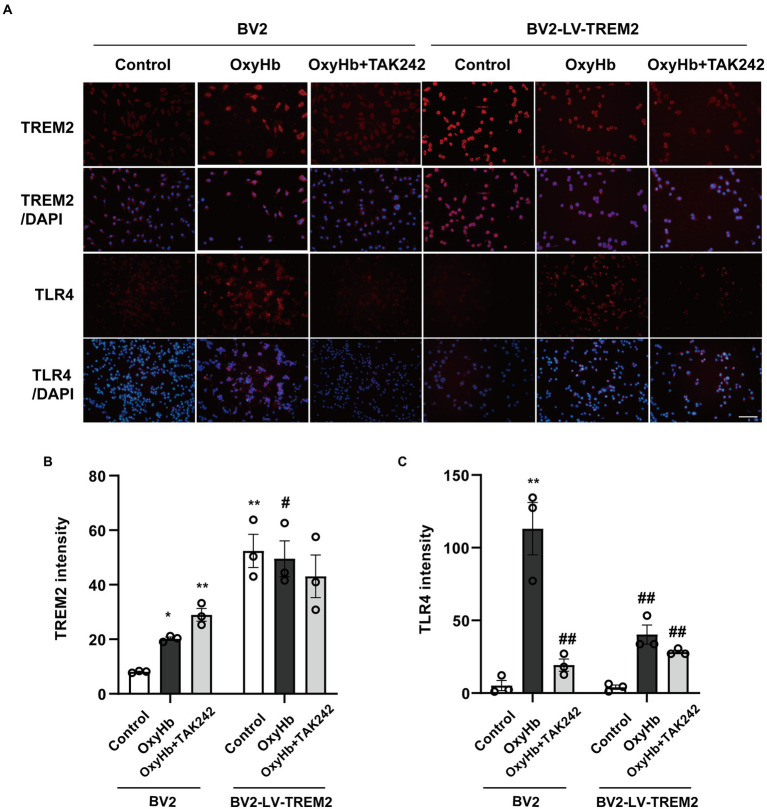
The interaction between TREM2 and TLR4 in the OxyHb-induced intracerebral hemorrhage cell culture model. **(A)** TREM2 and TLR4 immunochemical staining. **(B,C)** The levels of TREM2 and TLR4 in BV2 cells. *p < 0.05, **p < 0.01, vs. BV2 control group. ^#^*p* < 0.05, ^##^*p* < 0.01, vs. BV2 OxyHb group. LV, lentivirus; Scale bar: 100 μm. The data were represented as mean ± SEM. *n* = 3 per group.

The relationship between TREM2 and TLR4 was investigated in BV2 cells (Figure 4). TREM2 expression was upregulated and the level of TLR4 was increased in the OxyHb mimic ICH model. TREM2 overexpression increased the level of TREM2 and inhibited TLR4 expression following OxyHb administration. Interestingly, TLR4 antagonist TAK242 also enhanced TREM2 expression and reduced the level of TLR4 following ICH *in vitro*. Furthermore, the combination of TREM2 and TAK242 resulted in a further downregulation of TLR4. These data demonstrated that the interaction between TREM2 and TLR4 indicated the imbalance between neuroinflammation and anti-inflammation in the OxyHb-induced *in vitro* ICH model.

### TREM2 overexpression suppressed necroptosis following ICH

**Figure 5:**
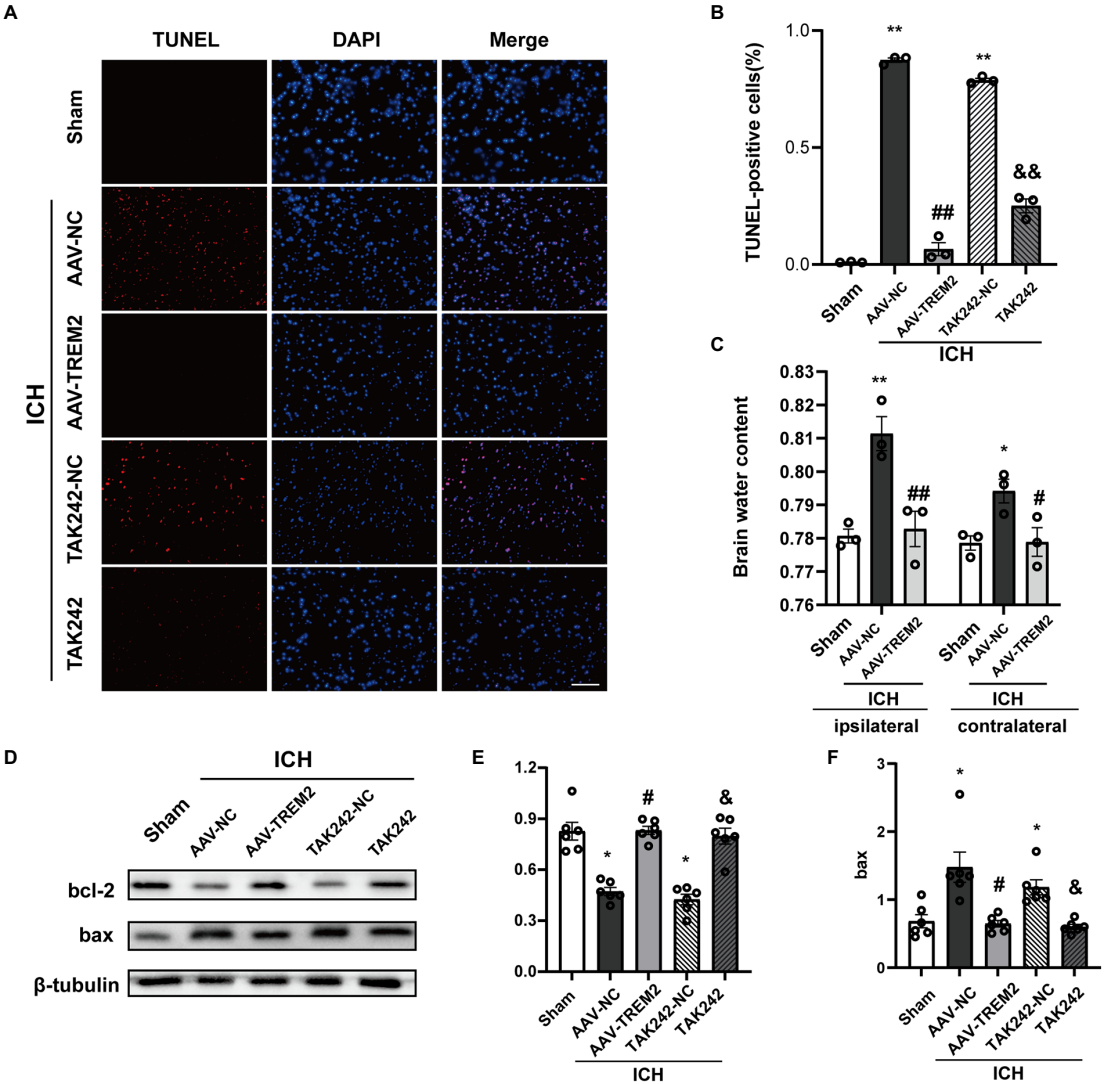
TUNEL staining and brain water content following intracerebral hemorrhage. **(A,B)** TUNEL staining of brain tissue (DAPI: blue, TUNEL: red), and quantitative analysis of TUNEL staining. ^**^p < 0.01, vs. the sham group, ^##^*p* < 0.01, vs. the AAV-NC + ICH group, ^&&^*p* < 0.01, vs. the TAK242-NC + ICH group. **(C)** Brain water content both in ipsilateral and contralateral hemisphere. ^**^p < 0.01, vs. corresponding sham group, ^#^*p* < 0.05, ^##^*p* < 0.01, vs. the corresponding AAV-NC + ICH group. **(D–F)** Expression levels of bcl2 and bax in different treated groups, ^*^p < 0.05, vs. the sham group, ^#^*p* < 0.05, vs. the AAV-NC + ICH group, ^&^*p* < 0.05, vs. the TAK242-NC + ICH group. ICH: intracerebral hemorrhage, AAV, Adeno-associated virus; LV, lentivirus; NC, negative controls. Scale bar: 100 μm. The data were represented as mean ± SEM. *n* = 3 per group.

The present results indicated that ICH increased the percentage of TUNEL-positive cells following ICH, which was markedly decreased by TREM2 overexpression or administration of TAK242 *in vivo* (Figures 5A,B). Similarly, the protein level of bcl-2 was increased and bax protein was upregulated following ICH, which was reversed by TREM2 overexpression (Figures 5D–F). The similar results were observed following TAK242 administration. Thus, the present data confirmed the neuroprotective effects of TREM2 following ICH.

### TREM2 overexpression attenuated brain edema following ICH

Brain edema volume was assessed by measuring brain water content. The brain water content in ipsilateral side was significantly increased following ICH (*p* < 0.05), and TREM2 overexpression attenuated this effect (*p* < 0.05; Figure 5C). The similar pattern was observed in contralateral side.

## Discussion

This study investigated the role of TREM2 in alleviating ICH-induced early brain injury. The present study found that TREM2 was upregulated in a time-dependent manner following ICH, indicating TREM2 involved in the progression of ICH. TREM2 overexpression alleviated ICH-induced neurological deficit and neuroinflammatory response in the ICH mouse model. Furthermore, TREM2 inhibited TLR4-mediated activation of NF-κB and MAPK signaling pathways, which involved in neuroprotective effects of TREM2 against ICH-induced neuroinflammation and neuron apoptosis.

Accumulating evidence indicated that TREM2 exerted neuroprotection by alleviating neuroinflammation in experimental cerebral ischemia (Kawabori et al., 2015, Wu et al., 2017b, Zhai et al., 2017). TREM2 deficiency attenuated microglial phagocytic activities and exacerbated apoptosis and neurological dysfunction, whereas TREM2 overexpression protected neurons against ischemic injury (Ulland et al., 2017, Wu et al., 2017b). This study demonstrated that TREM2 was increased in a time-dependent manner and reached a peak at 24 h following ICH. ICH-induced neurological deficits were evaluated using the forelimb placement test, the Bederson score and the corner turn test in this study. The results demonstrated that neurological impairments were observed at 24 h following ICH, and that TREM2 overexpression significantly ameliorated this effect. These data indicate that TREM2 protects against ICH-induced early brain injury.

Neuroinflammation is an essential pathological feature and is considered a major contributor to the progression of ICH. Increasing evidence has confirmed the ubiquitous role of TLR4 in the pathogenesis of ICH. TLR4 activated NF-κB and MAPK signaling pathways to enhance pro-inflammatory cytokines production, thus inducing the innate immune response and subsequent neurological deficits (Yenari et al., 2010; Zhao et al., 2014). TREM2 plays a critical role in modulating microglial-mediated neuroinflammatory response. TREM2 overexpression markedly ameliorated Aβ-induced neuroinflammation (Li et al., 2019). More importantly, TREM2 silencing enhanced LPS-induced neuroinflammatory response (Griciuc and Tanzi, 2021). The current study demonstrated that TREM2 overexpression inhibited TLR4 activation and subsequent pro-inflammatory cytokines production both *in vivo* and *in vitro*.

TREM2 is an efficient negative regulator of TLR4 signaling (Rosciszewski et al., 2018; Zhou et al., 2019; Vuono et al., 2020). TREM2 and TLR4 are both microglial surface receptors. TREM2 silencing enhanced neuroinflammatory response following TLR4 activation in macrophages or dendritic cells (Ito and Hamerman, 2012). Consistent with previous studies (Ren et al., 2018), TREM2 modulated neuroinflammation by downregulating TLR4-mediated activation of NF-κB signaling pathway in this study. Two major MAPKs ERK and p38 pathways were also involved in regulating pro-inflammatory cytokines expression during the pathological condition of ICH (Long et al., 2019). In this study, ERK and p38 were activated and phosphorylated following ICH and TREM2 overexpression inhibited p-ERK and p-p38 expression. The present study indicated that TREM2 modulated NF-κB and MAPK signaling pathways and inhibited TLR4 activation.

This study indicated that TLR4 protein expression was increased, and elevated levels of TREM2 were also observed in the brain of ICH-model mice, suggesting that anti-and pro-inflammatory effects coexisted in the initial stage of ICH. The initial bleeding induced the release of endogenous neuroinflammation-related ligands that activated both TLR4-dependent pro-inflammatory pathways and TREM2-dependent anti-inflammatory signaling (Painter et al., 2015; Akamatsu et al., 2020). Activated microglia play a beneficial or detrimental effect on the basis of gene expression profiles. In this study, TLR4 shifted microglial polarization to pro-inflammatory state following ICH. Conversely, TREM2 alleviated ICH-induced neuroinflammatory response through switching microglia toward anti-inflammatory phenotype. Furthermore, TREM2 overexpression inhibited TLR4 expression. This study demonstrated that the interaction between TREM2 and TLR4 played an important role in controlling the balance between pro-inflammatory and anti-inflammatory conditions.

The current study had some potential limitations. Firstly, this study only investigated the early neurological outcomes (within 72 h) following ICH, the effects of TREM2 on long-term neurological outcomes are required to investigate. Secondly, only the strategy of AAV vectors was employed to overexpress TREM2, future studies are required to determine whether silencing TREM2 can exacerbate ICH-induced neurological deficit. Thirdly, injecting bacterial collagenase itself can induce an inflammatory response. This study cannot rule out collagenase-induced neuroinflammatory response following ICH. Finally, this study only focused on MyD88-dependent signaling pathways downstream of TLR4. Thus, the current data could not fully exclude TRIF-dependent signaling that might be involved in TLR4 signaling pathway.

In summary, the present study provides novel evidence that TREM2 overexpression protects against neurological deficits and suppressed neuroinflammatory response, TLR4 signaling pathway, and neuronal apoptosis triggered by ICH. These findings provide insights into the potent neuroprotective effects of TREM2 in early brain injury following ICH. TREM2 overexpression may be a promising target in the management of ICH.

## Data availability statement

The raw data supporting the conclusions of this article will be made available by the authors, without undue reservation.

## Ethics statement

All animal experiments were conducted in compliance with the Care and Use of Laboratory Animals of the National Institutes of Health and approved by the Institutional Animal Care and Use Committee of China Medical University.

## Author contributions

XC and FL designed the experiment and drafted the manuscript. SL performed the experiments. SL, SD, HF, and ZW analyzed the data. ZW participated in the experimental design. All authors contributed to the article and approved the submitted version.

## Funding

This work was supported by National Natural Science Foundation of China (grant number 81300938); National Key Research and Development Program of China (SQ2018YFC200044 and 2020YFC2005300); and Liaoning Province Education Foundation (grant number QN2019007).

## Conflict of interest

The authors declare that the research was conducted in the absence of any commercial or financial relationships that could be construed as a potential conflict of interest.

## Publisher’s note

All claims expressed in this article are solely those of the authors and do not necessarily represent those of their affiliated organizations, or those of the publisher, the editors and the reviewers. Any product that may be evaluated in this article, or claim that may be made by its manufacturer, is not guaranteed or endorsed by the publisher.

## Abbreviations

AAV: Adeno-associated virus
GFP: Green fluorescent protein
GFP-LV: Green fluorescent protein-lentivirus
ICH: Intracerebral hemorrhage
LV: Lentivirus
MAPK: Mitogen-activated protein kinases
NF-κB: Nuclear factor-κB
OxyHb: Oxygen hemoglobin
TLR4: Toll-like receptor 4
TREM2: Triggering receptor expressed on myeloid cells 2
