# Supplementary figures and images for: TREM2 improves neurological dysfunction and attenuates neuroinflammation, TLR signaling and neuronal apoptosis in the acute phase of intracerebral hemorrhage
Source: Front Aging Neurosci. 2022 Oct 24;14:967825. doi: 10.3389/fnagi.2022.967825 (PMC9637852; doi:10.3389/fnagi.2022.967825)

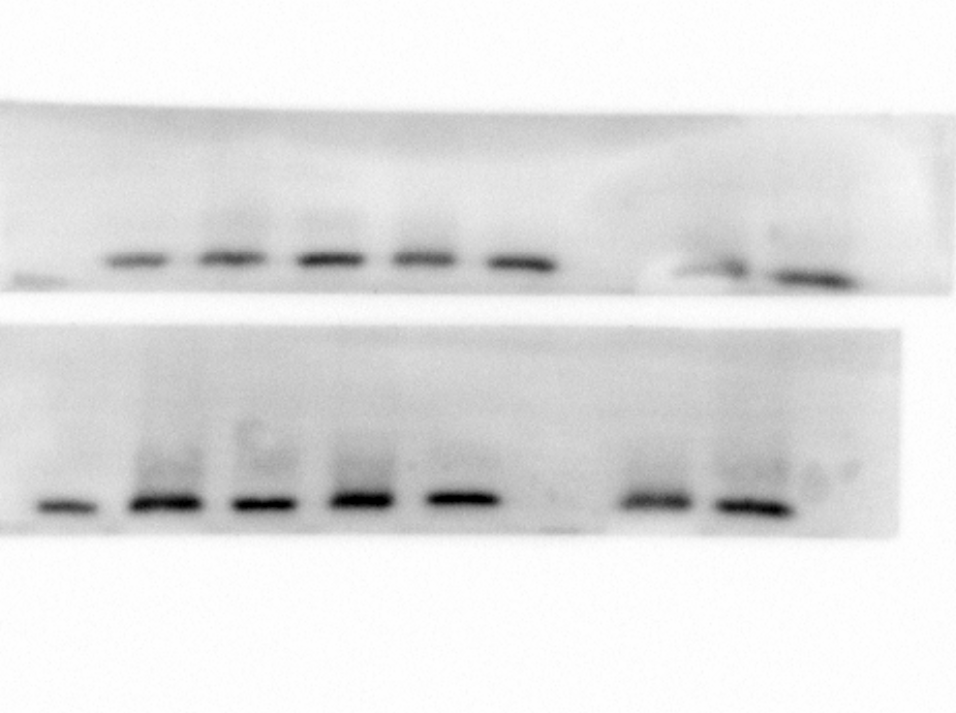

Supplement: Supplementary file 1 [file Data_Sheet_1.ZIP › Original Files of WB bands/bax1.tif]

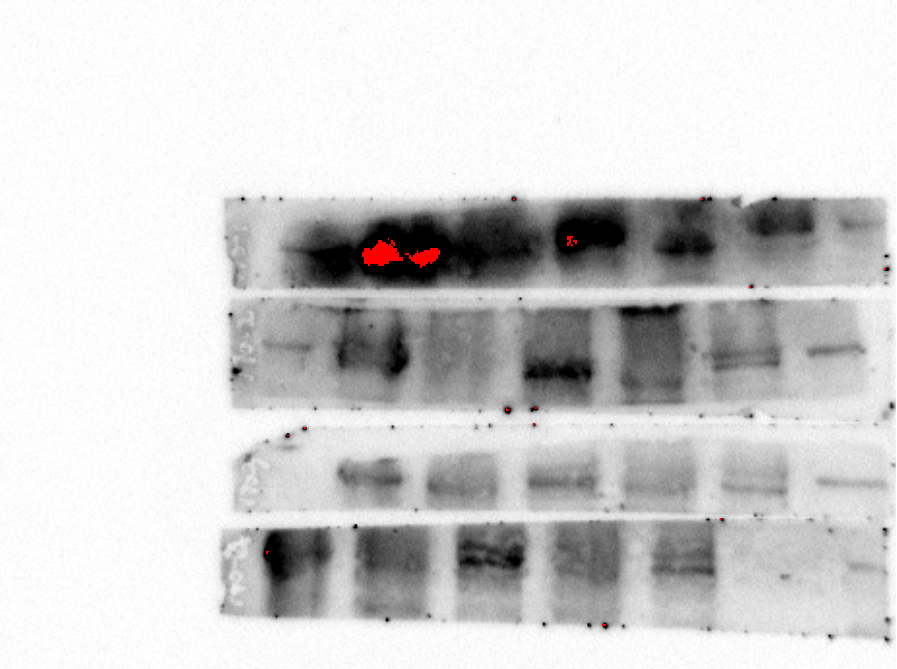

Supplement: Supplementary file 1 [file Data_Sheet_1.ZIP › Original Files of WB bands/CD206.tif]

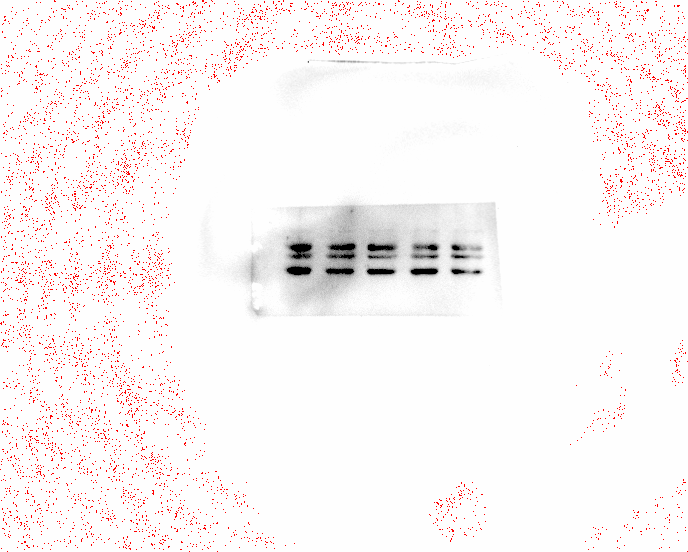

Supplement: Supplementary file 1 [file Data_Sheet_1.ZIP › Original Files of WB bands/erk.tif]

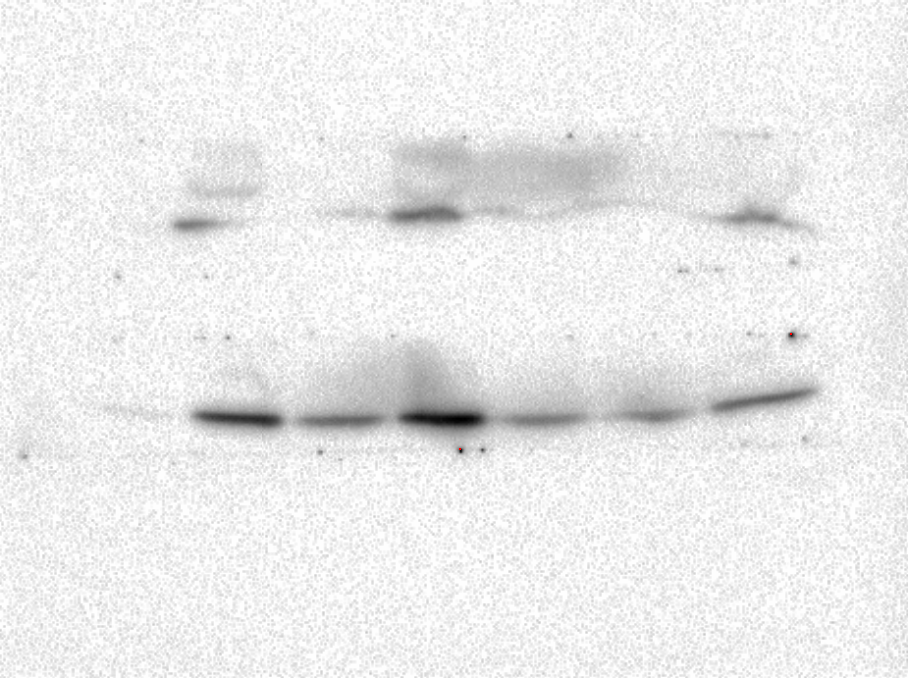

Supplement: Supplementary file 1 [file Data_Sheet_1.ZIP › Original Files of WB bands/IL-1β.tif]

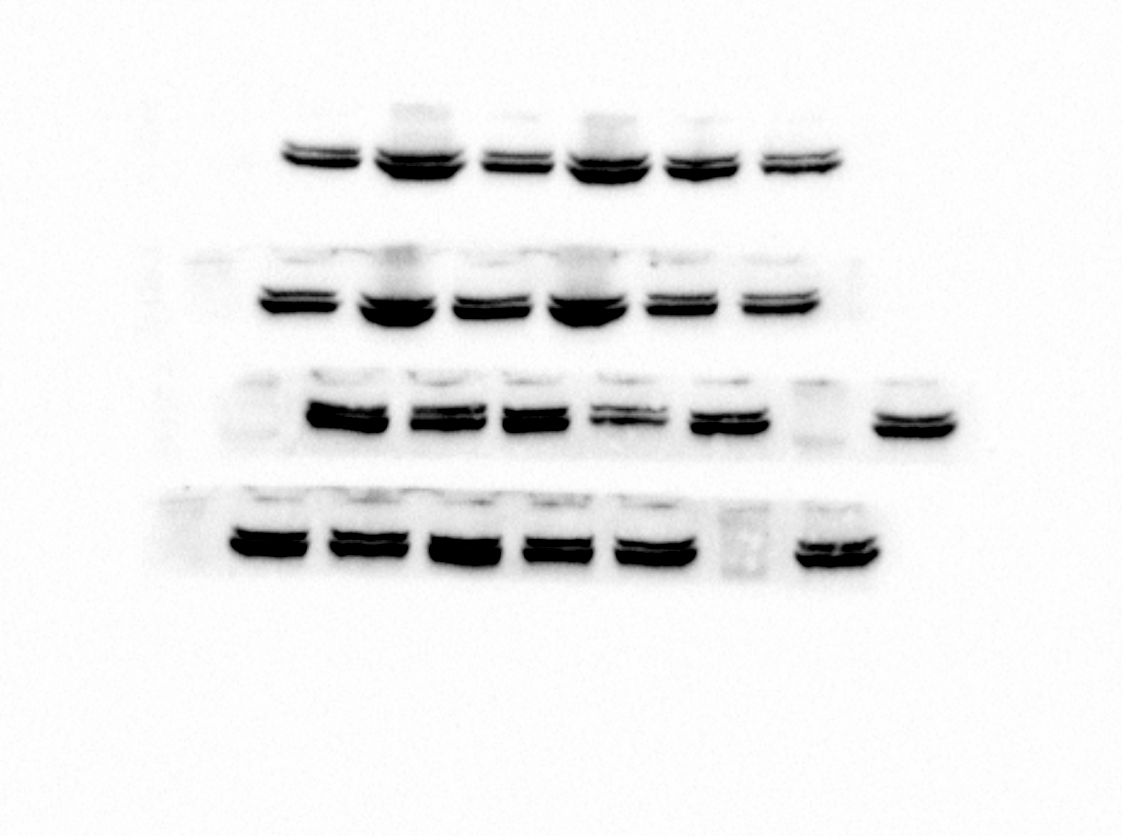

Supplement: Supplementary file 1 [file Data_Sheet_1.ZIP › Original Files of WB bands/inos.tif]

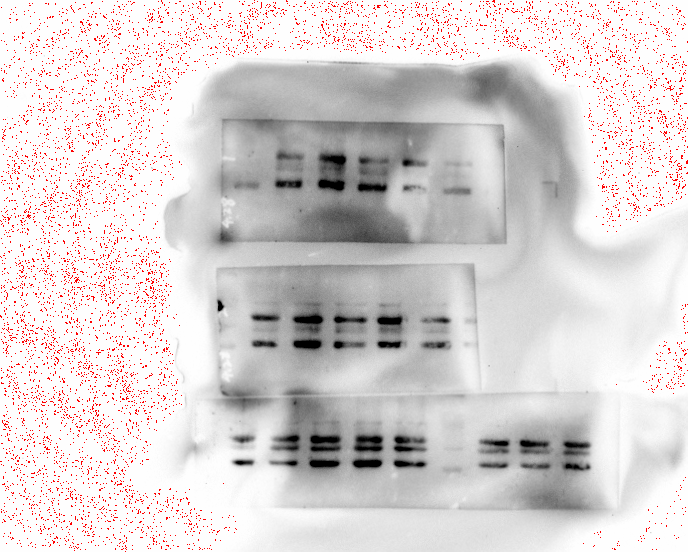

Supplement: Supplementary file 1 [file Data_Sheet_1.ZIP › Original Files of WB bands/p-erk.tif]

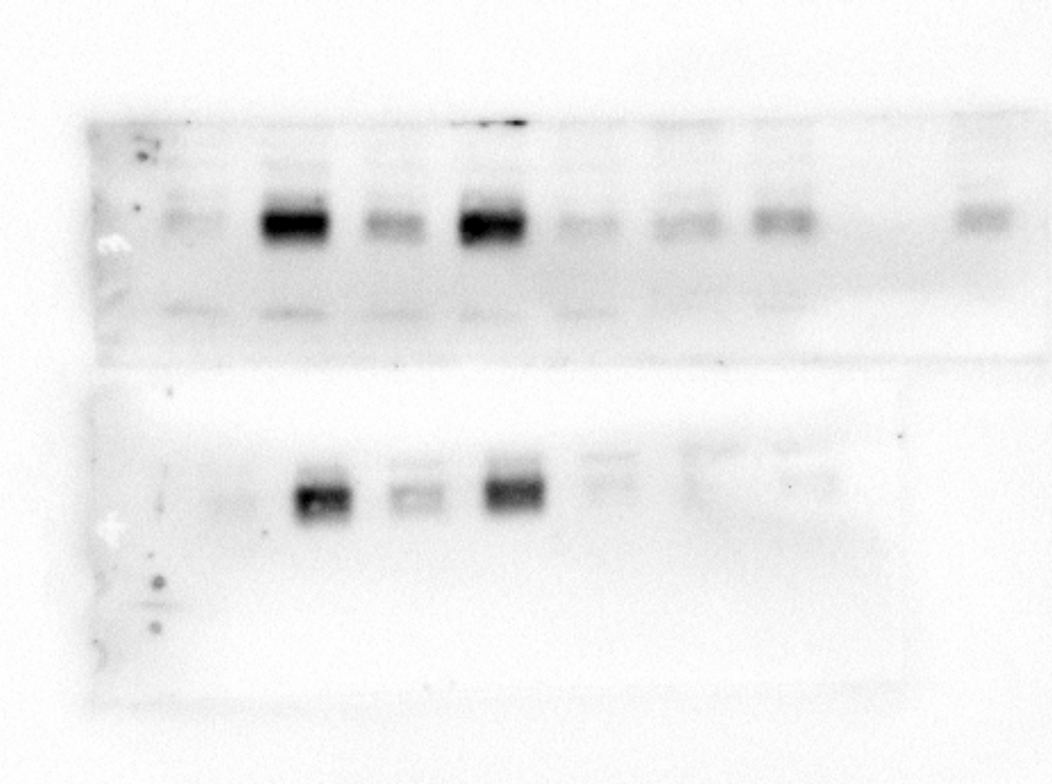

Supplement: Supplementary file 1 [file Data_Sheet_1.ZIP › Original Files of WB bands/p-p38.tif]

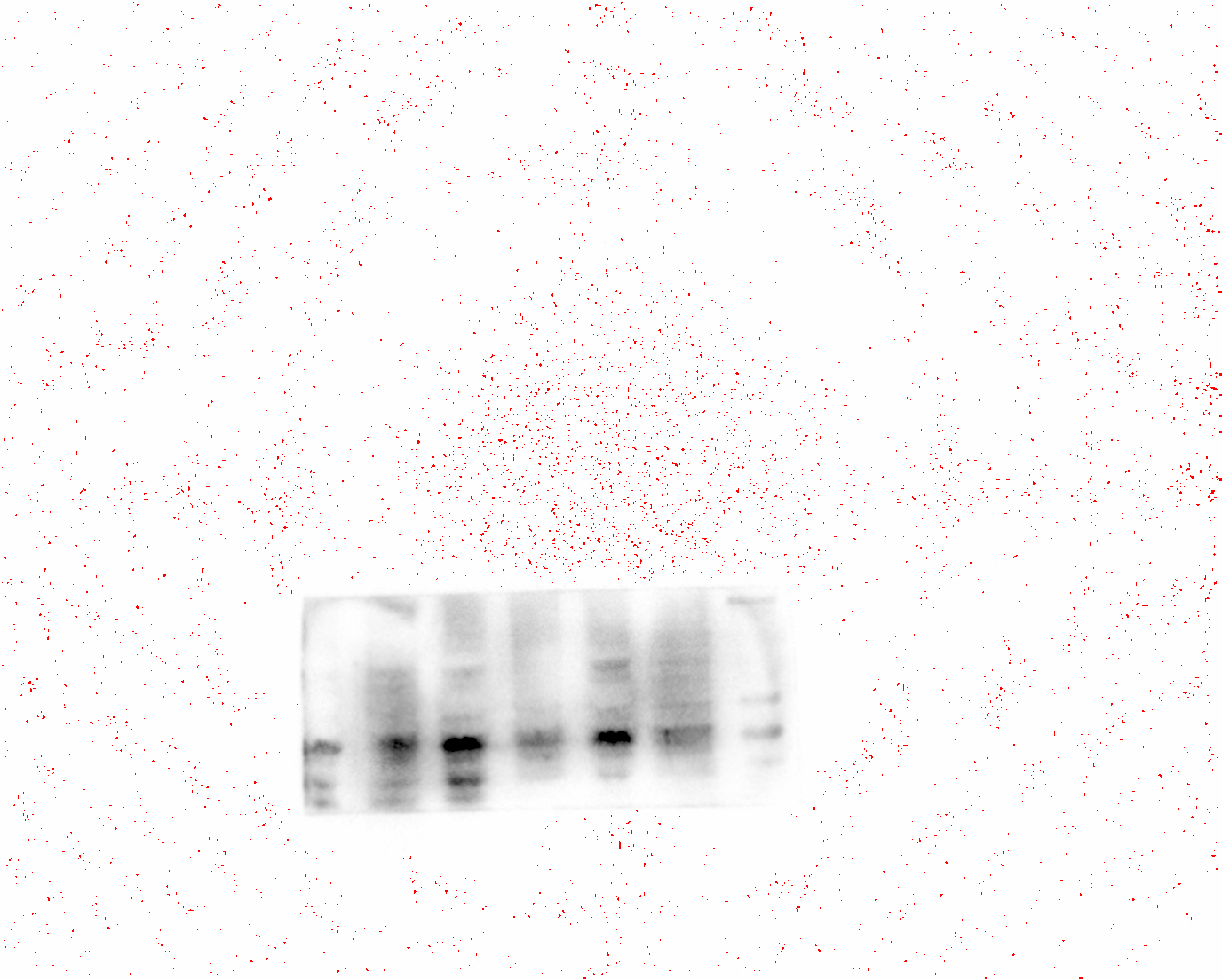

Supplement: Supplementary file 1 [file Data_Sheet_1.ZIP › Original Files of WB bands/p-p65.tif]

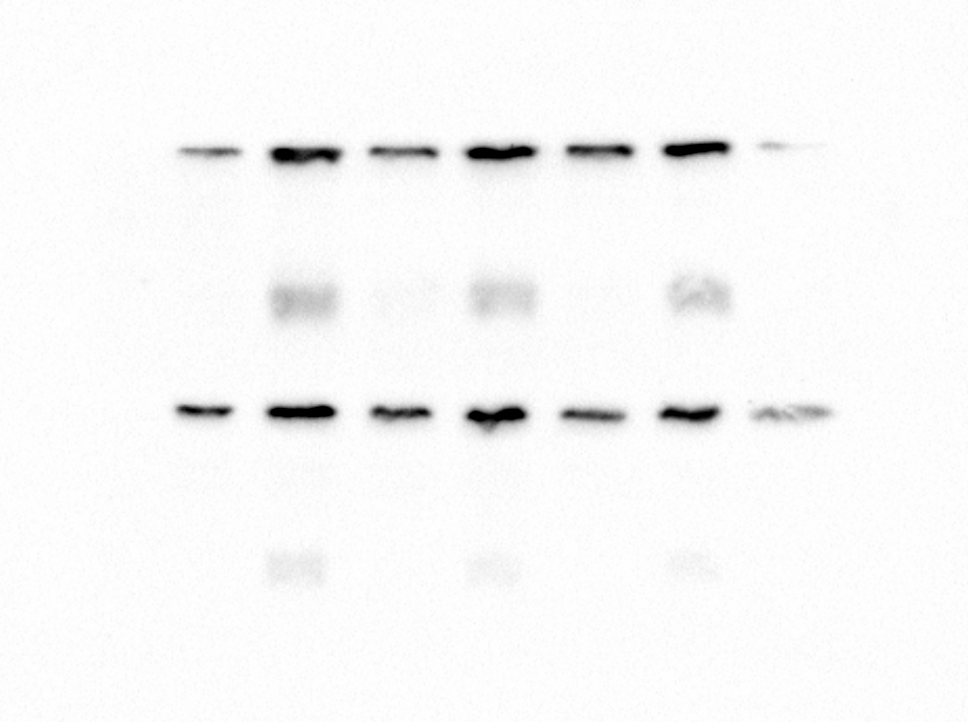

Supplement: Supplementary file 1 [file Data_Sheet_1.ZIP › Original Files of WB bands/p38.tif]

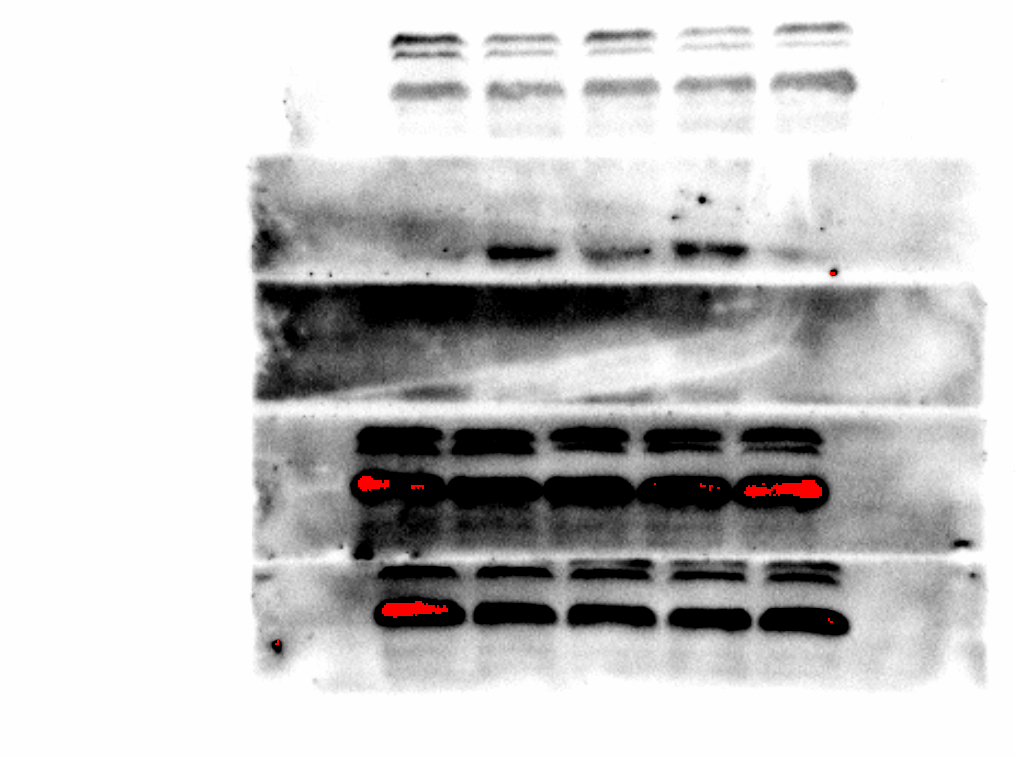

Supplement: Supplementary file 1 [file Data_Sheet_1.ZIP › Original Files of WB bands/P65 and its tubulin.tif]

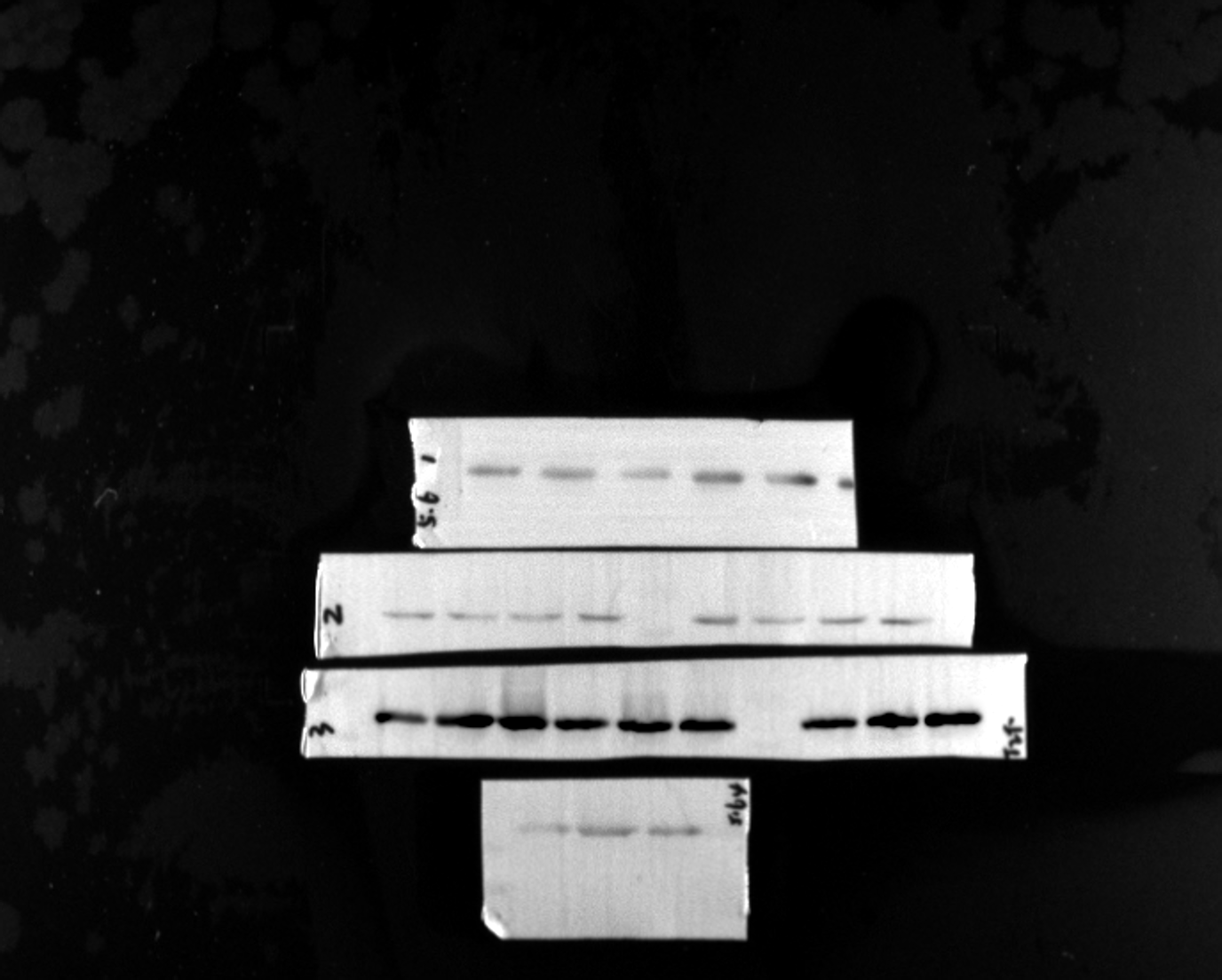

Supplement: Supplementary file 1 [file Data_Sheet_1.ZIP › Original Files of WB bands/the 2nd blot is the tubulin of TREM2 following Oxygen.Tif]

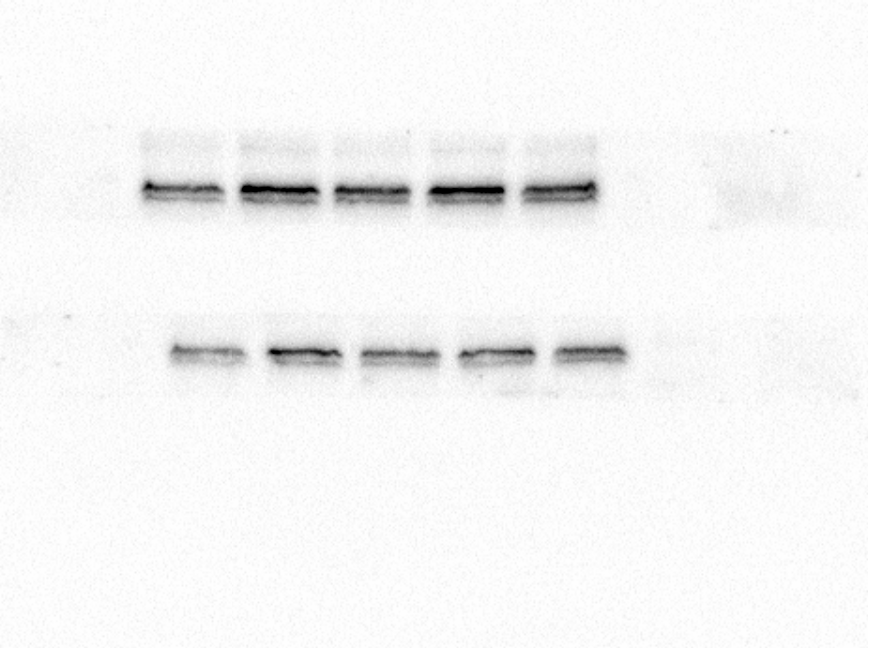

Supplement: Supplementary file 1 [file Data_Sheet_1.ZIP › Original Files of WB bands/TLR4.tif]

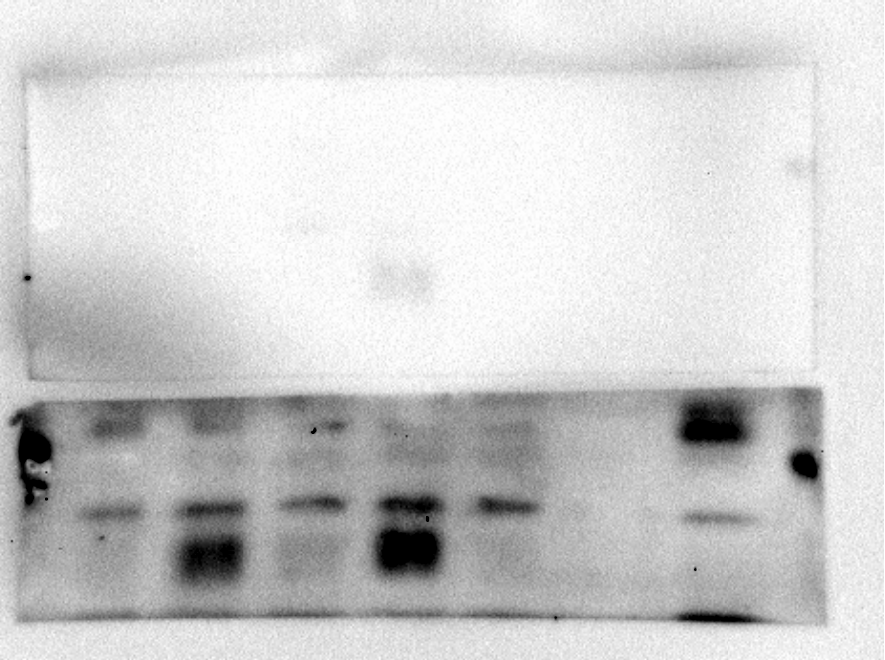

Supplement: Supplementary file 1 [file Data_Sheet_1.ZIP › Original Files of WB bands/TNFa.tif]

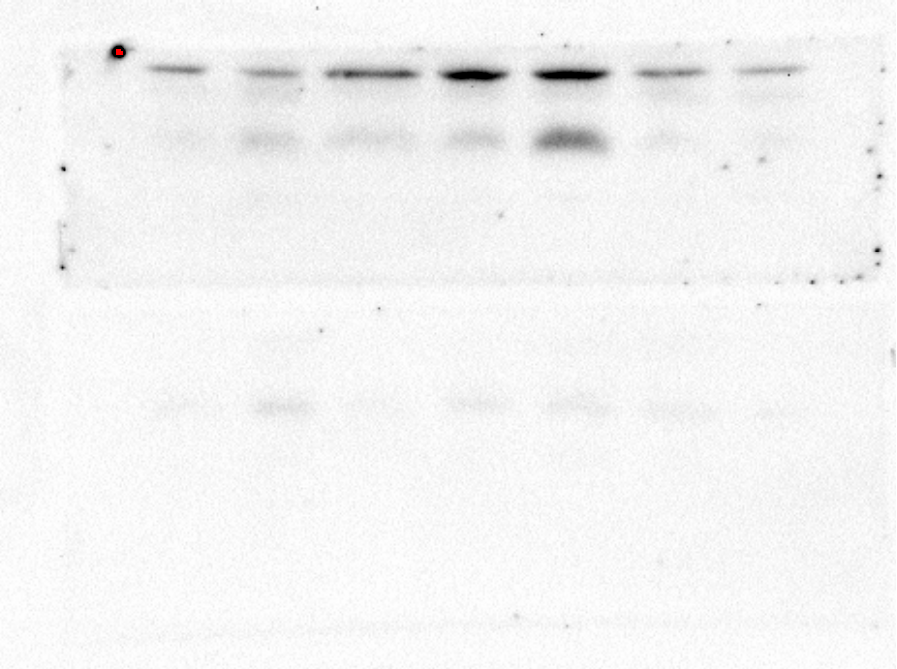

Supplement: Supplementary file 1 [file Data_Sheet_1.ZIP › Original Files of WB bands/trem2 timepoints.tif]

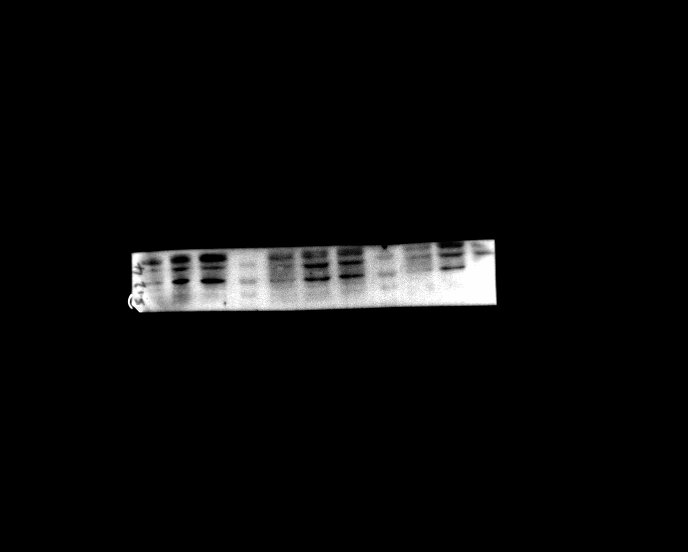

Supplement: Supplementary file 1 [file Data_Sheet_1.ZIP › Original Files of WB bands/trem2overexpression.tif]

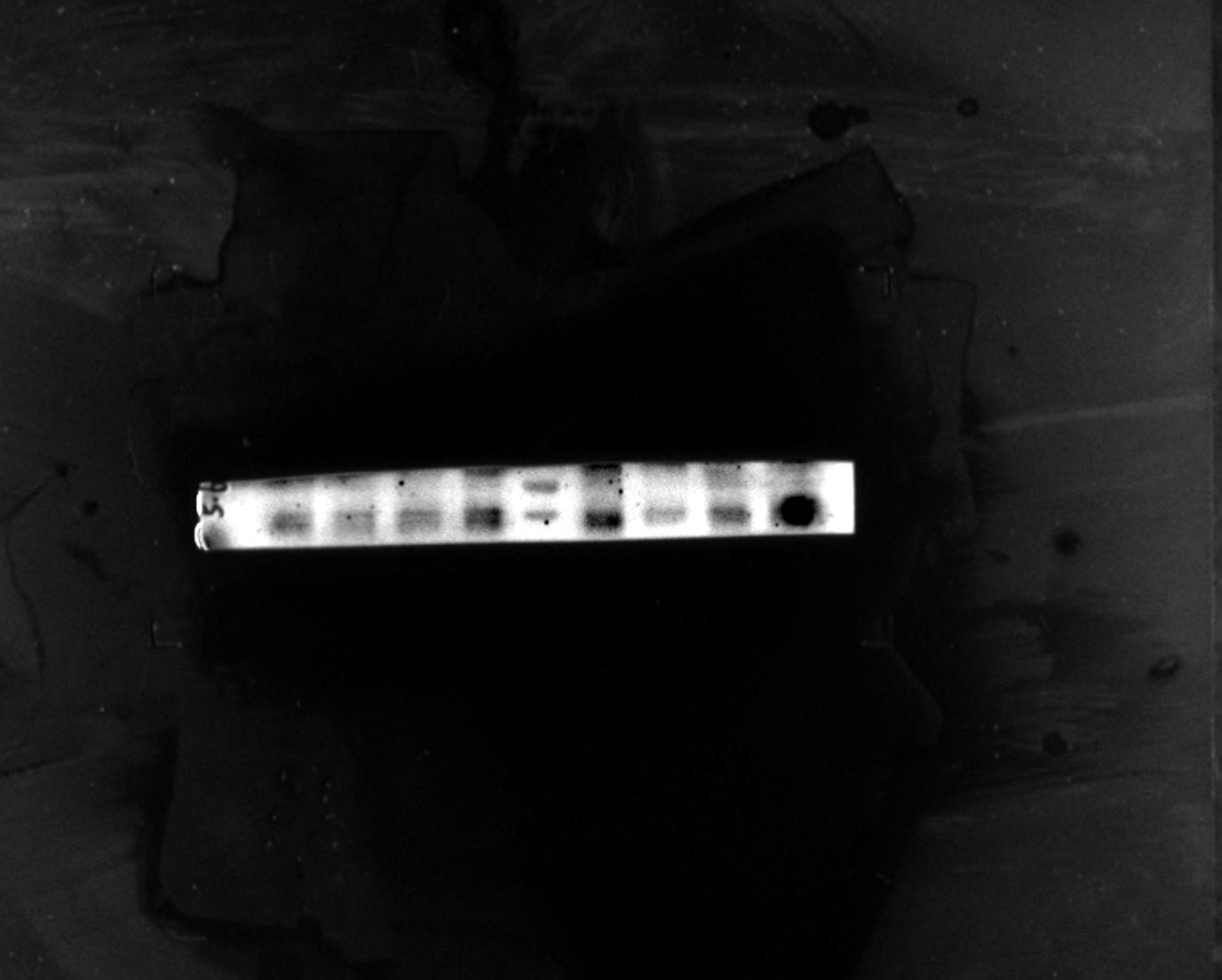

Supplement: Supplementary file 1 [file Data_Sheet_1.ZIP › Original Files of WB bands/TREM2(following Oxygen).Tif]

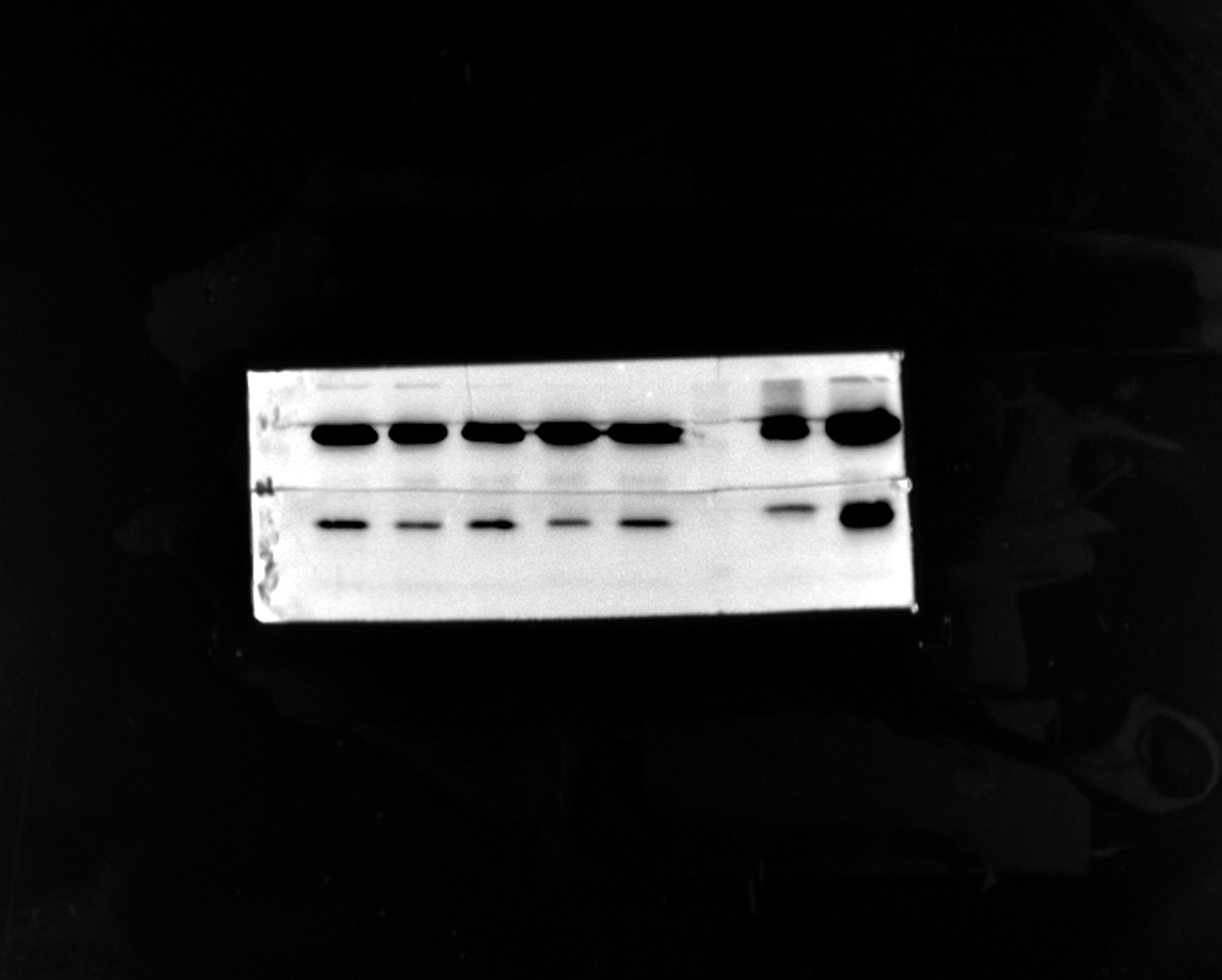

Supplement: Supplementary file 1 [file Data_Sheet_1.ZIP › Original Files of WB bands/tubulin and bcl2.Tif]

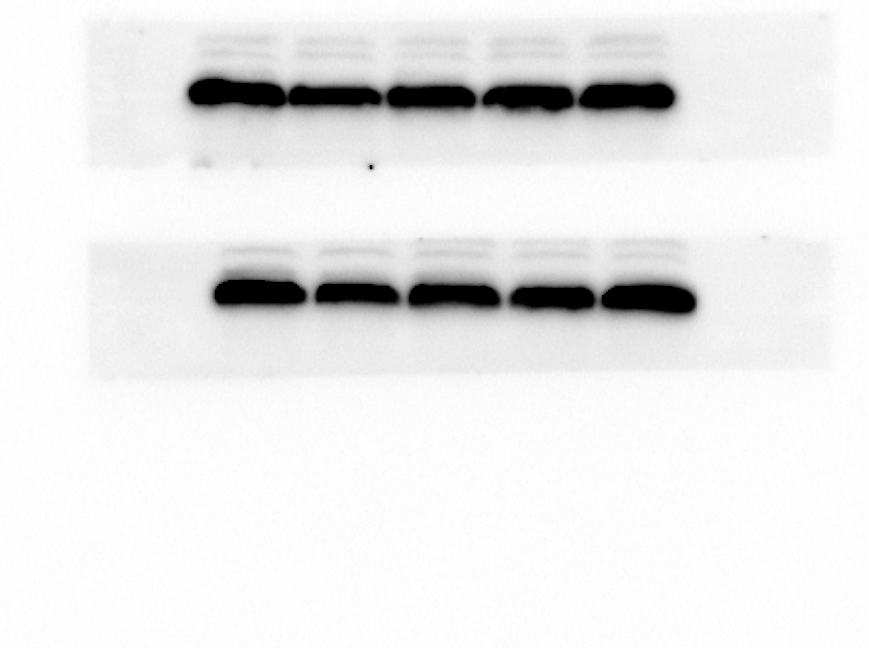

Supplement: Supplementary file 1 [file Data_Sheet_1.ZIP › Original Files of WB bands/tubulin of tlr4.tif]

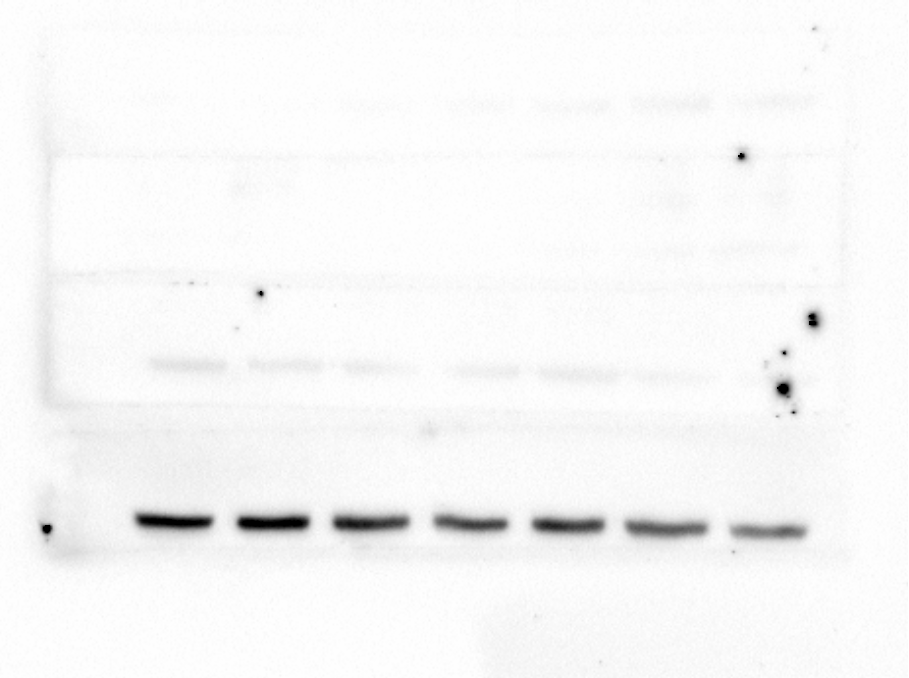

Supplement: Supplementary file 1 [file Data_Sheet_1.ZIP › Original Files of WB bands/tubulin of TREM2 timepoints.tif]

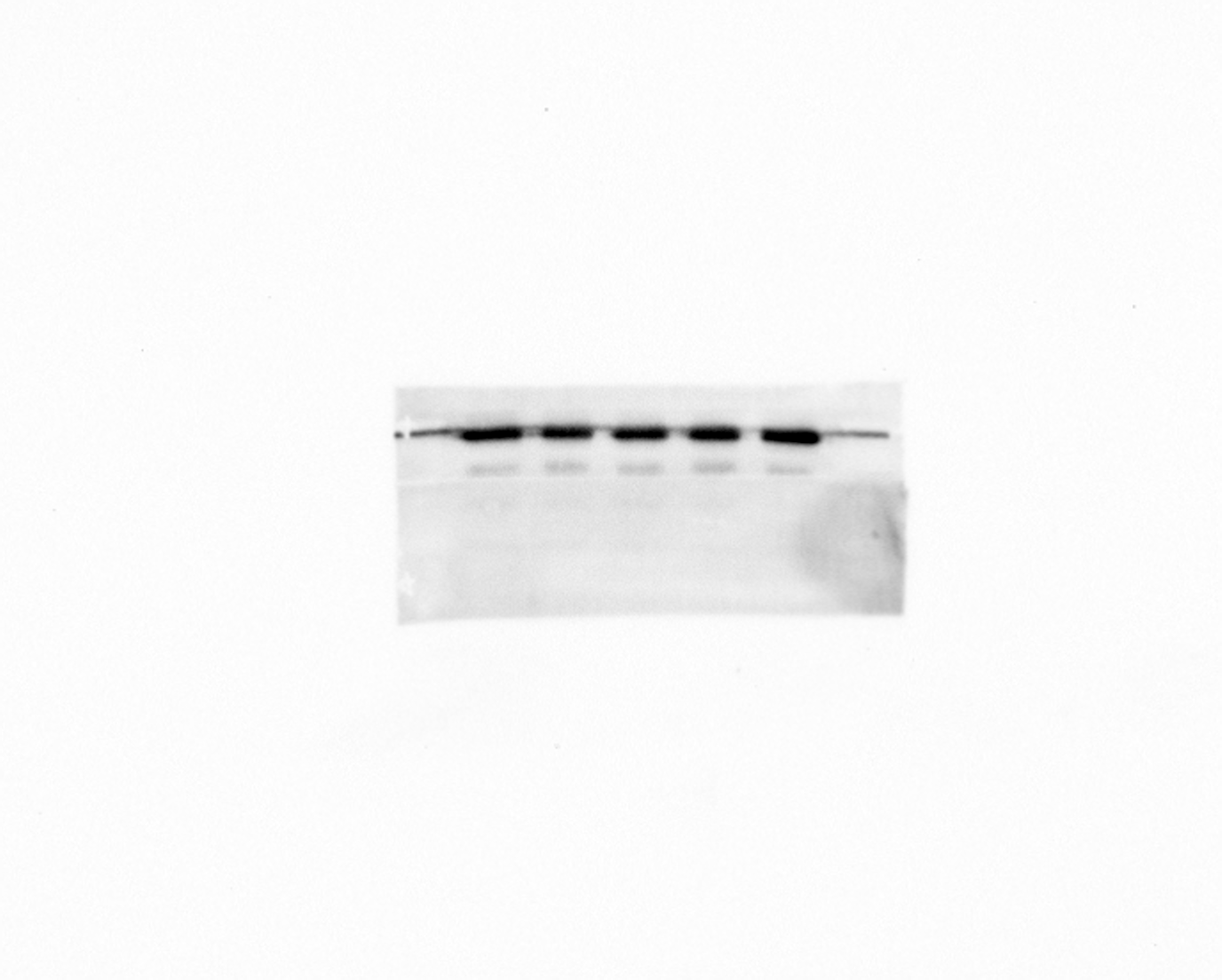

Supplement: Supplementary file 1 [file Data_Sheet_1.ZIP › Original Files of WB bands/tubulin.Tif]

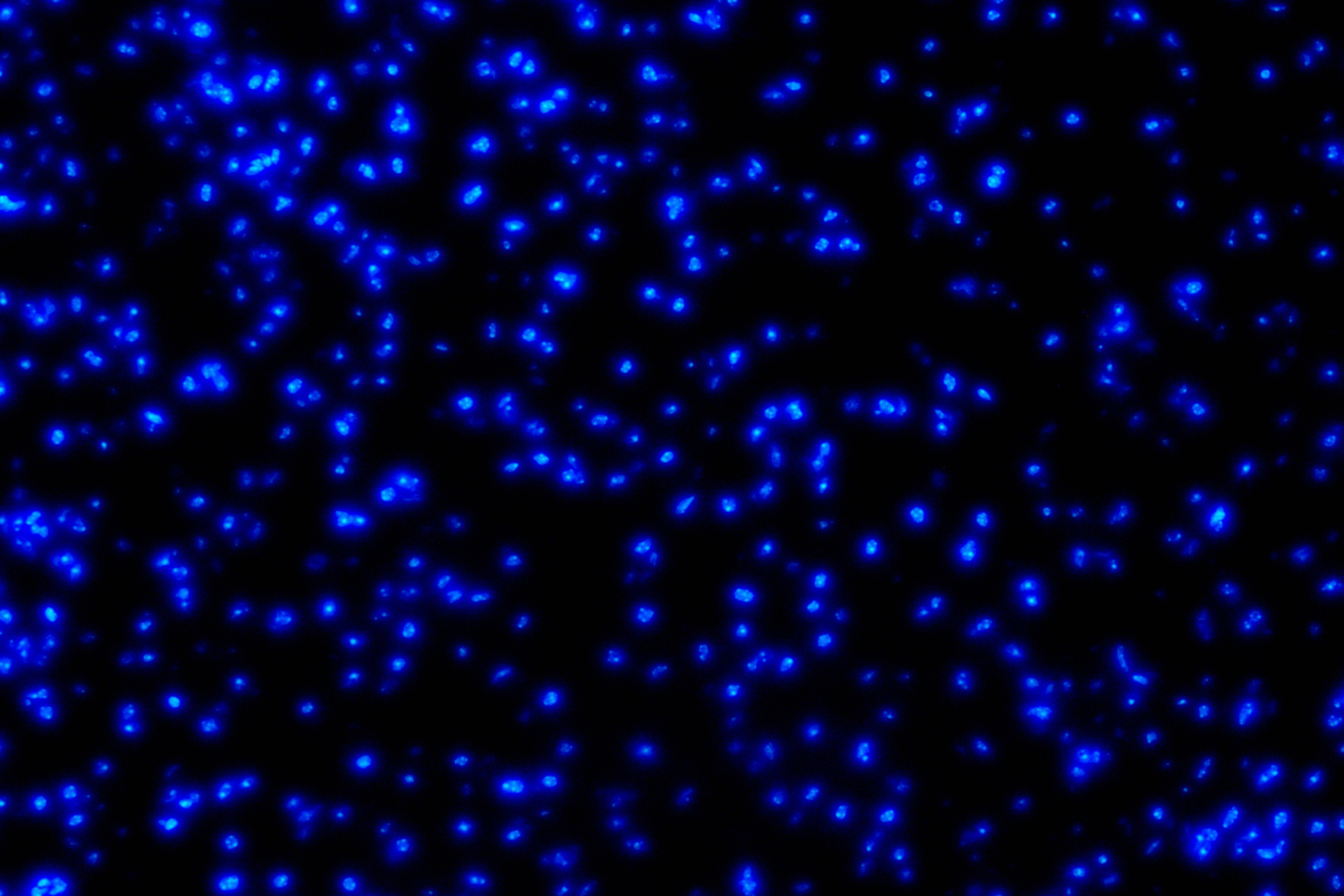

Supplement: Supplementary file 2 [file Data_Sheet_2.ZIP › Original Files of Tunel Images/AAV-NC-dapi.jpg]

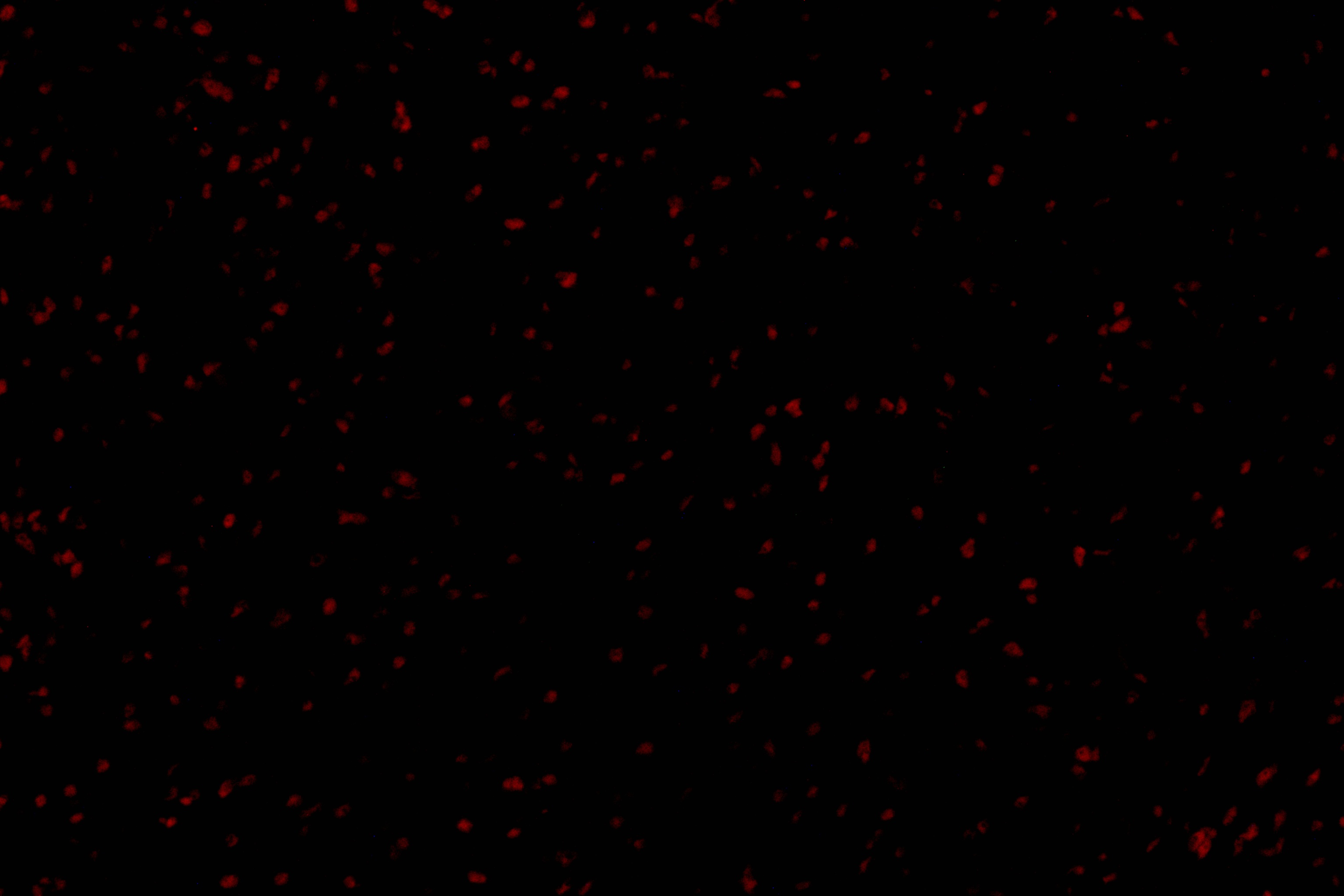

Supplement: Supplementary file 2 [file Data_Sheet_2.ZIP › Original Files of Tunel Images/AAV-NC.jpg]

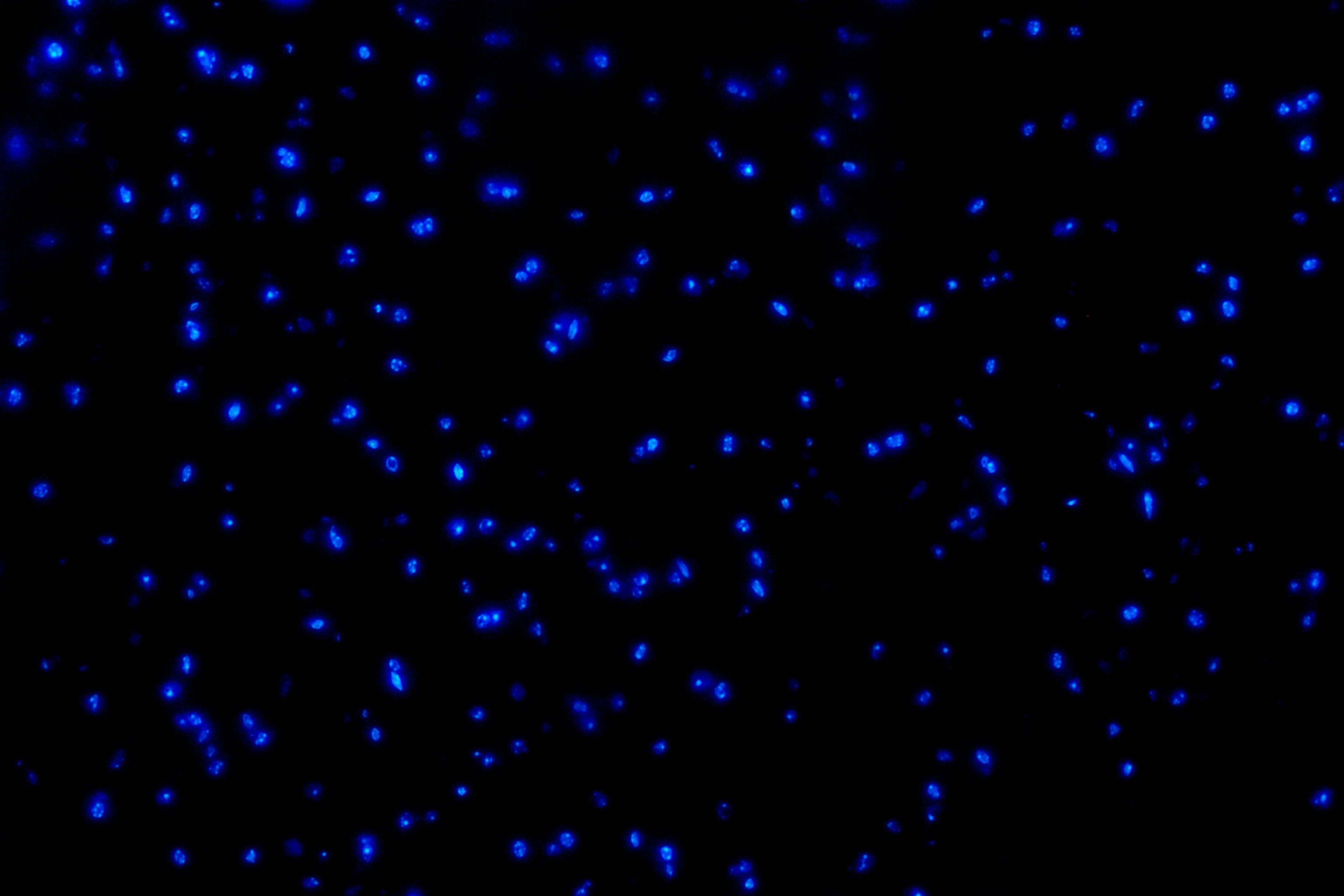

Supplement: Supplementary file 2 [file Data_Sheet_2.ZIP › Original Files of Tunel Images/AAV-TREM2-dapi.jpg]

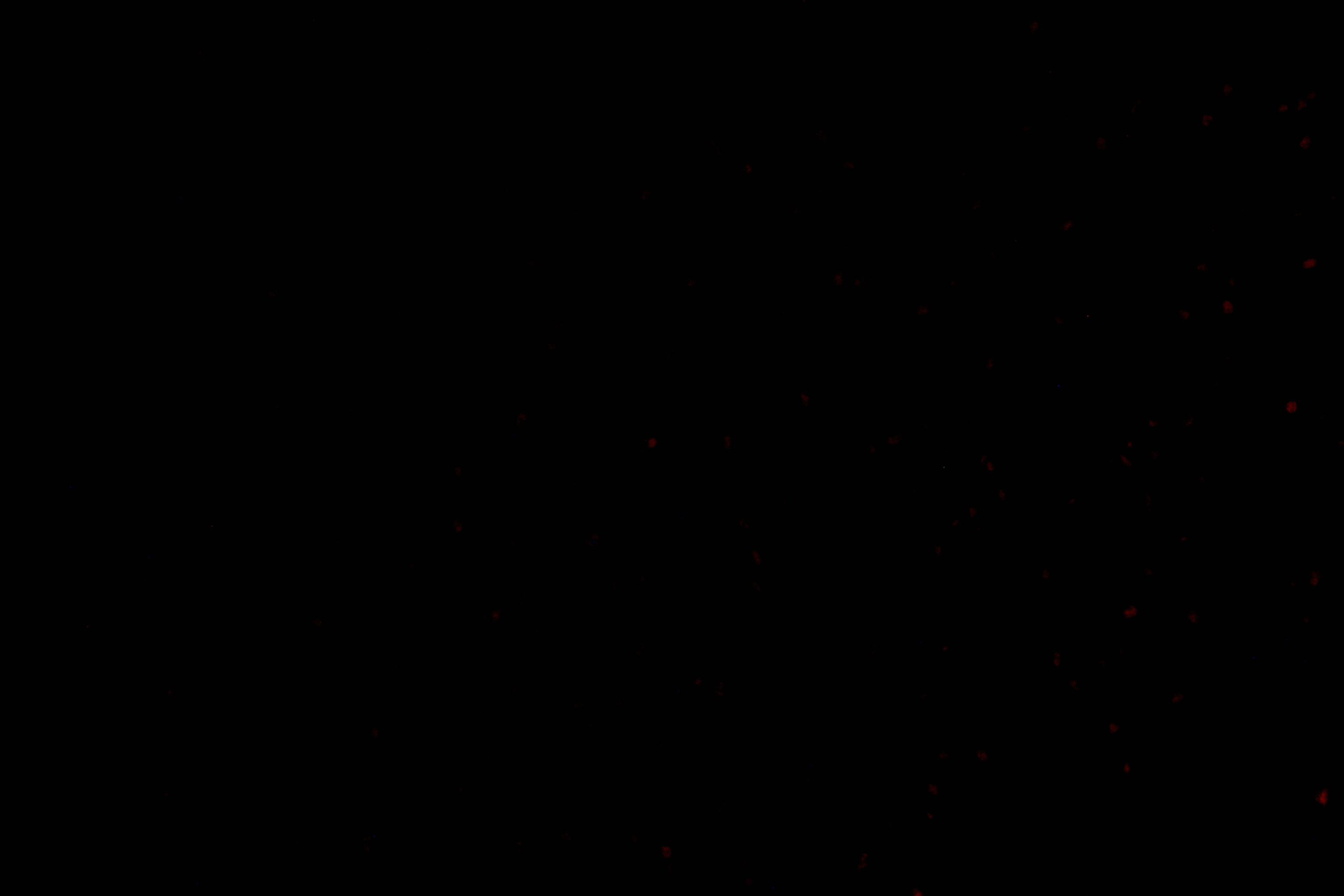

Supplement: Supplementary file 2 [file Data_Sheet_2.ZIP › Original Files of Tunel Images/AAV-TREM2.jpg]

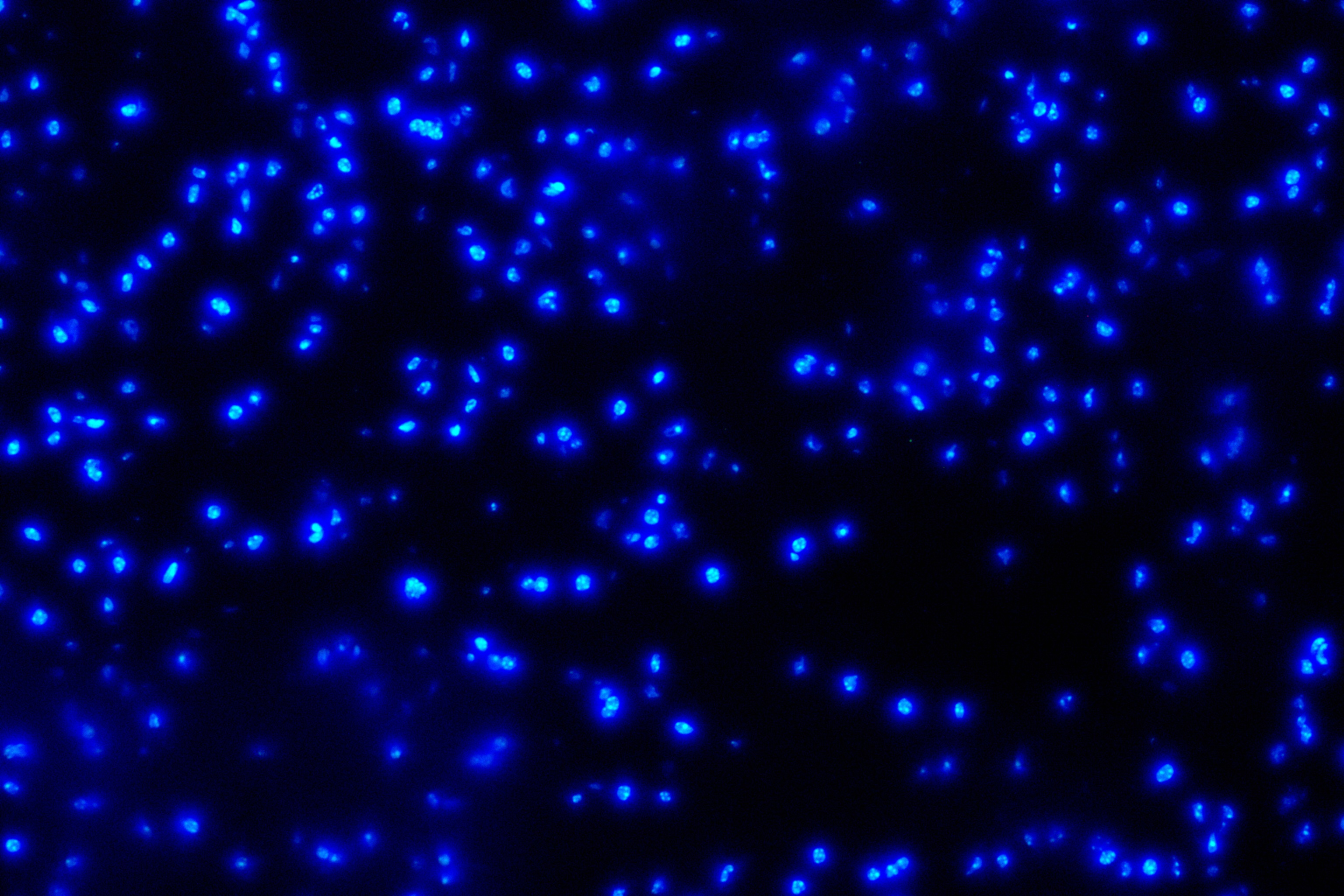

Supplement: Supplementary file 2 [file Data_Sheet_2.ZIP › Original Files of Tunel Images/sham-dapi.jpg]

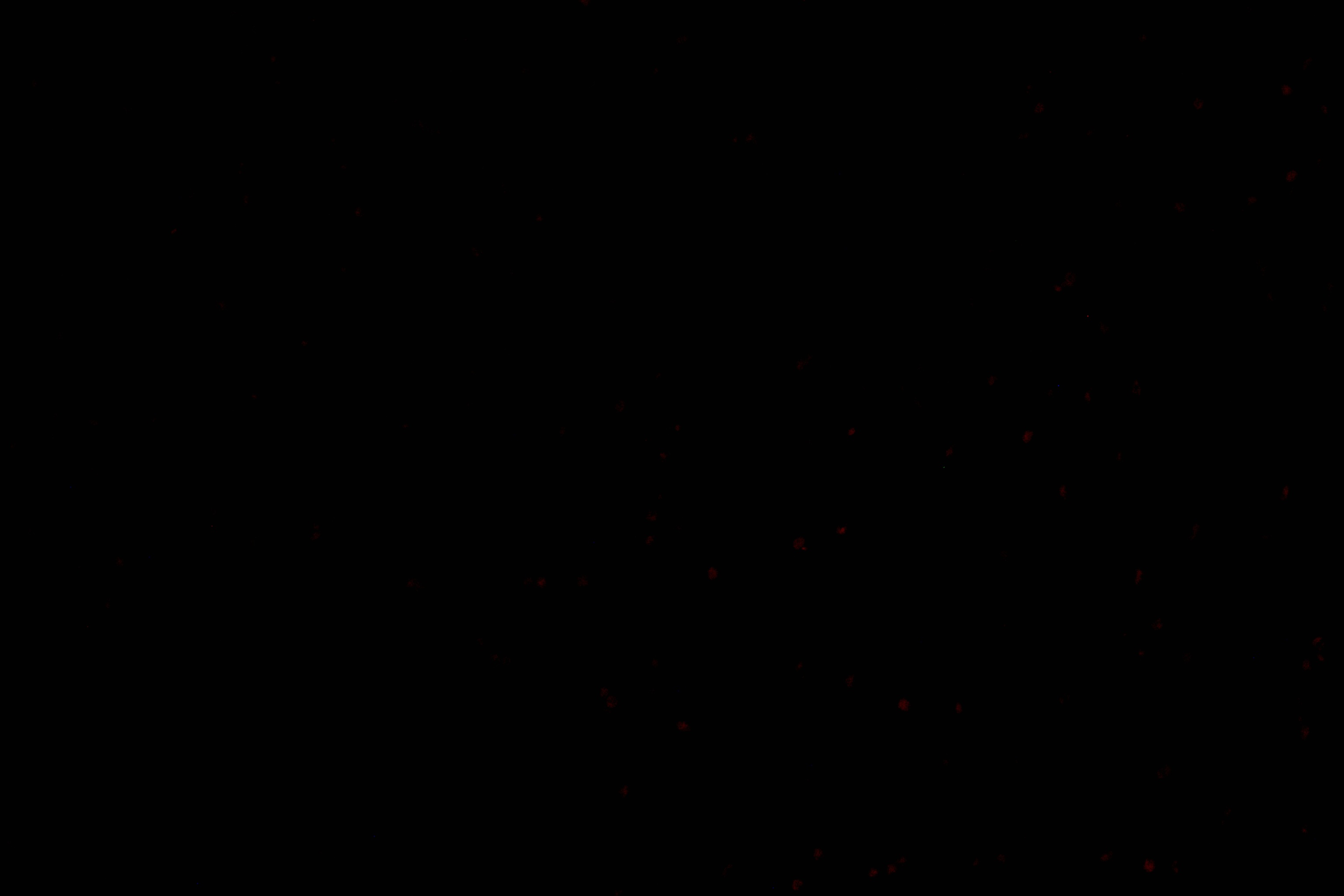

Supplement: Supplementary file 2 [file Data_Sheet_2.ZIP › Original Files of Tunel Images/sham.jpg]

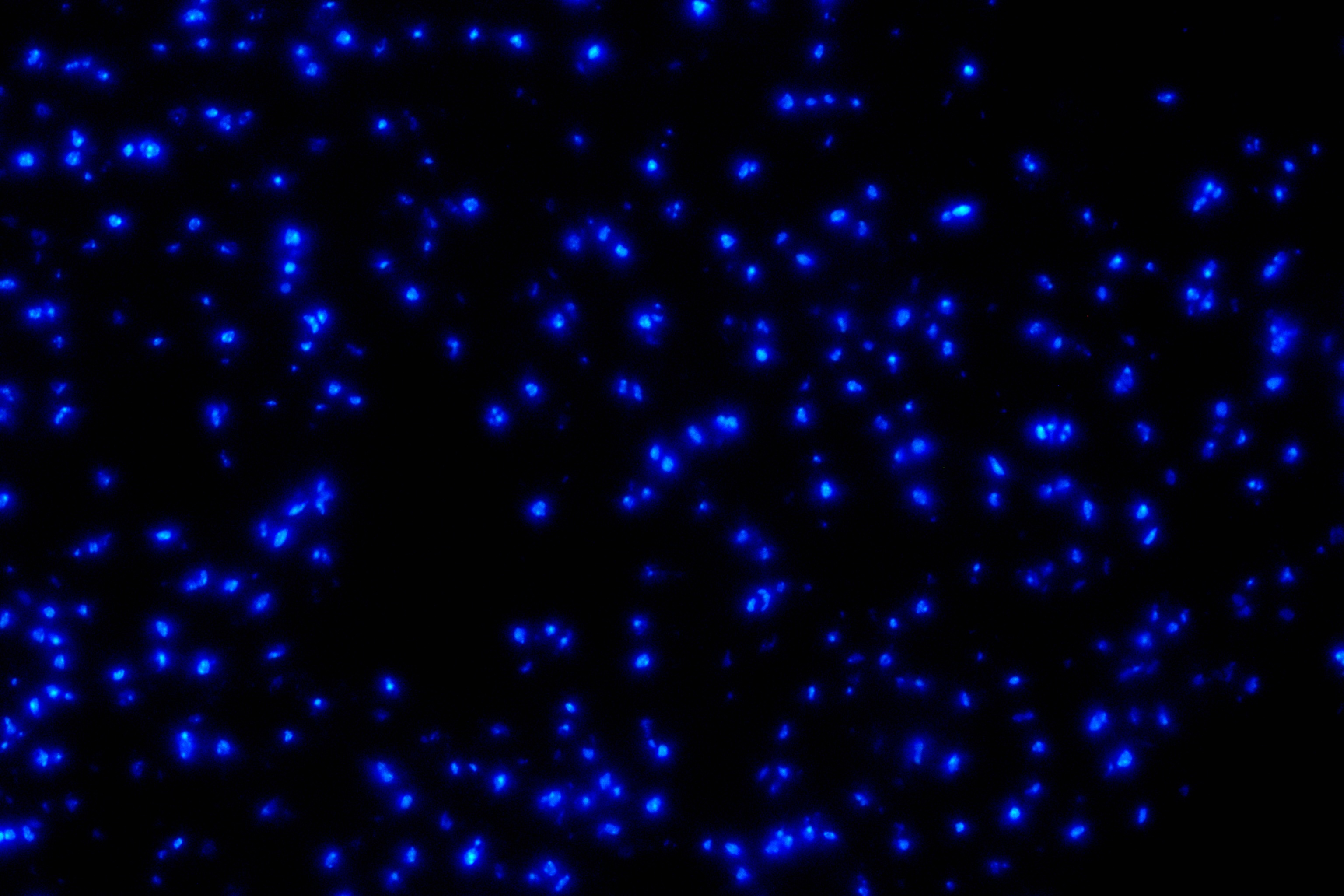

Supplement: Supplementary file 2 [file Data_Sheet_2.ZIP › Original Files of Tunel Images/tak242-dapi.jpg]

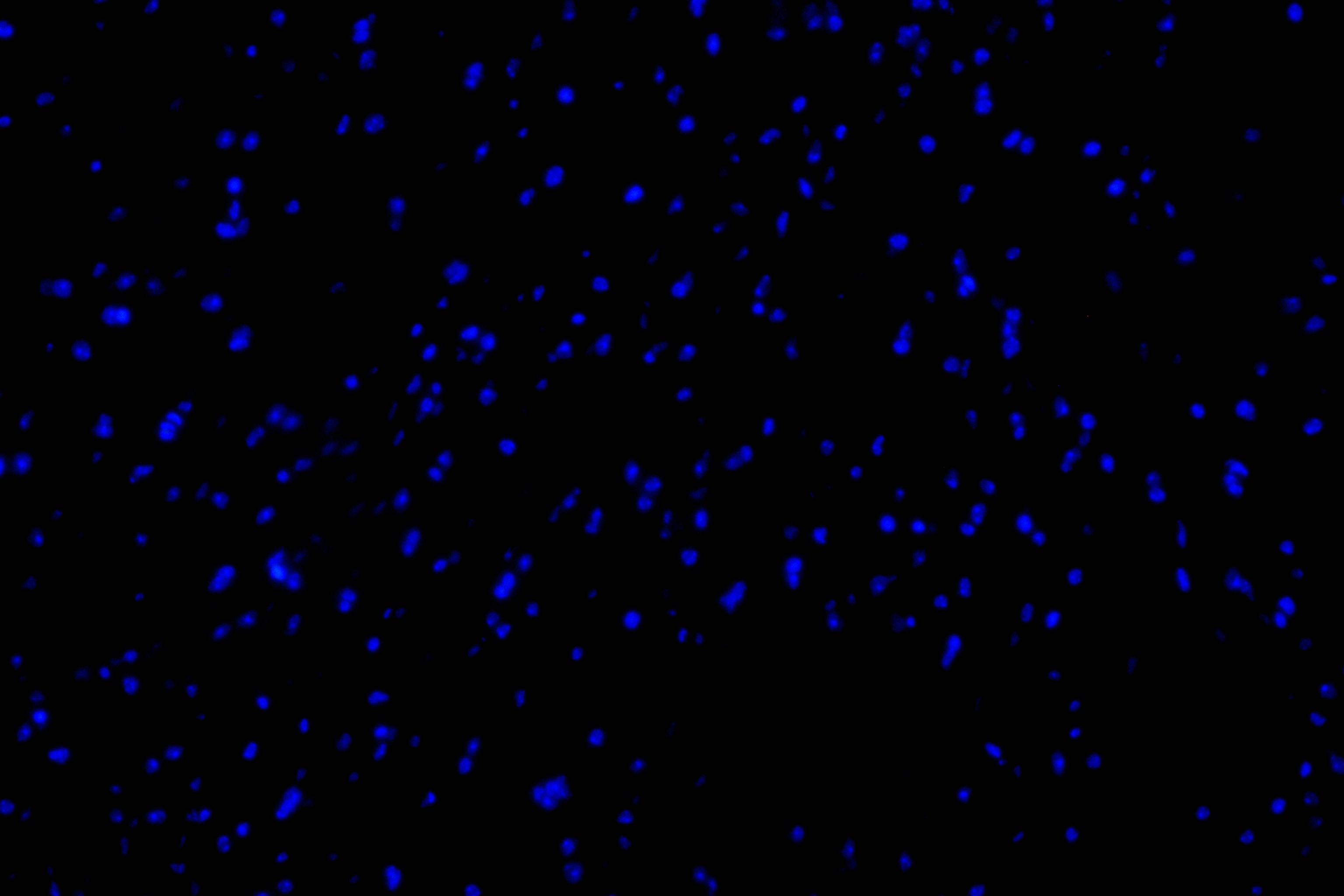

Supplement: Supplementary file 2 [file Data_Sheet_2.ZIP › Original Files of Tunel Images/TAK242-NC-dapi.jpg]

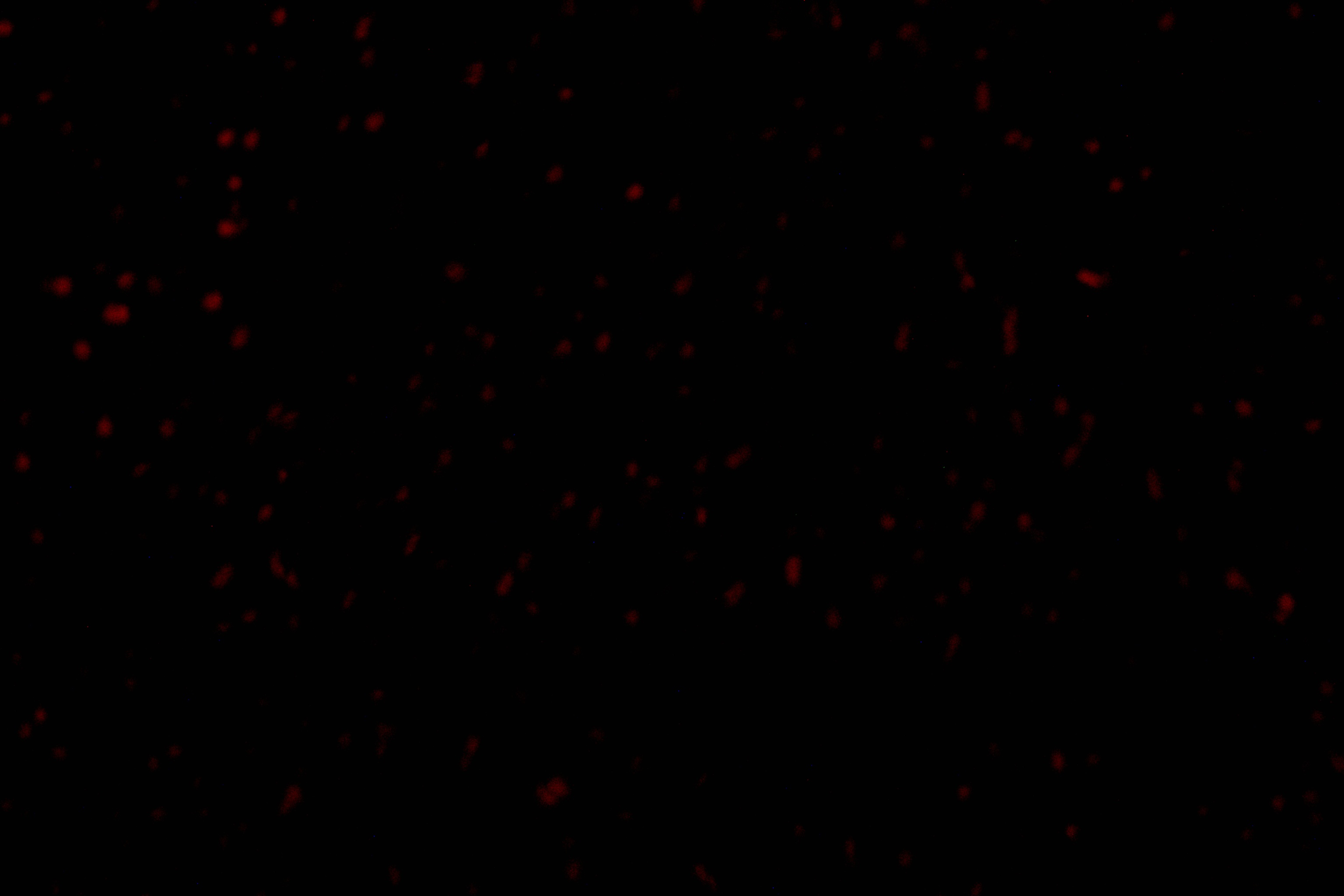

Supplement: Supplementary file 2 [file Data_Sheet_2.ZIP › Original Files of Tunel Images/TAK242-NC.jpg]

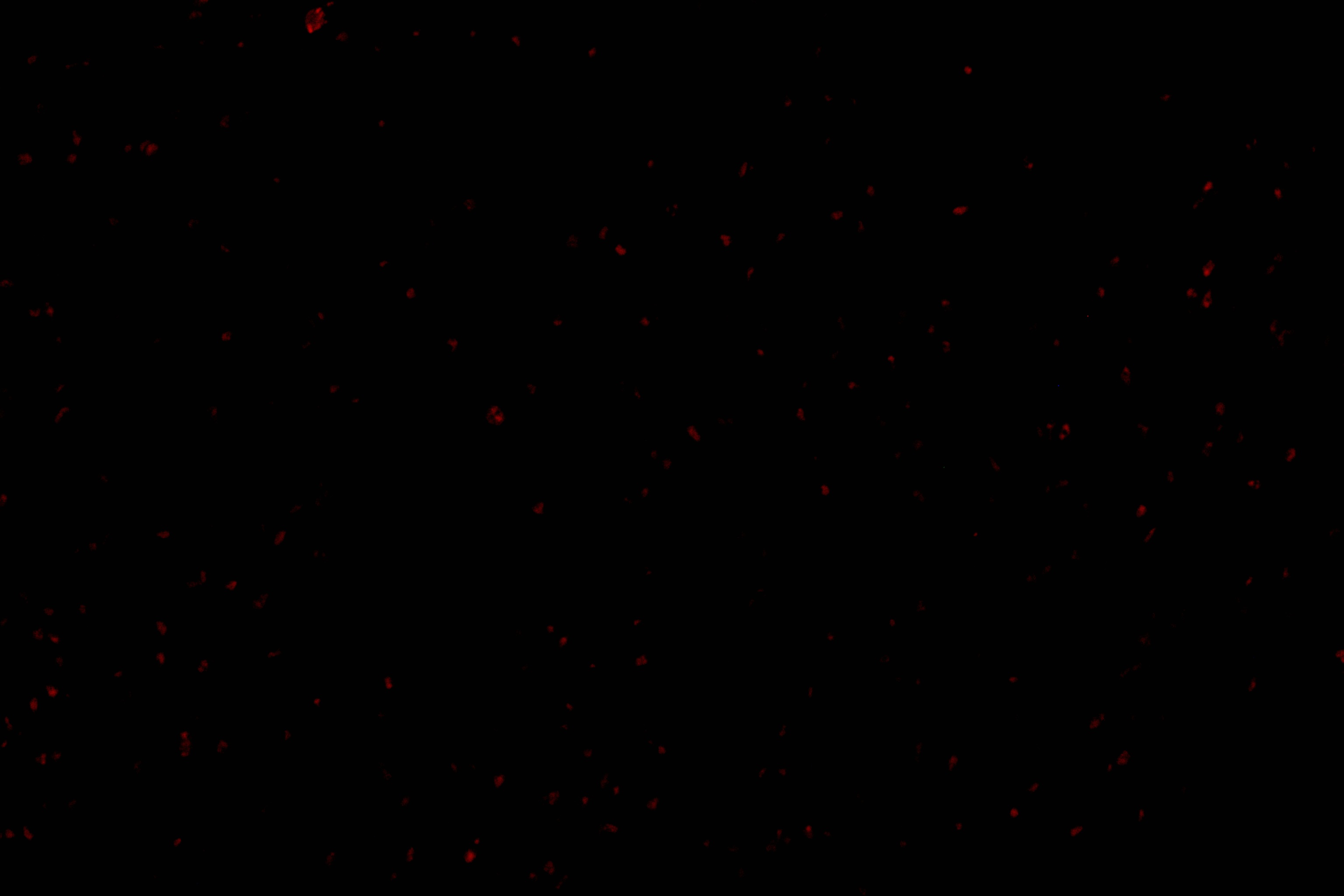

Supplement: Supplementary file 2 [file Data_Sheet_2.ZIP › Original Files of Tunel Images/TAK242.jpg]

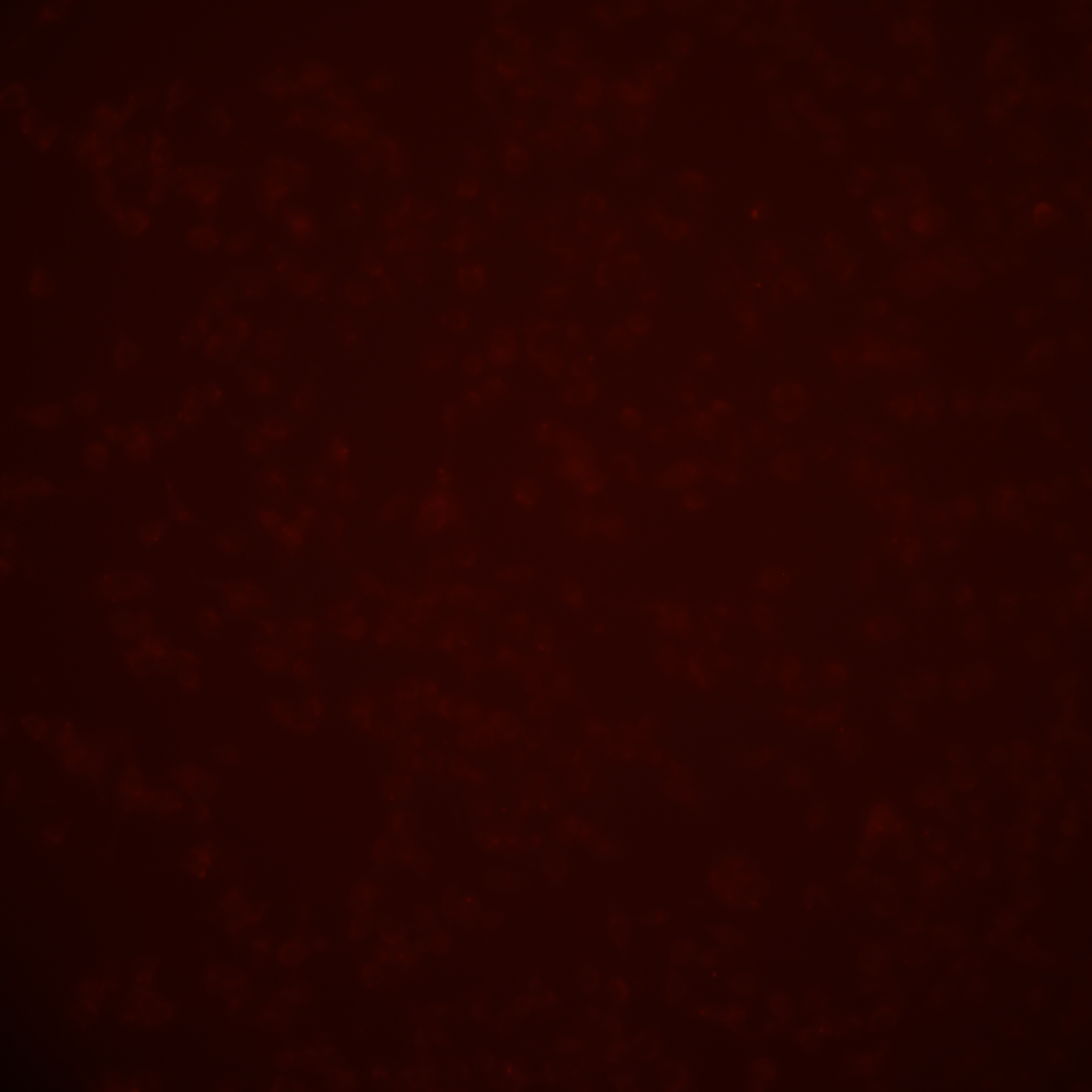

Supplement: Supplementary file 3 [file Data_Sheet_3.ZIP › Immunochemical staining of TLR4/1-BV2-NC.tif]

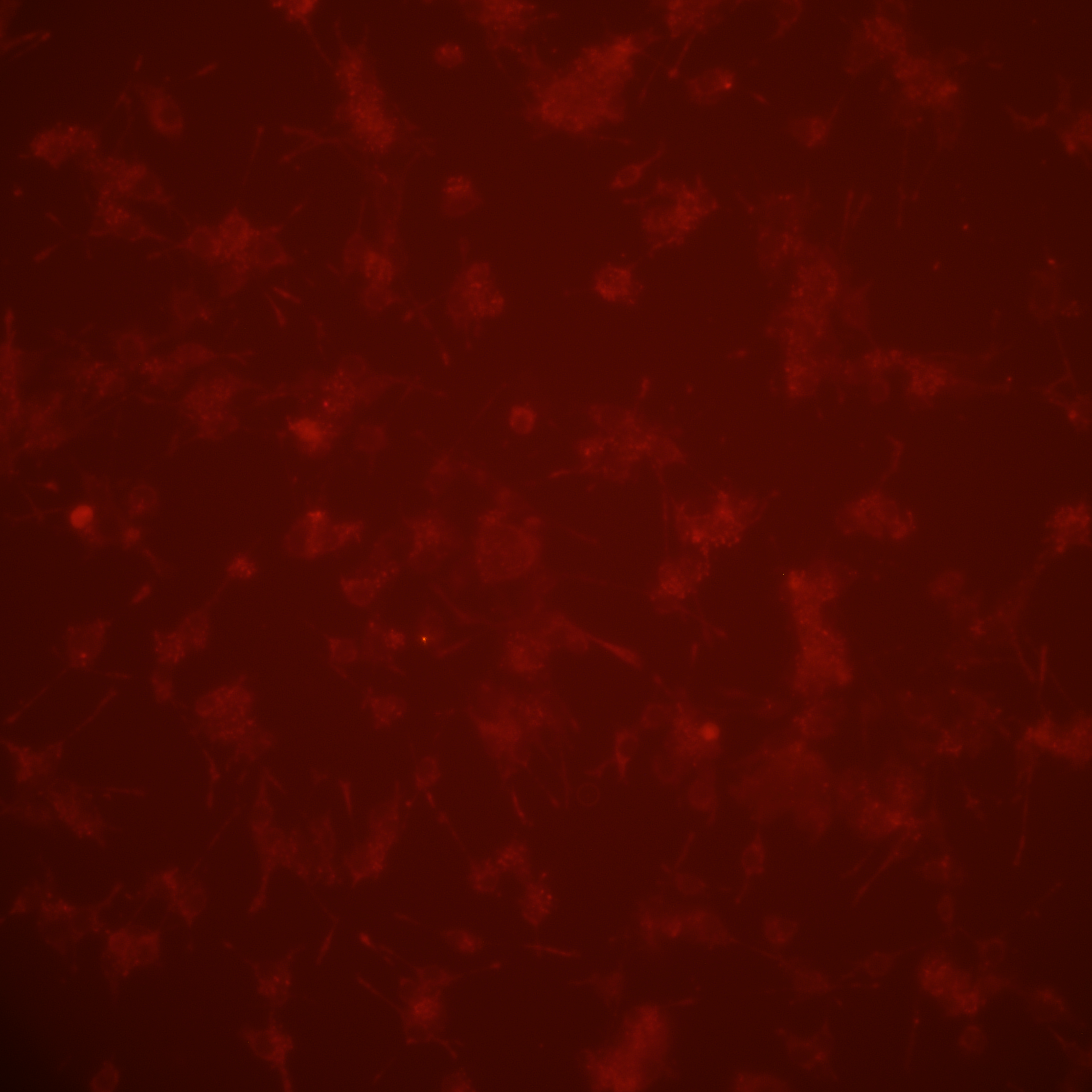

Supplement: Supplementary file 3 [file Data_Sheet_3.ZIP › Immunochemical staining of TLR4/2-BV2-Oxygen.tif]

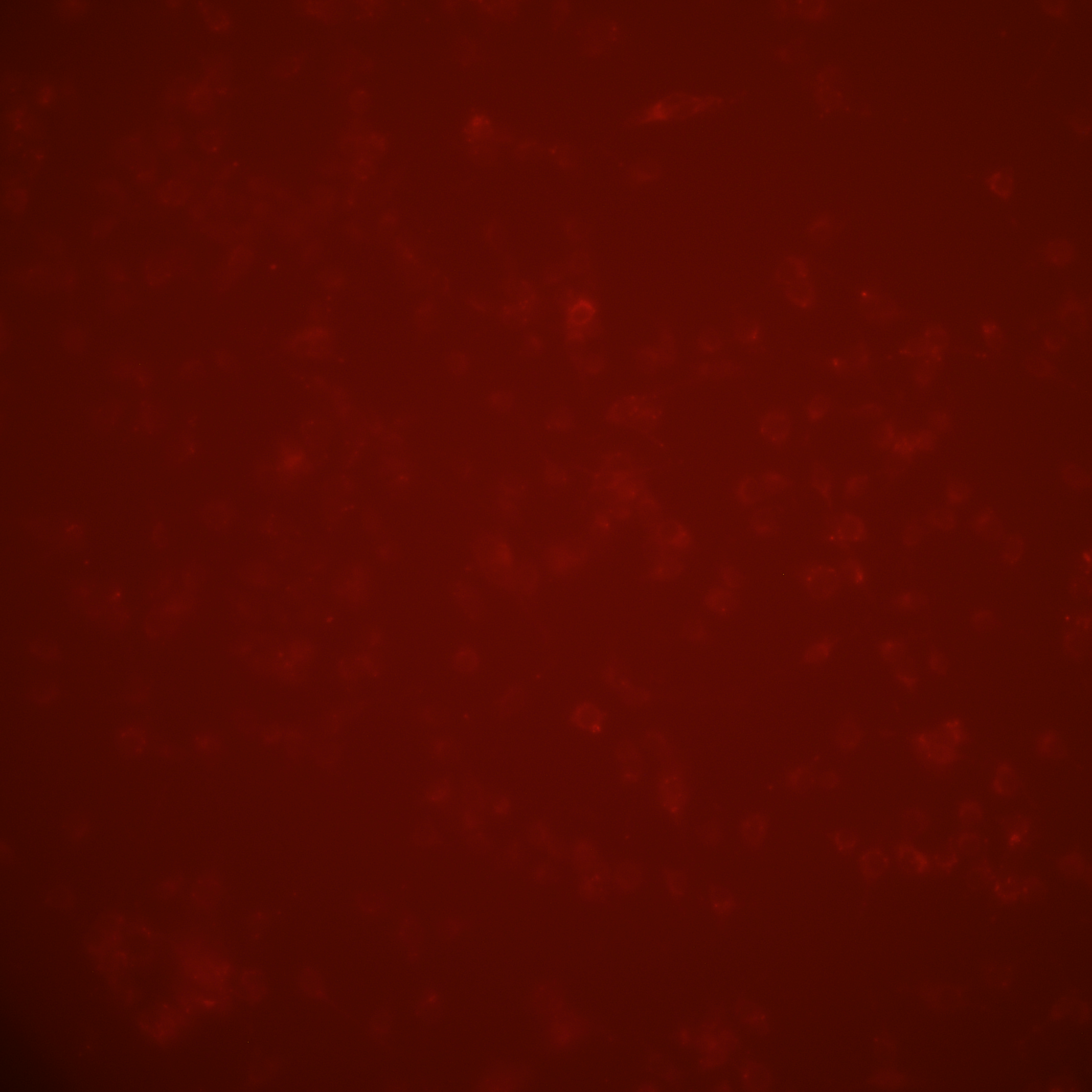

Supplement: Supplementary file 3 [file Data_Sheet_3.ZIP › Immunochemical staining of TLR4/3-BV2-Oxygen+TAK242.tif]

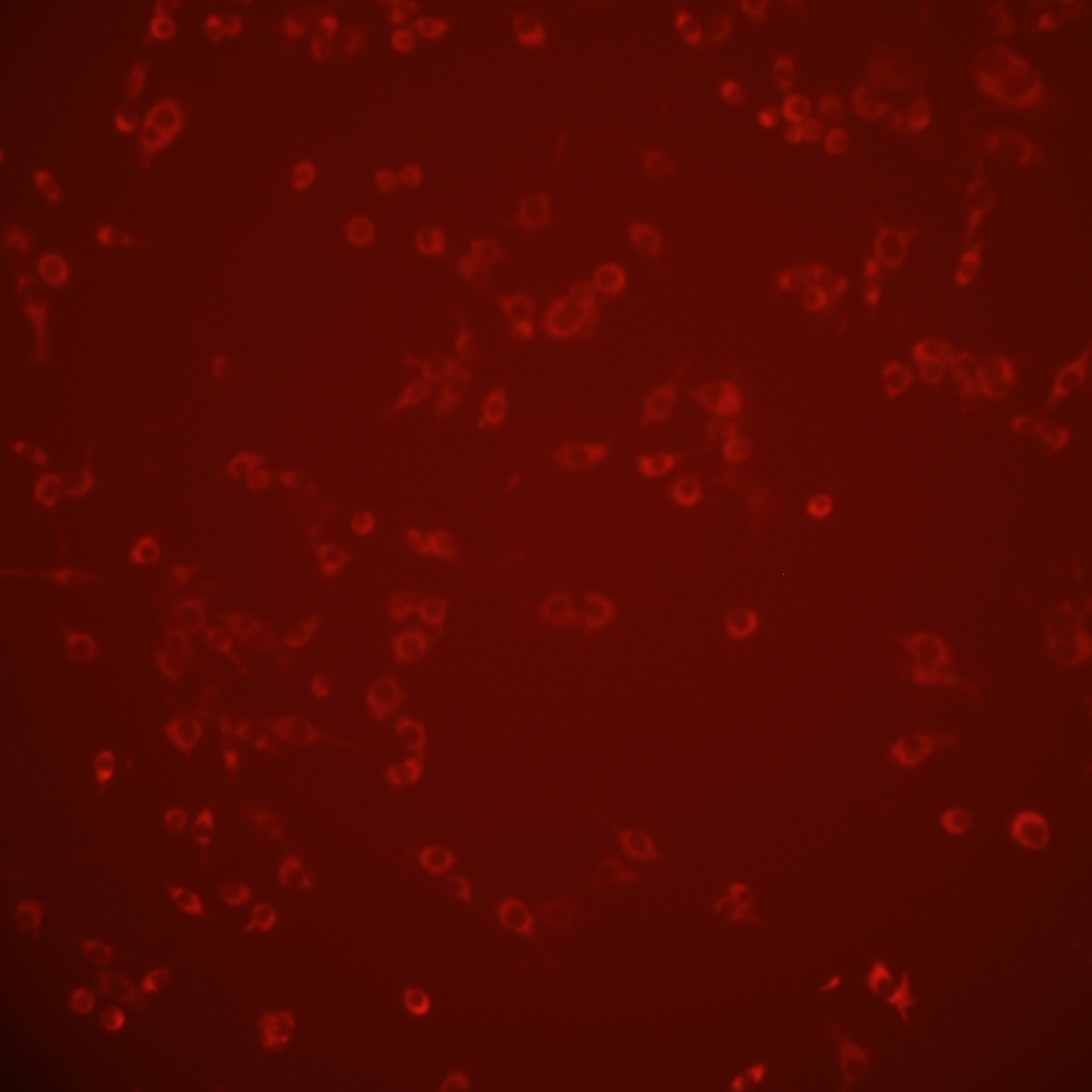

Supplement: Supplementary file 3 [file Data_Sheet_3.ZIP › Immunochemical staining of TLR4/4-LV-TREM2-NC.tif]

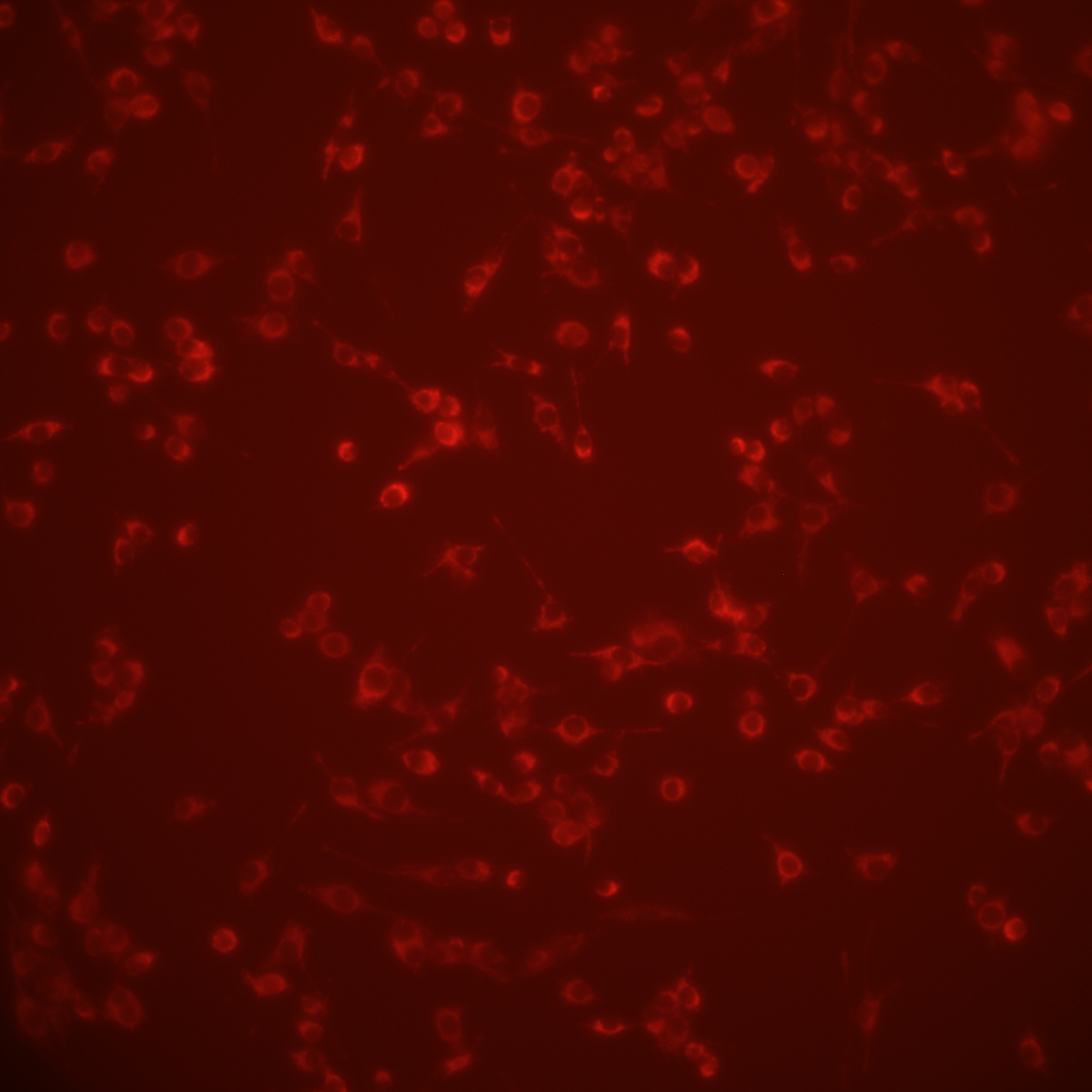

Supplement: Supplementary file 3 [file Data_Sheet_3.ZIP › Immunochemical staining of TLR4/5-LV-TREM2-Oxygen.tif]

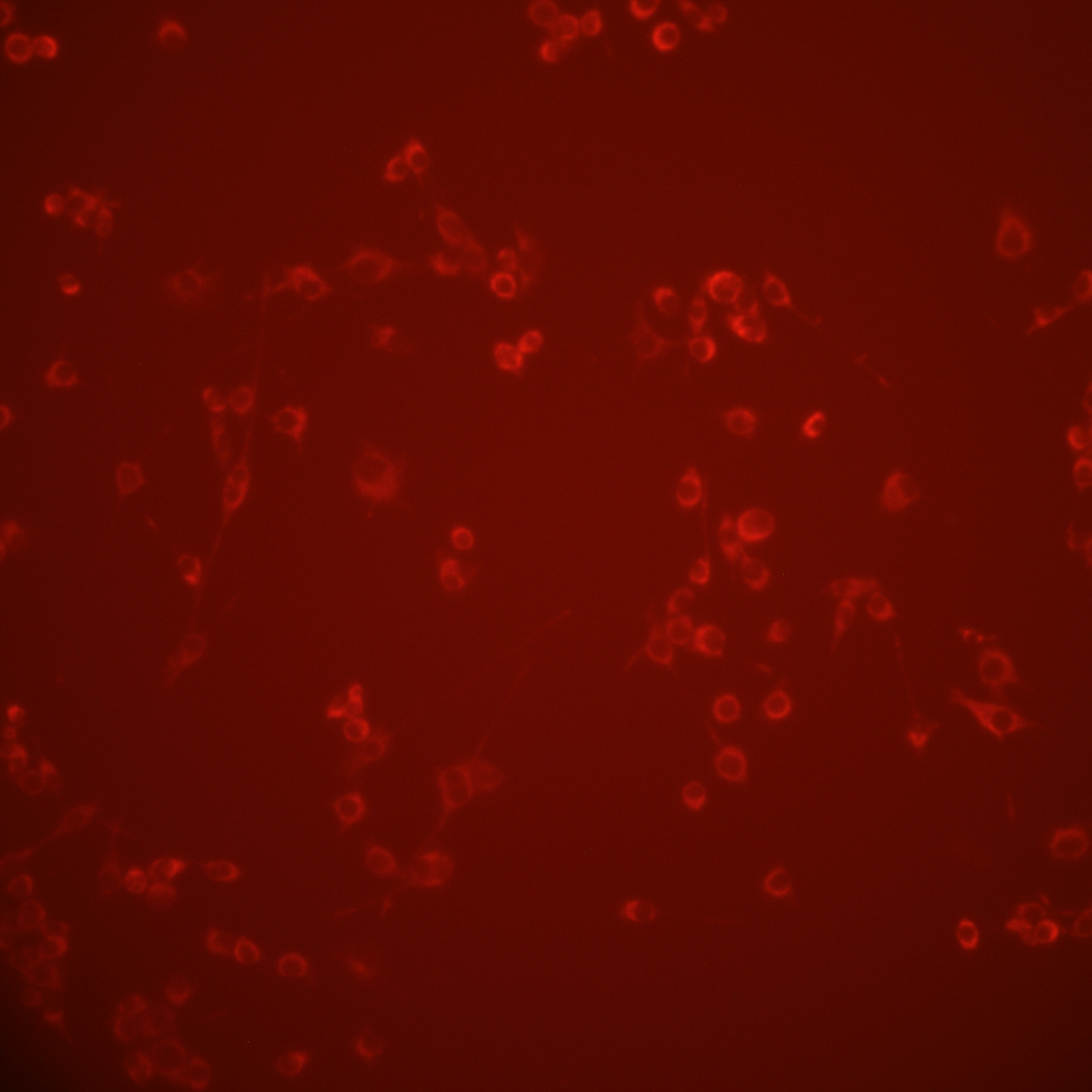

Supplement: Supplementary file 3 [file Data_Sheet_3.ZIP › Immunochemical staining of TLR4/6-LV-TREM2-Oxygen+TAK242.tif]

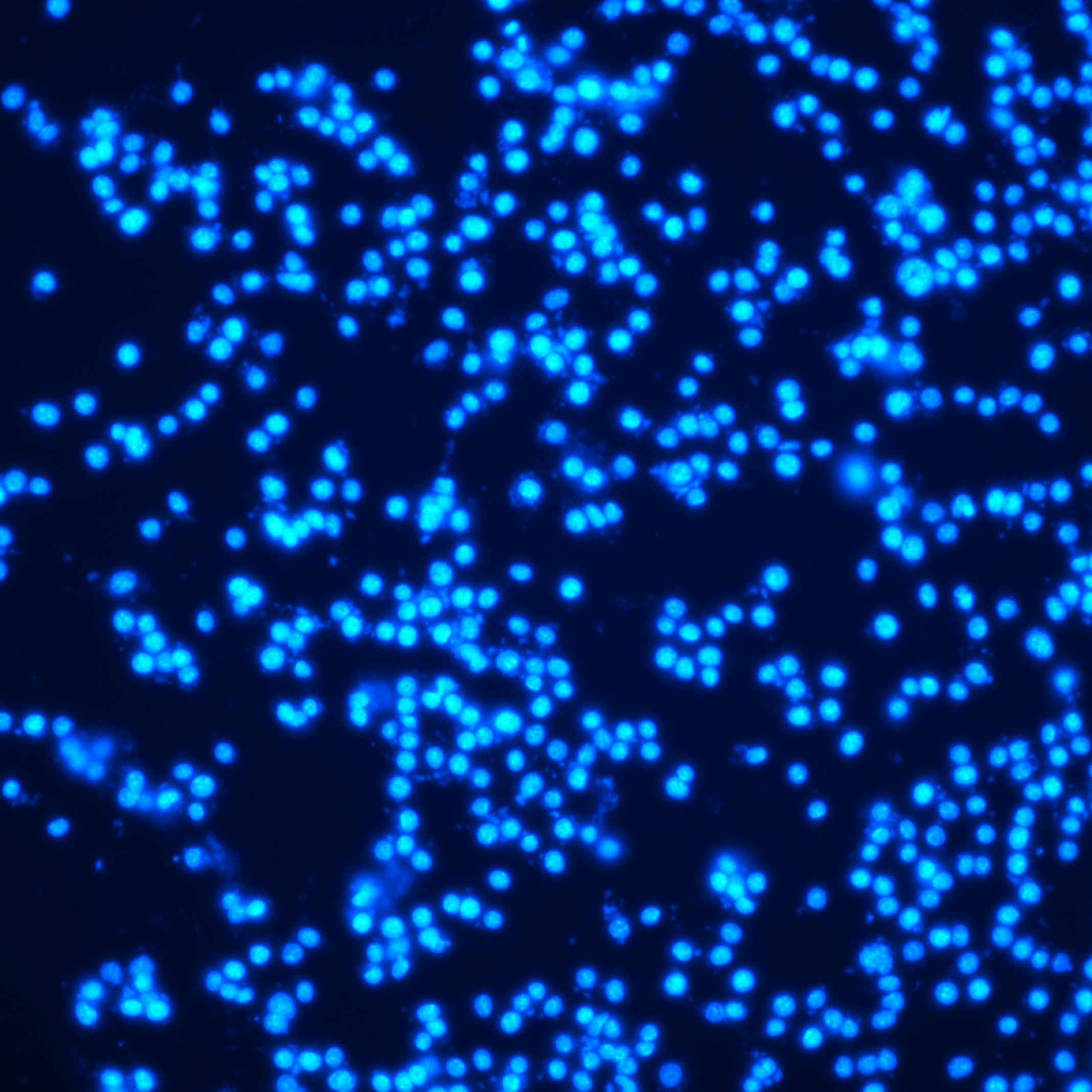

Supplement: Supplementary file 4 [file Data_Sheet_4.ZIP › Immunochemical staining of TLR4-DAPIs/1-BV2-NC-DAPI.tif]

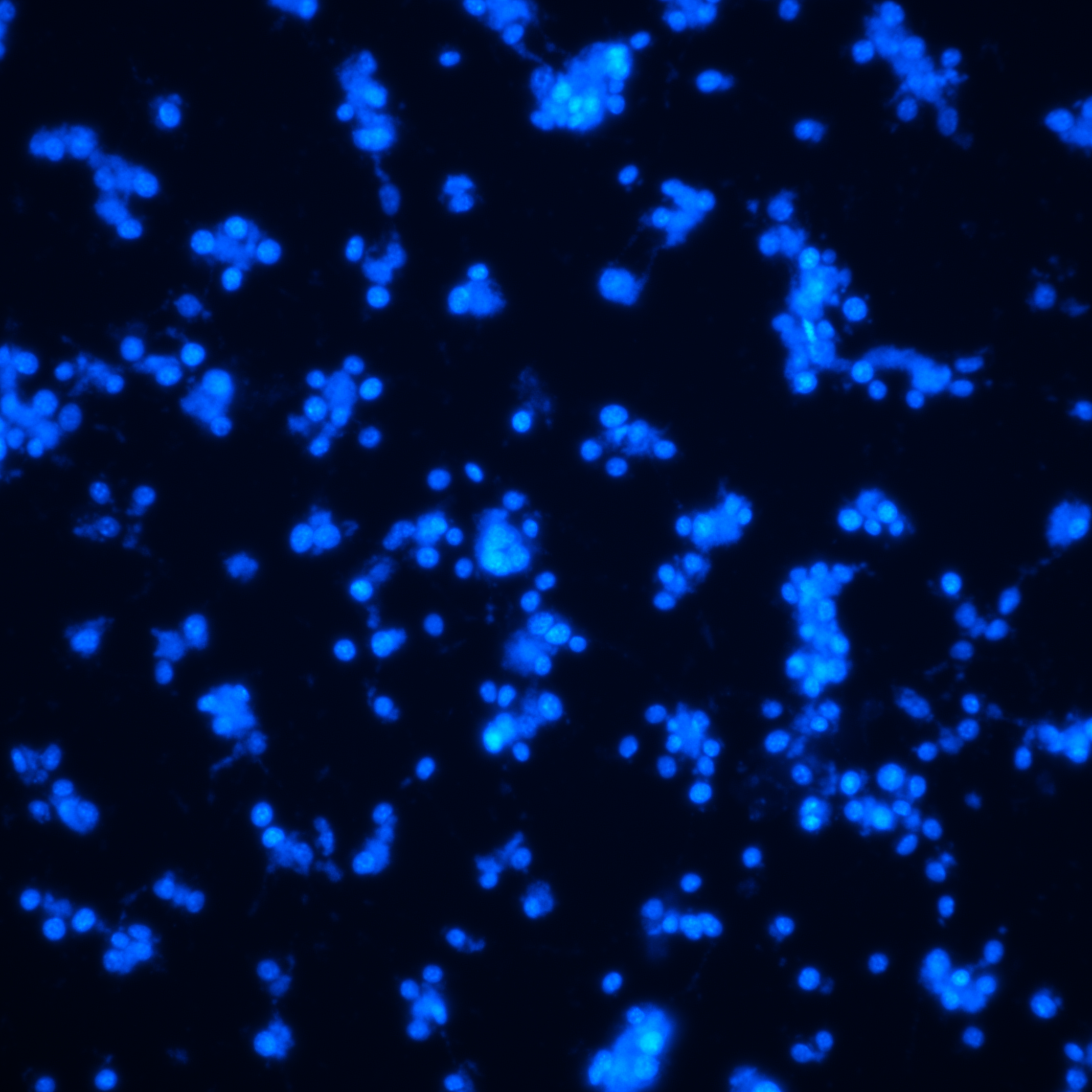

Supplement: Supplementary file 4 [file Data_Sheet_4.ZIP › Immunochemical staining of TLR4-DAPIs/2-BV2-Oxygen-DAPI.tif]

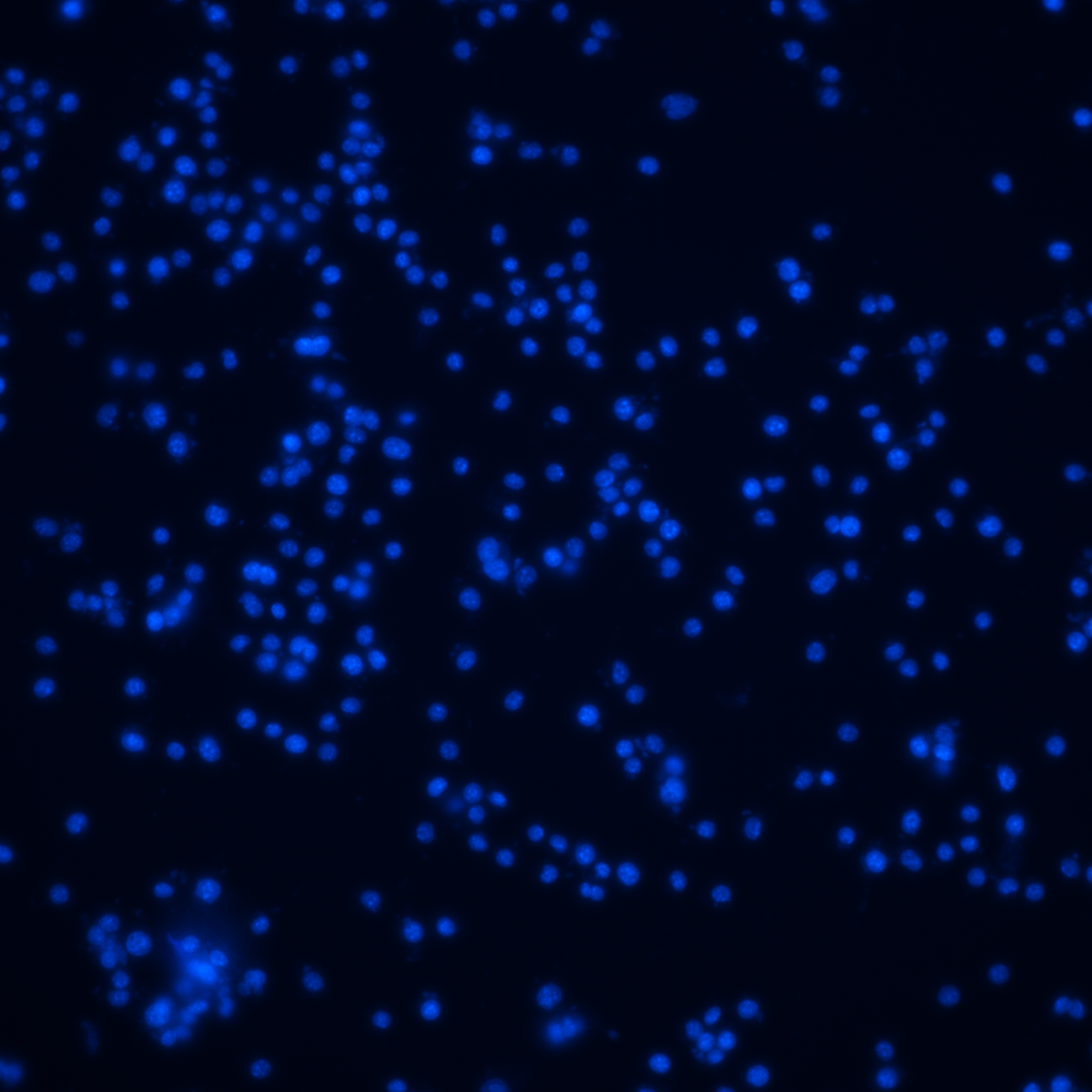

Supplement: Supplementary file 4 [file Data_Sheet_4.ZIP › Immunochemical staining of TLR4-DAPIs/3-BV2-Oxygen+TAK242-DAPI.tif]

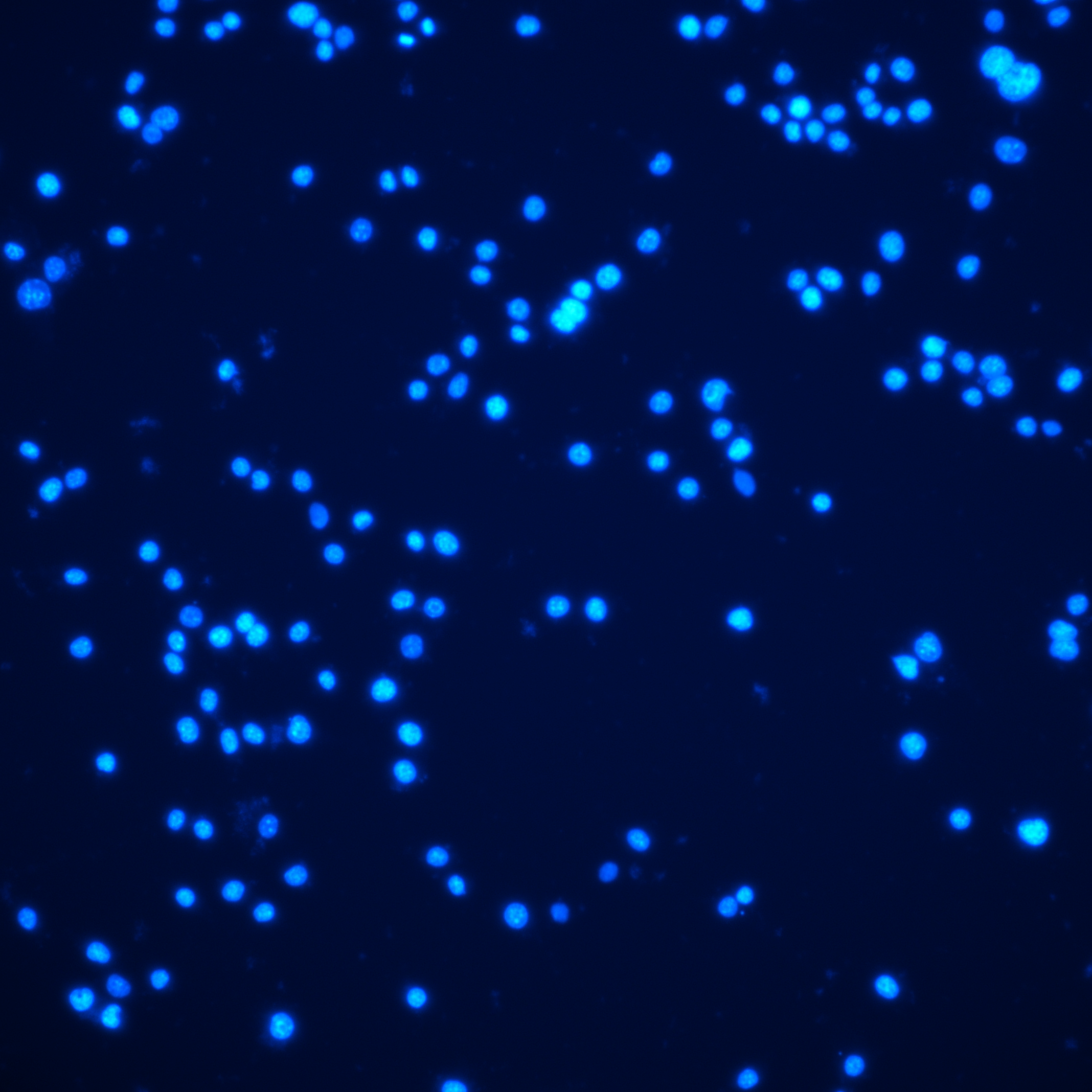

Supplement: Supplementary file 4 [file Data_Sheet_4.ZIP › Immunochemical staining of TLR4-DAPIs/4-LV-TREM2-NC-DAPI.tif]

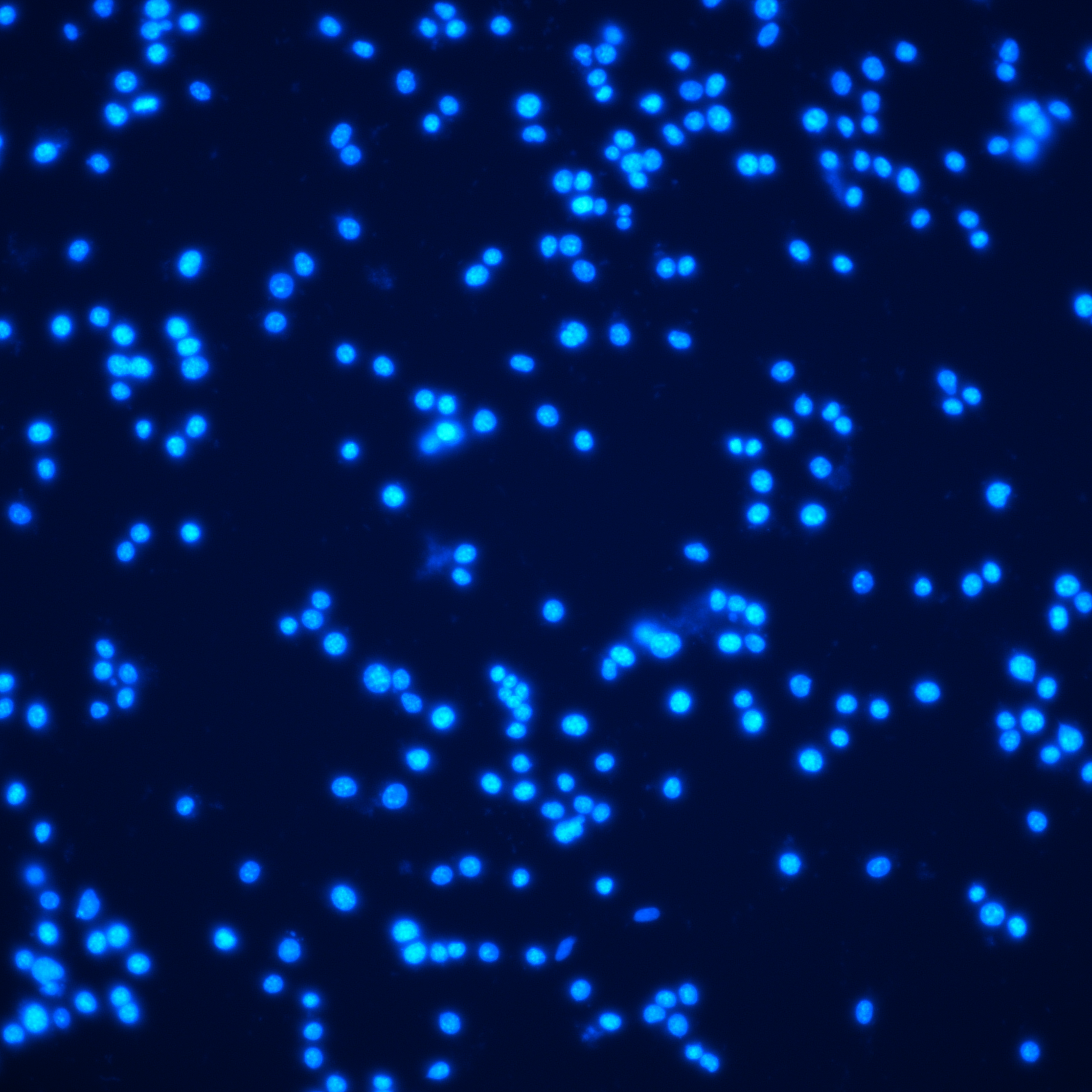

Supplement: Supplementary file 4 [file Data_Sheet_4.ZIP › Immunochemical staining of TLR4-DAPIs/5-LV-TREM2-Oxygen-DAPI.tif]

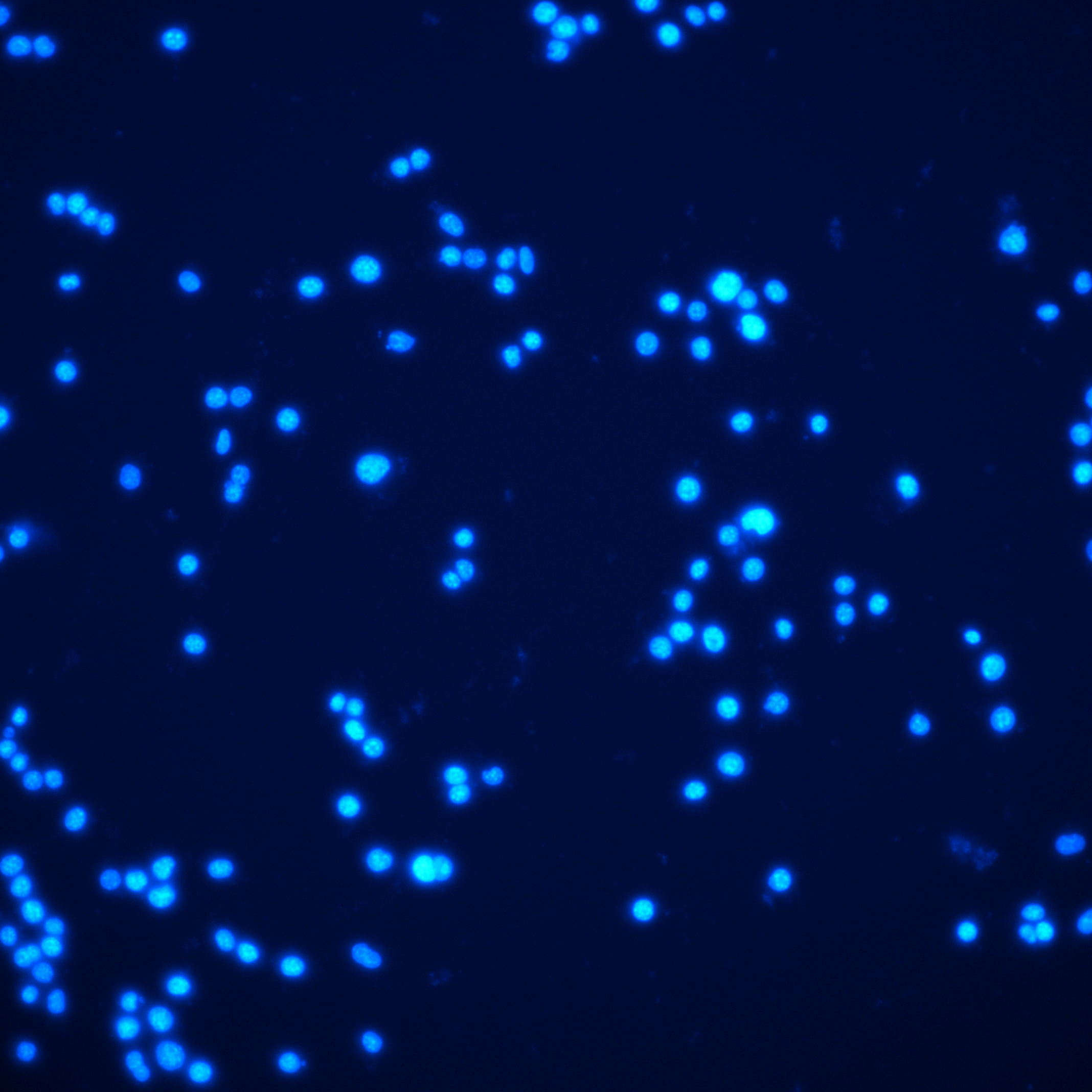

Supplement: Supplementary file 4 [file Data_Sheet_4.ZIP › Immunochemical staining of TLR4-DAPIs/6-LV-TREM2-Oxygen+TAK242-DAPI.tif]

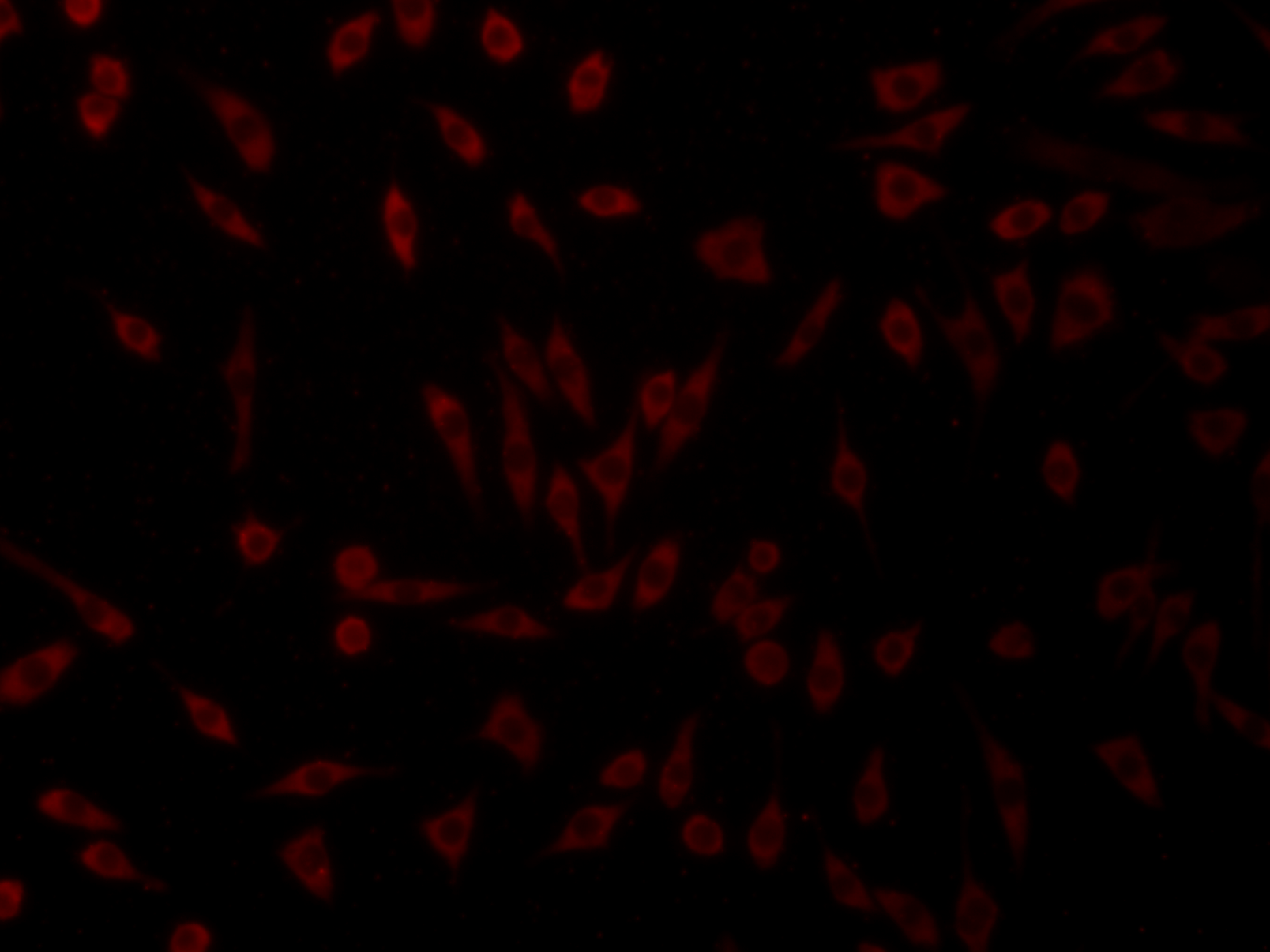

Supplement: Supplementary file 5 [file Data_Sheet_5.ZIP › Immunochemical staining of TREM2/1-BV2-NC.tif]

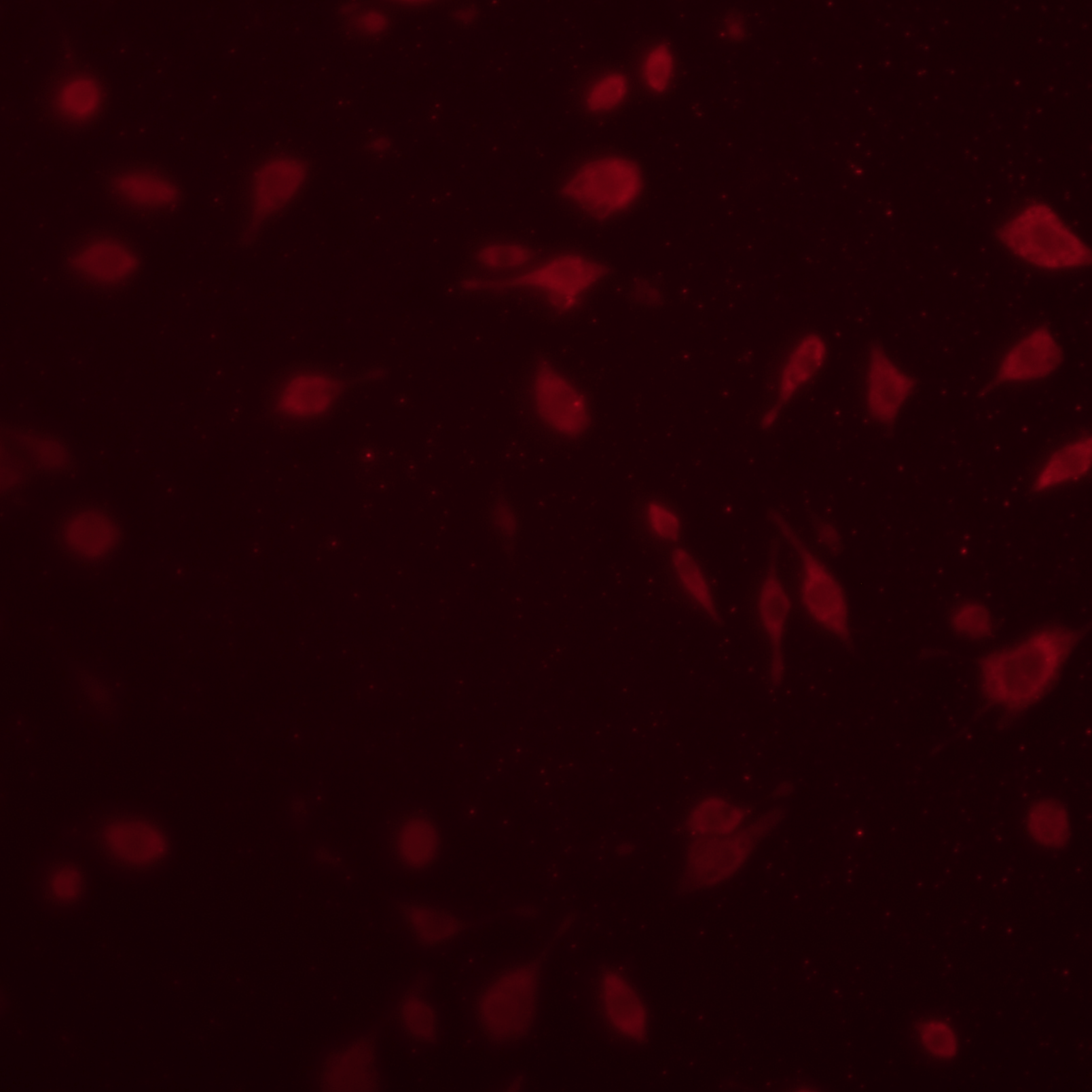

Supplement: Supplementary file 5 [file Data_Sheet_5.ZIP › Immunochemical staining of TREM2/2-BV2-Oxygen.tif]

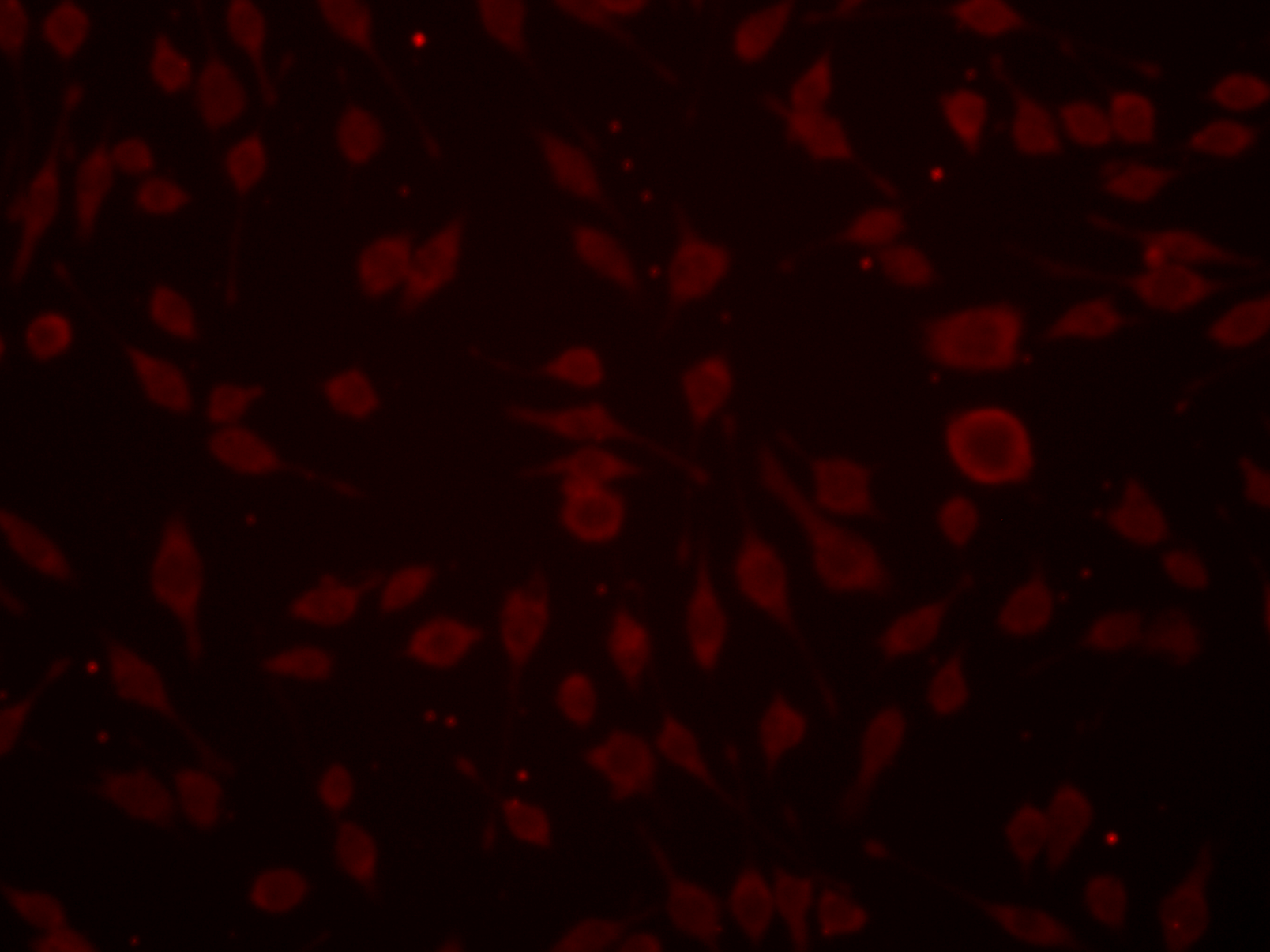

Supplement: Supplementary file 5 [file Data_Sheet_5.ZIP › Immunochemical staining of TREM2/3-BV2-Oxygen+TAK242.tif]

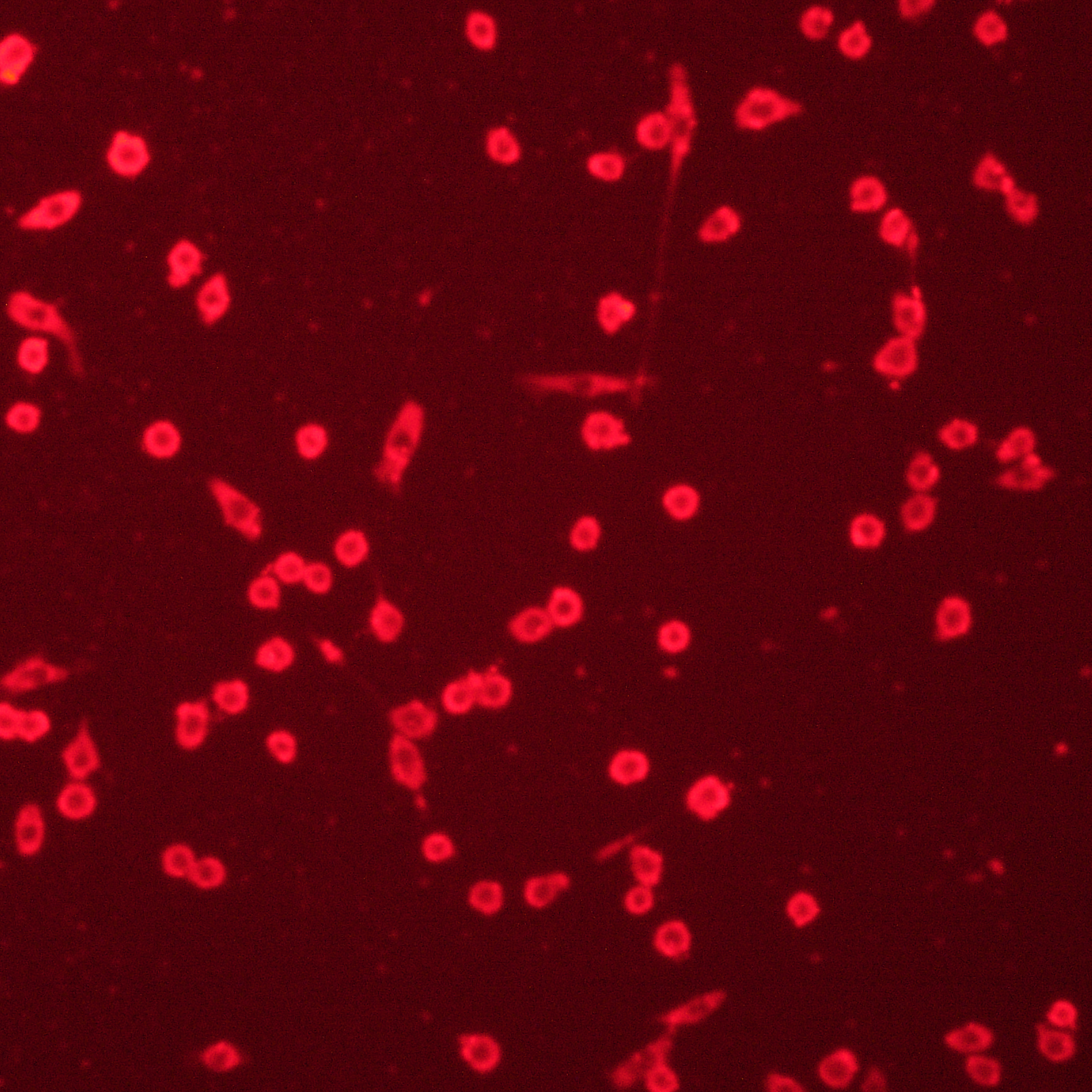

Supplement: Supplementary file 5 [file Data_Sheet_5.ZIP › Immunochemical staining of TREM2/4-LV-TREM2-NC.tif]

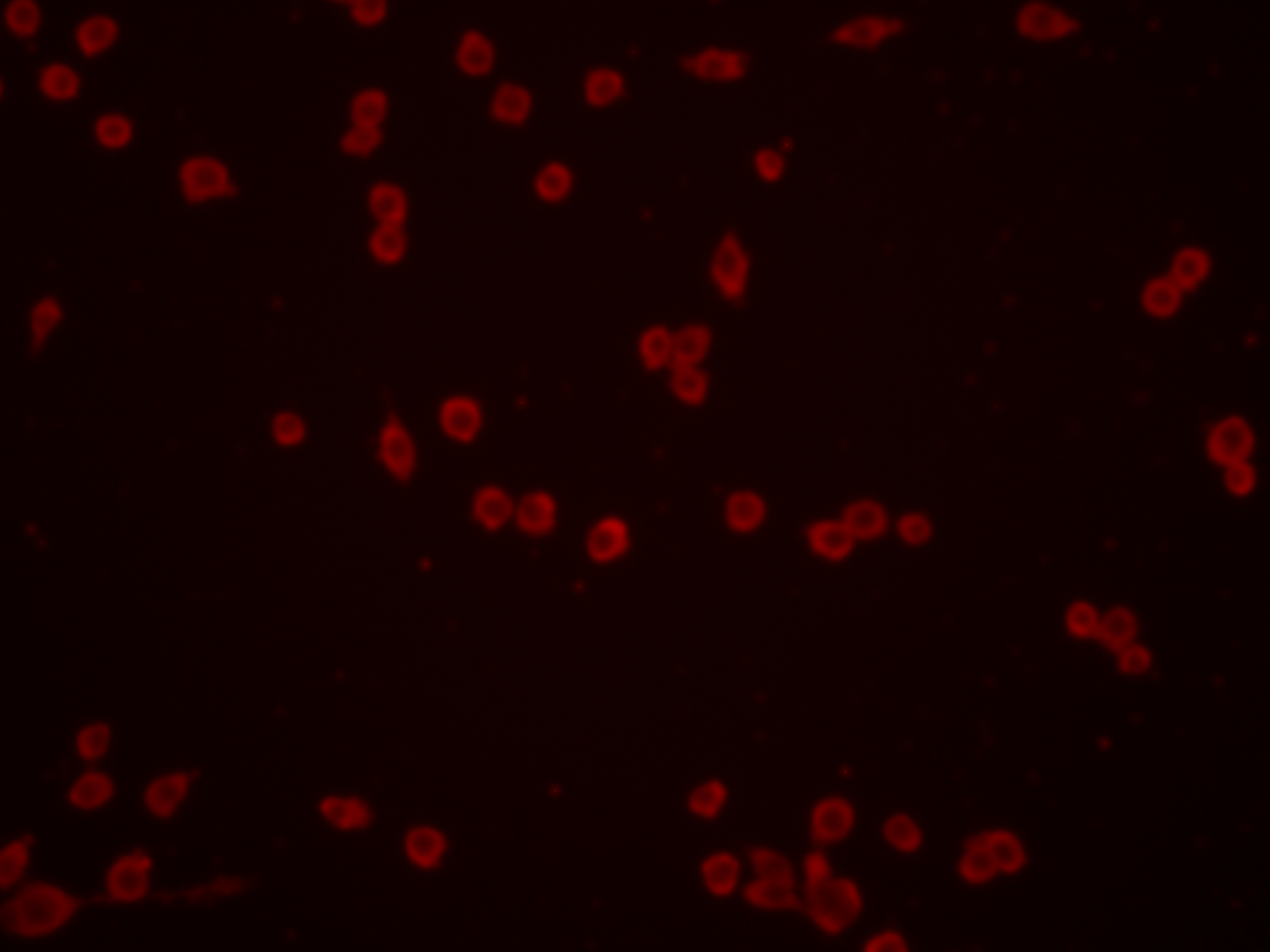

Supplement: Supplementary file 5 [file Data_Sheet_5.ZIP › Immunochemical staining of TREM2/5-LV-TREM2-Oxygen.tif]

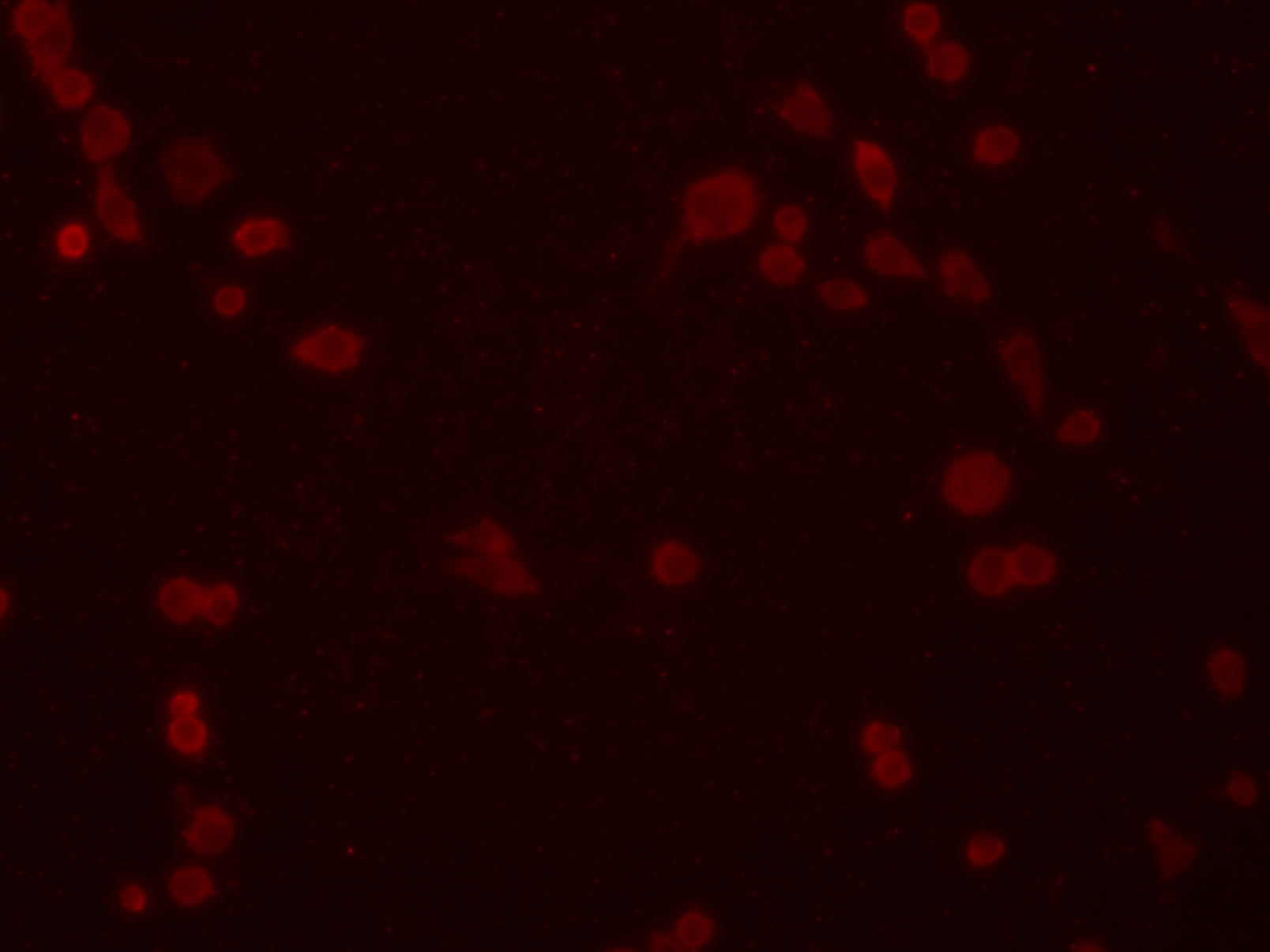

Supplement: Supplementary file 5 [file Data_Sheet_5.ZIP › Immunochemical staining of TREM2/6-LV-TREM2-Oxygen+TAK242.tif]

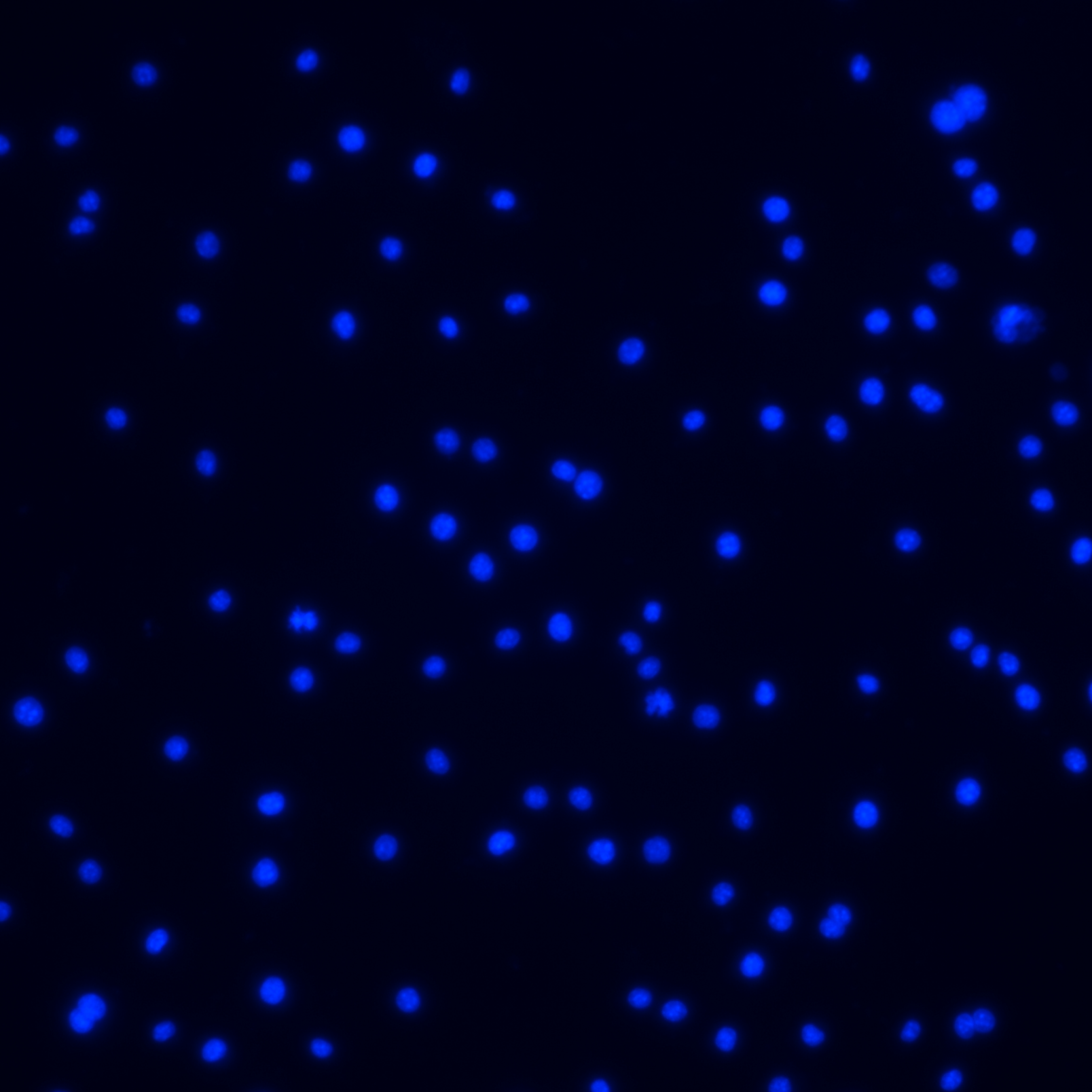

Supplement: Supplementary file 6 [file Data_Sheet_6.ZIP › Immunochemical staining of TREM2-DAPIs/1-BV2-NC-DAPI.tif]

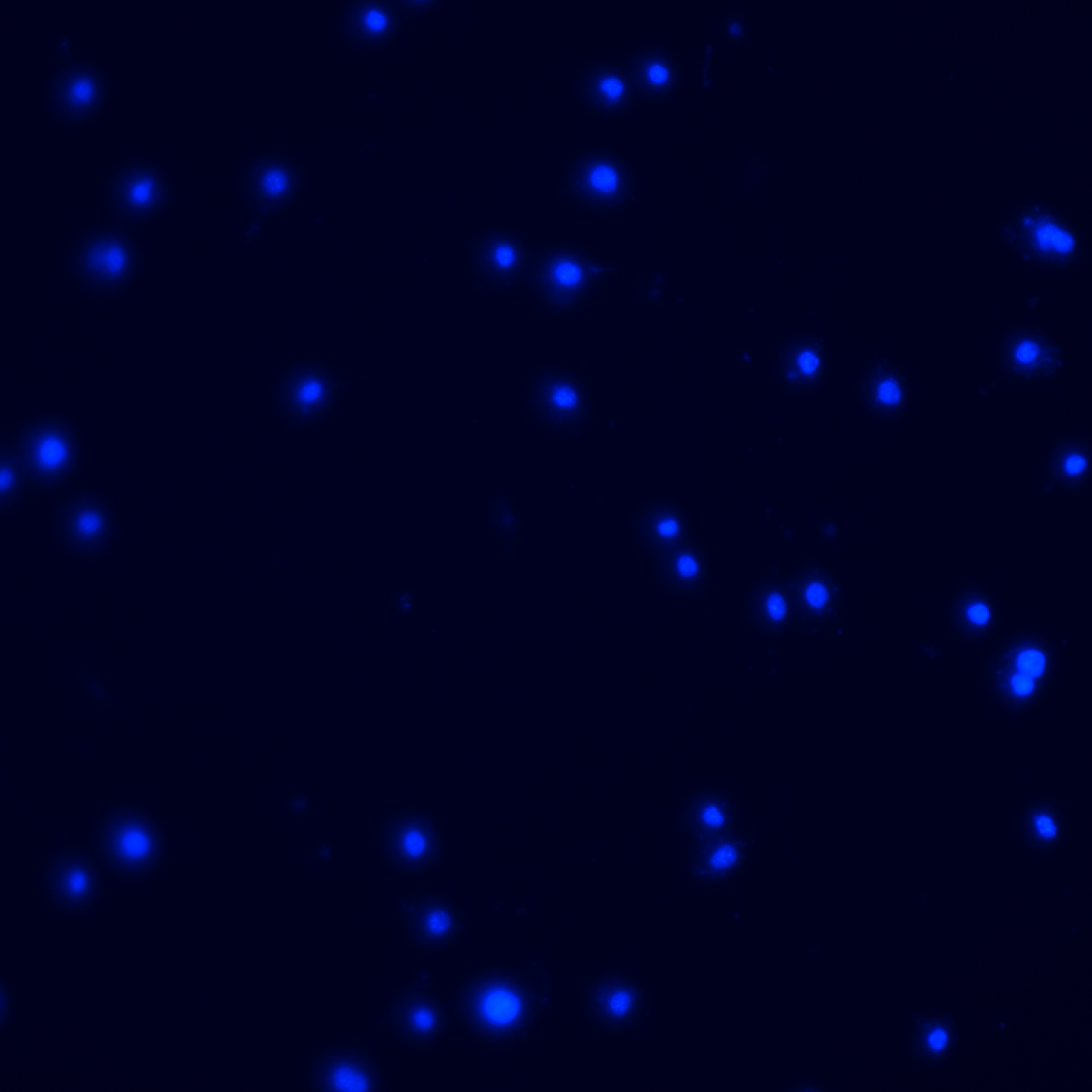

Supplement: Supplementary file 6 [file Data_Sheet_6.ZIP › Immunochemical staining of TREM2-DAPIs/2-BV2-Oxygen-DAPI.tif]

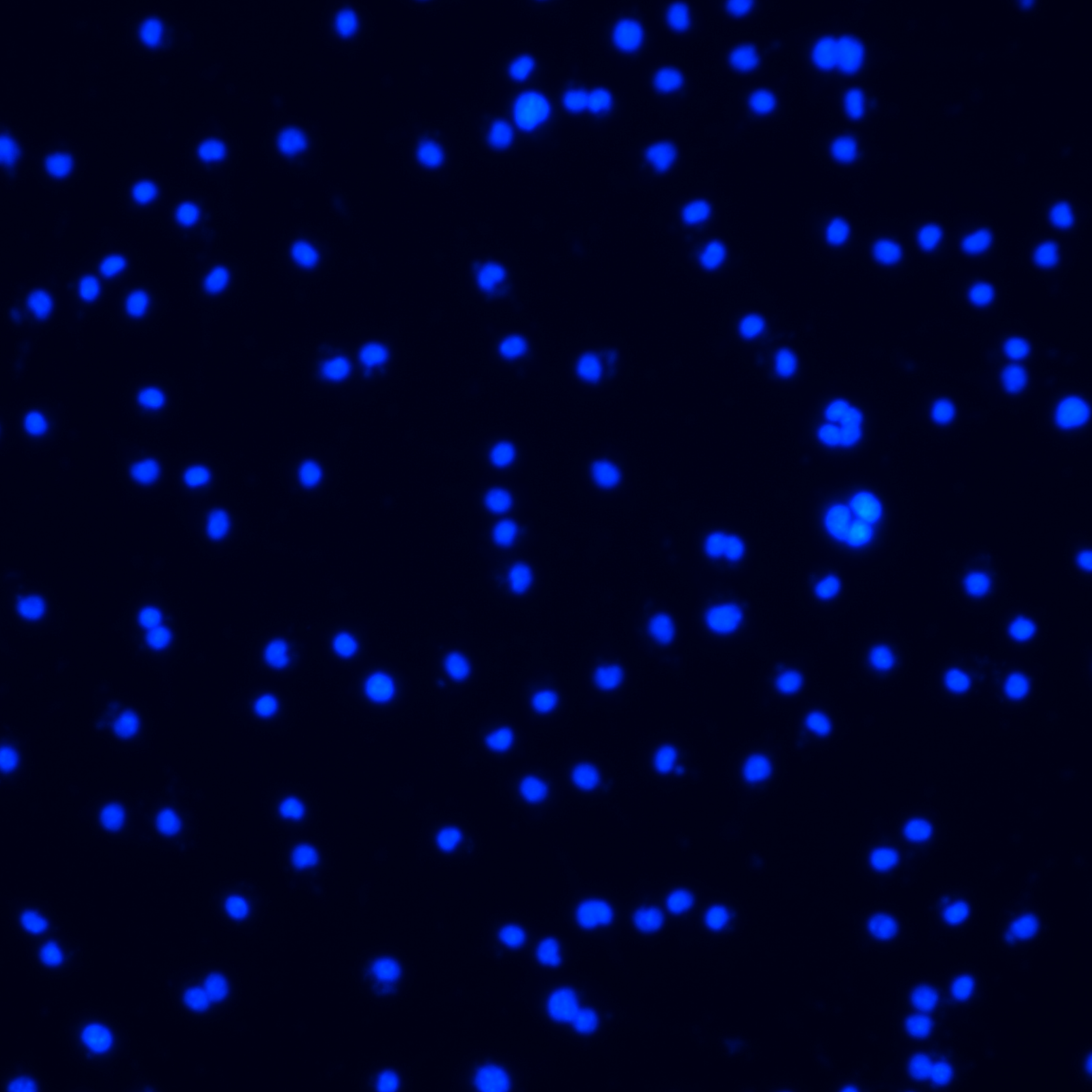

Supplement: Supplementary file 6 [file Data_Sheet_6.ZIP › Immunochemical staining of TREM2-DAPIs/3-BV2-Oxygen+TAK242-DAPI.tif]

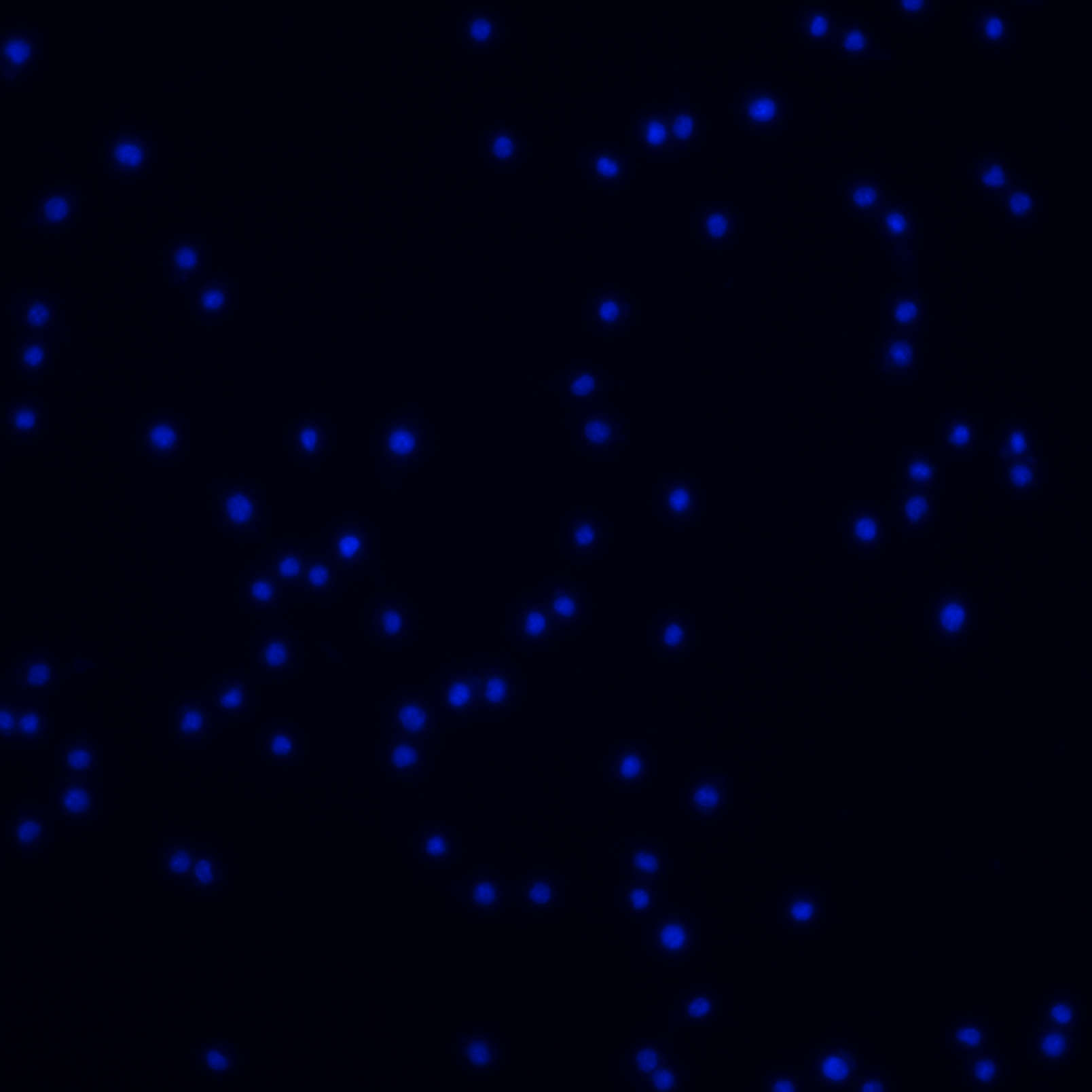

Supplement: Supplementary file 6 [file Data_Sheet_6.ZIP › Immunochemical staining of TREM2-DAPIs/4-LV-TREM2-NC-DAPI.tif]

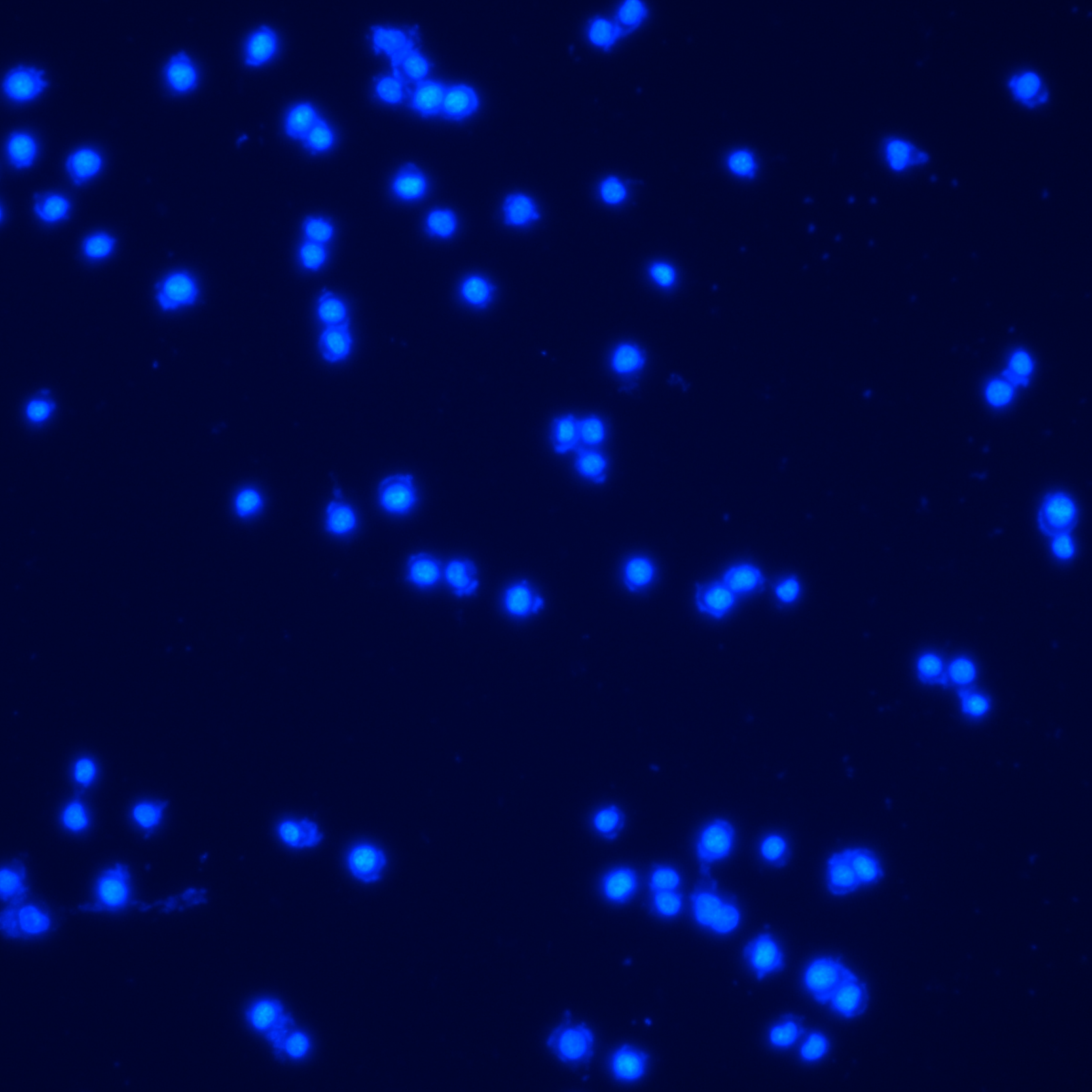

Supplement: Supplementary file 6 [file Data_Sheet_6.ZIP › Immunochemical staining of TREM2-DAPIs/5-LV-TREM2-Oxygen-DAPI.tif]

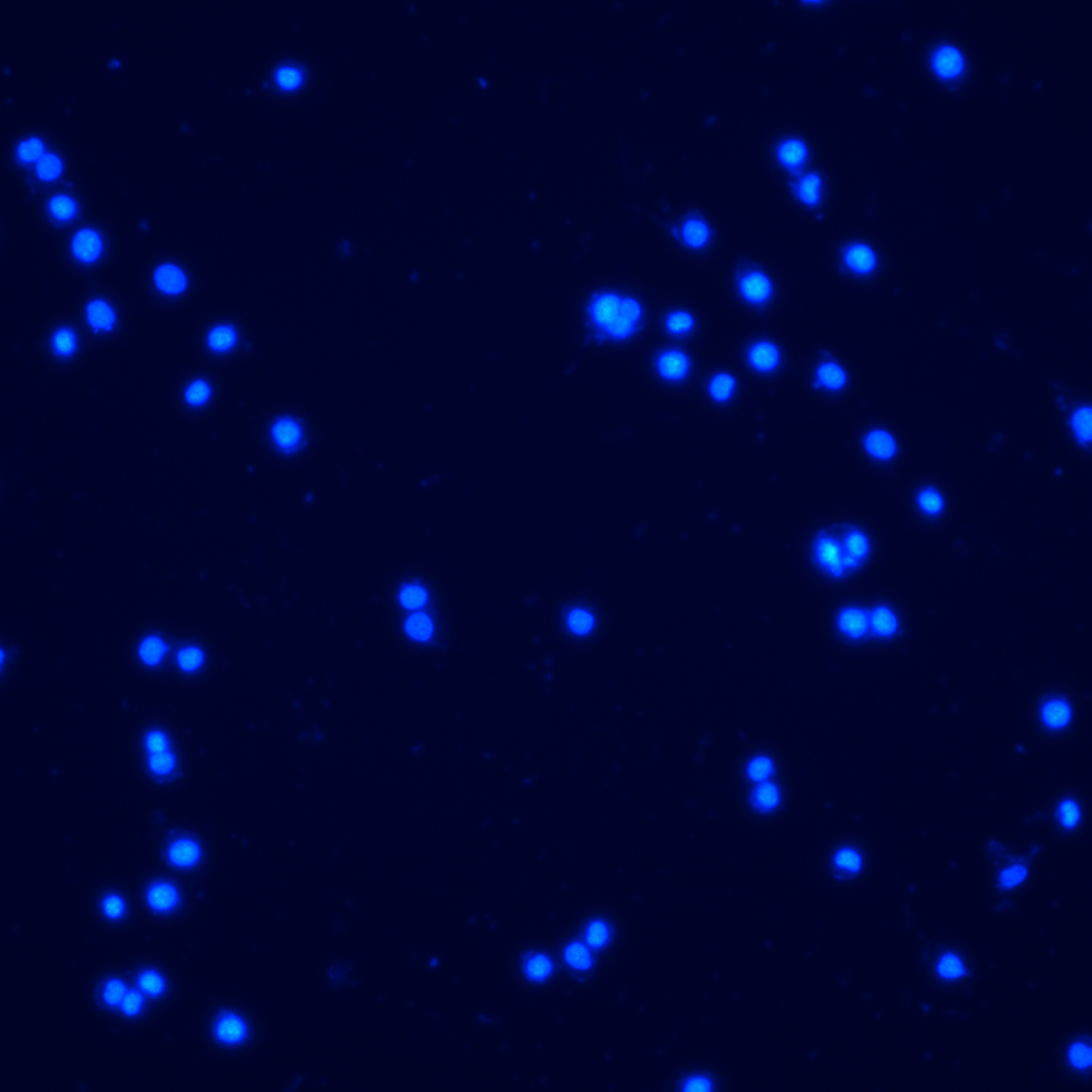

Supplement: Supplementary file 6 [file Data_Sheet_6.ZIP › Immunochemical staining of TREM2-DAPIs/6-LV-TREM2-Oxygen+TAK242-DAPI.tif]

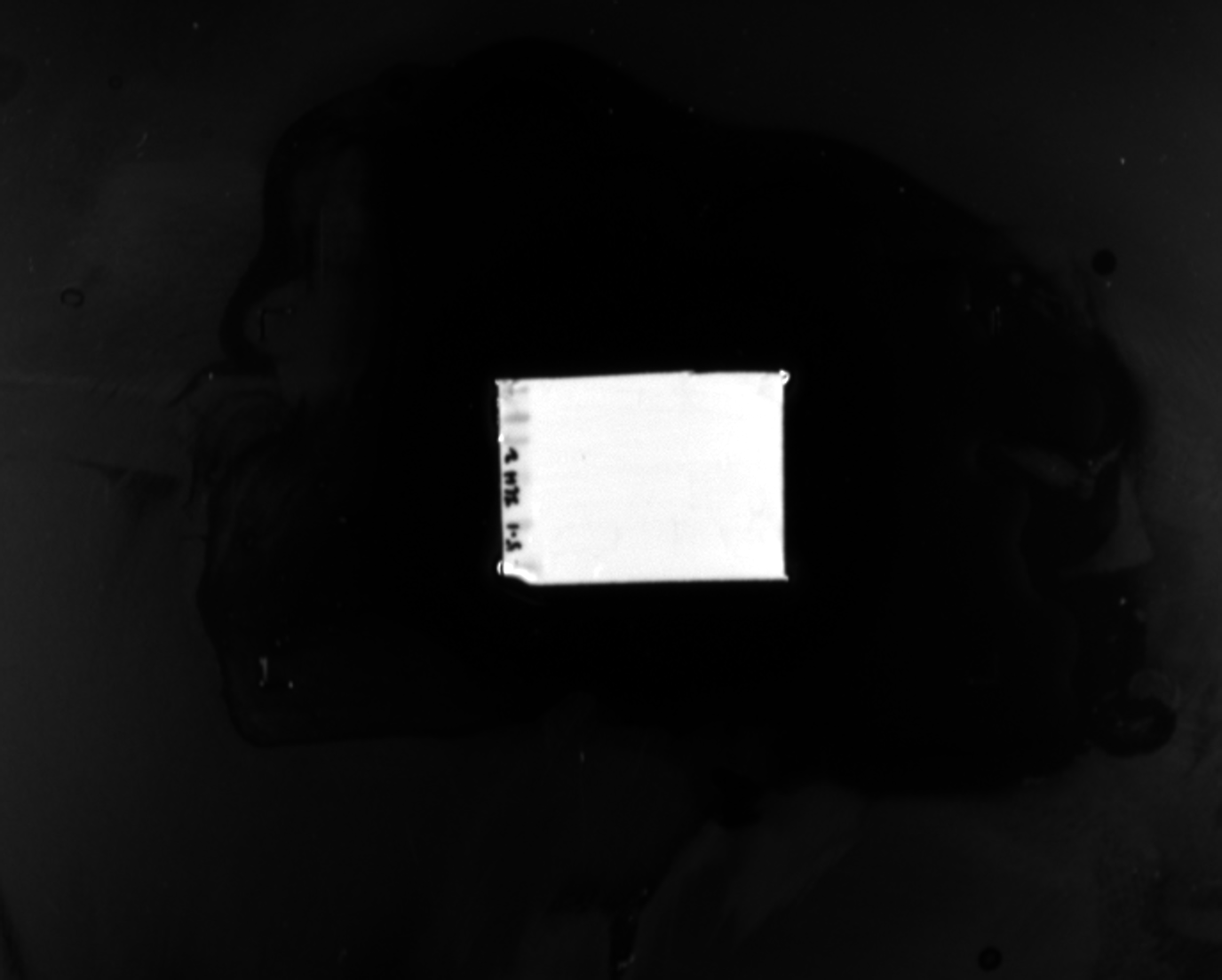

Supplement: Supplementary file 7 [file Data_Sheet_7.ZIP › Original files of supplementary figure 1/20220502marker of tubulin and TNF-a┴.Tif]

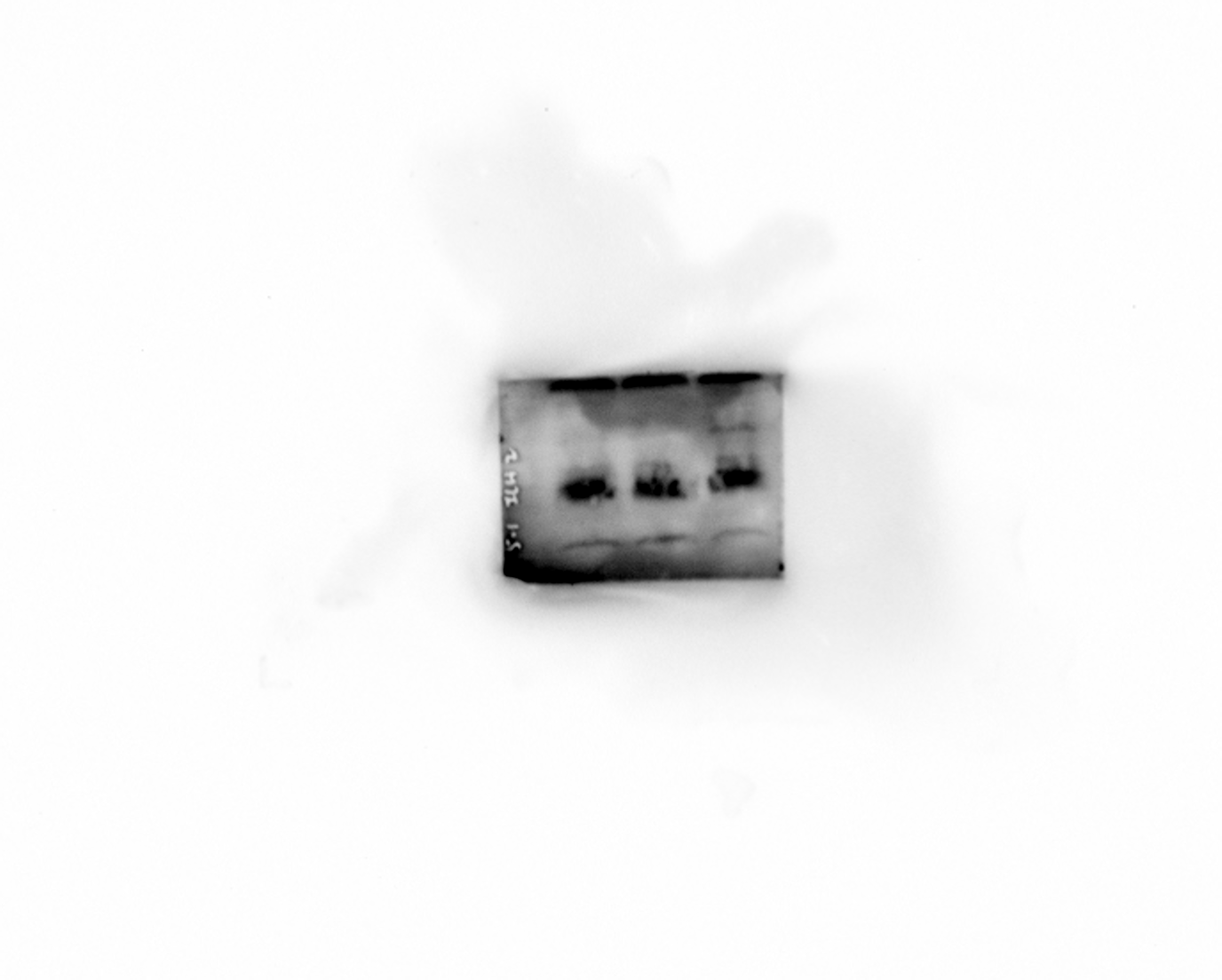

Supplement: Supplementary file 7 [file Data_Sheet_7.ZIP › Original files of supplementary figure 1/20220502tubulin and TNF-a┴ (2).Tif]

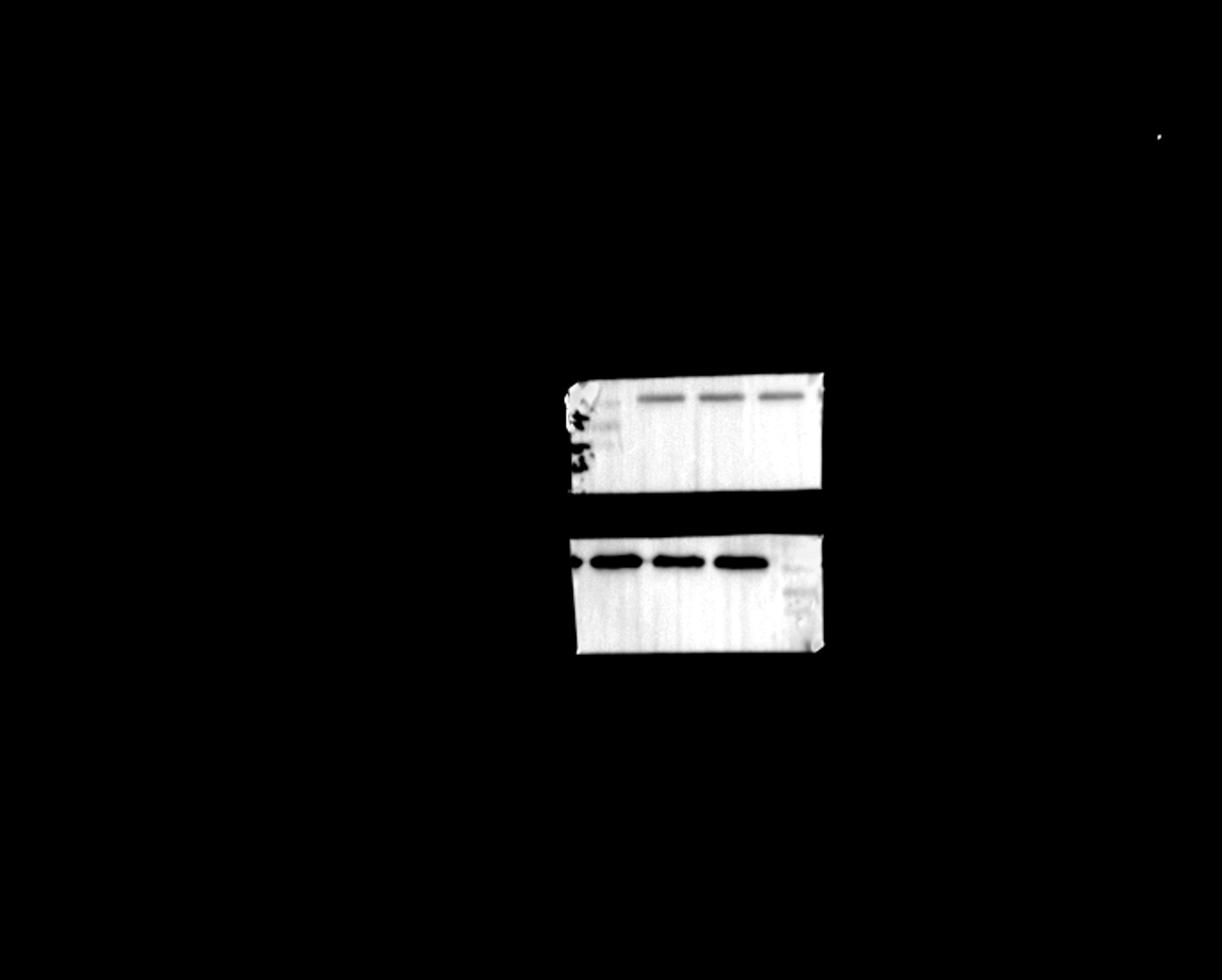

Supplement: Supplementary file 7 [file Data_Sheet_7.ZIP › Original files of supplementary figure 1/TLR4 and IL-1a┬.Tif]

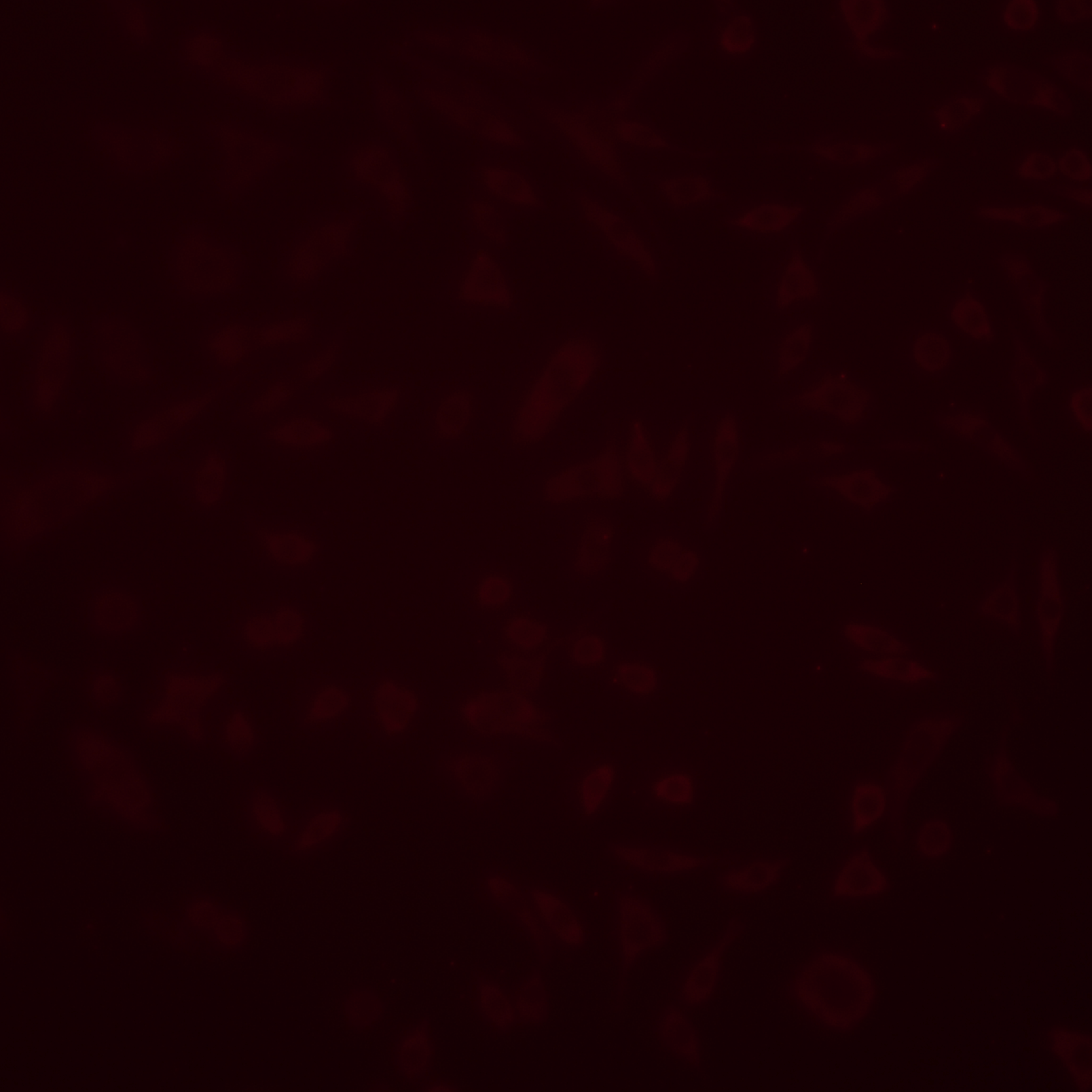

Supplement: Supplementary file 8 [file Data_Sheet_8.ZIP › original images of TREM2 Immunofluorescence staining/BV2-control-2.tif]

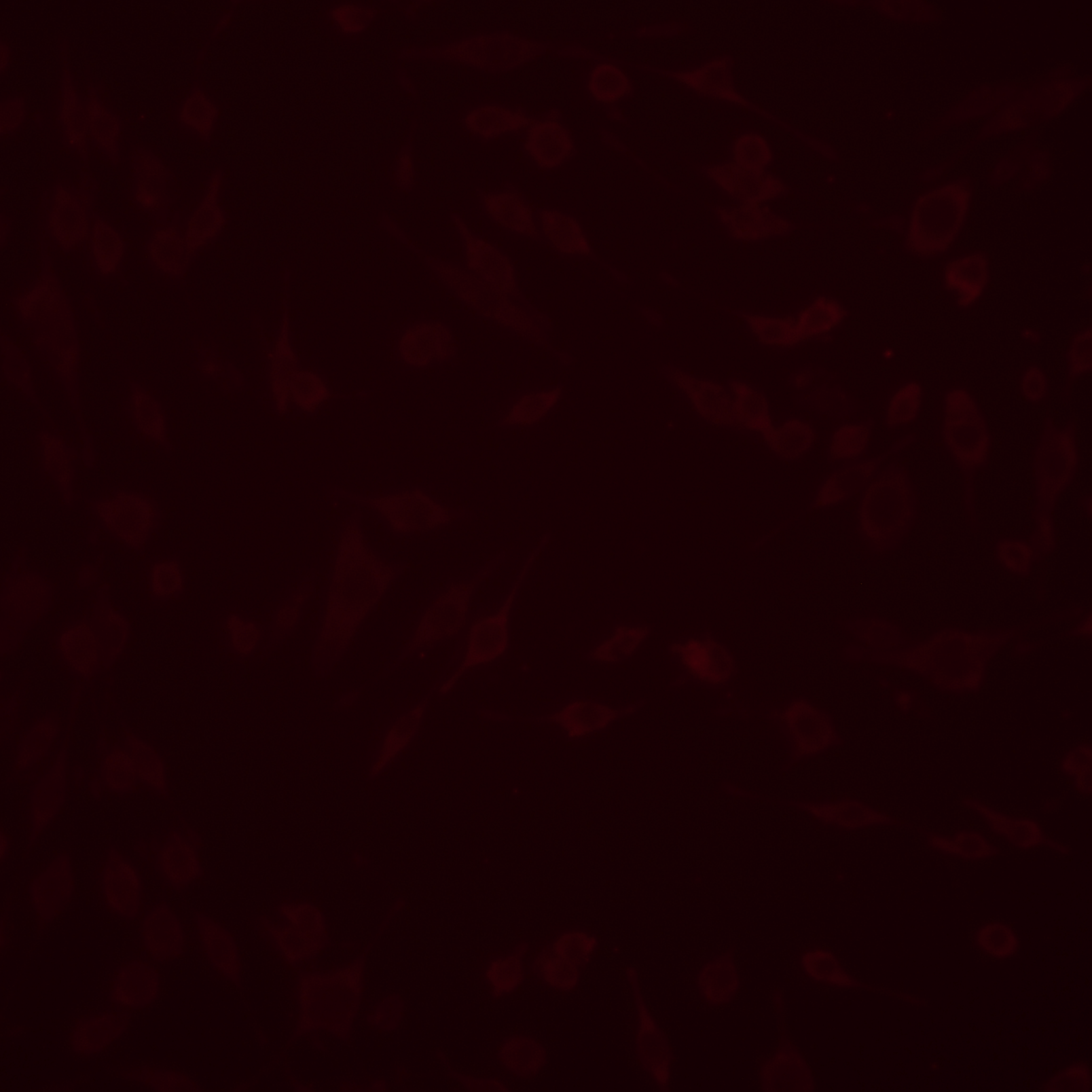

Supplement: Supplementary file 8 [file Data_Sheet_8.ZIP › original images of TREM2 Immunofluorescence staining/BV2-control-3.tif]

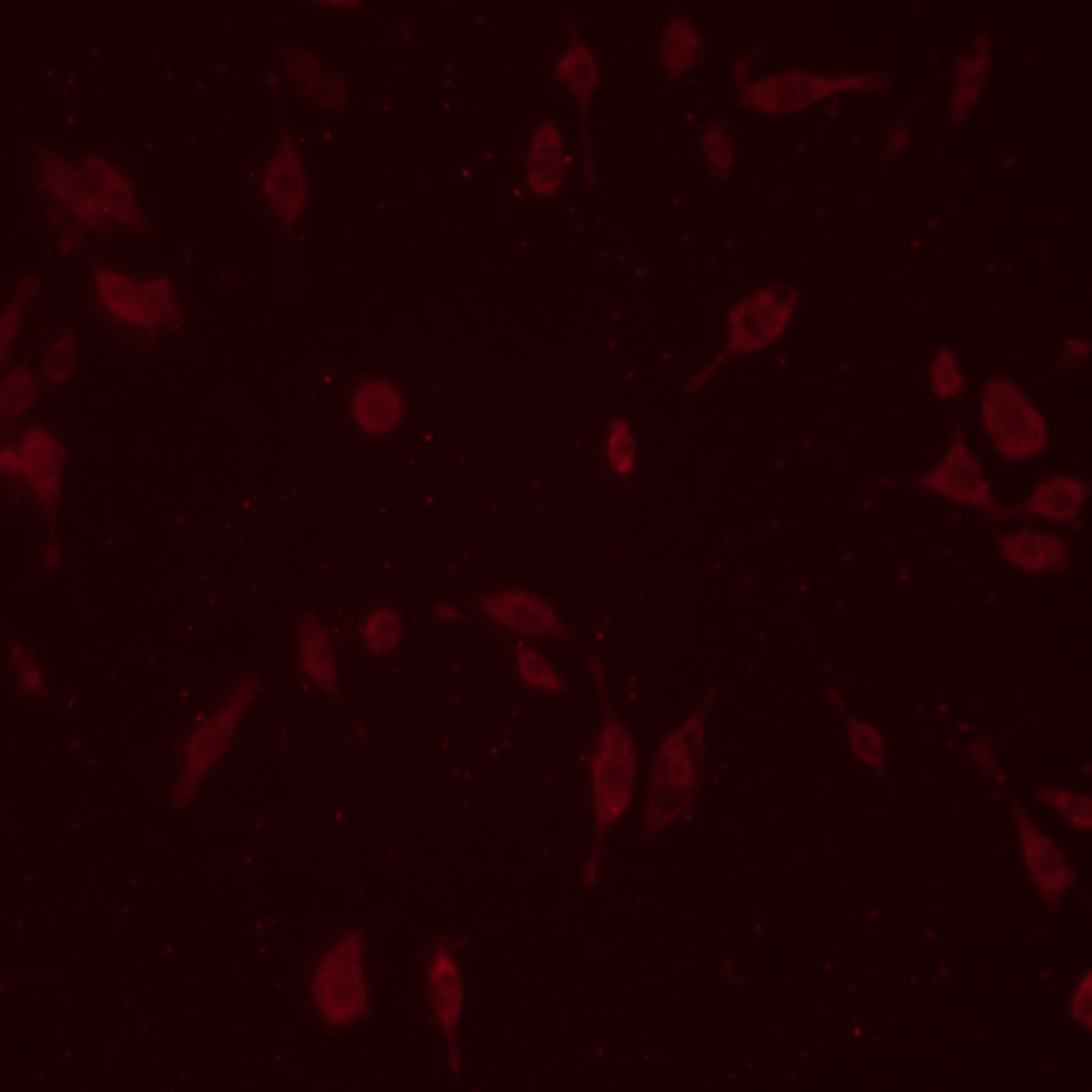

Supplement: Supplementary file 8 [file Data_Sheet_8.ZIP › original images of TREM2 Immunofluorescence staining/BV2-oxyhb-2.tif]

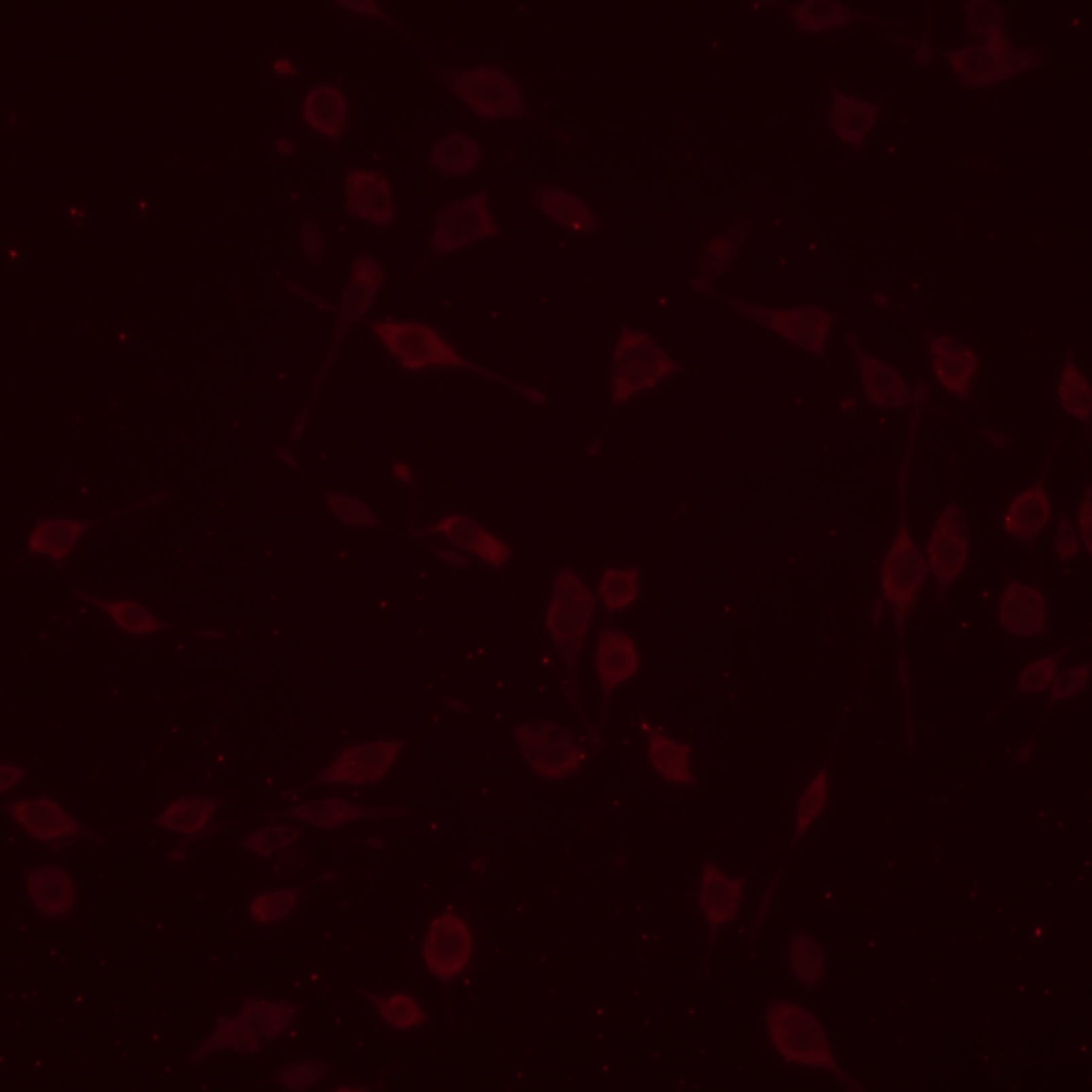

Supplement: Supplementary file 8 [file Data_Sheet_8.ZIP › original images of TREM2 Immunofluorescence staining/BV2-oxyhb-3.tif]

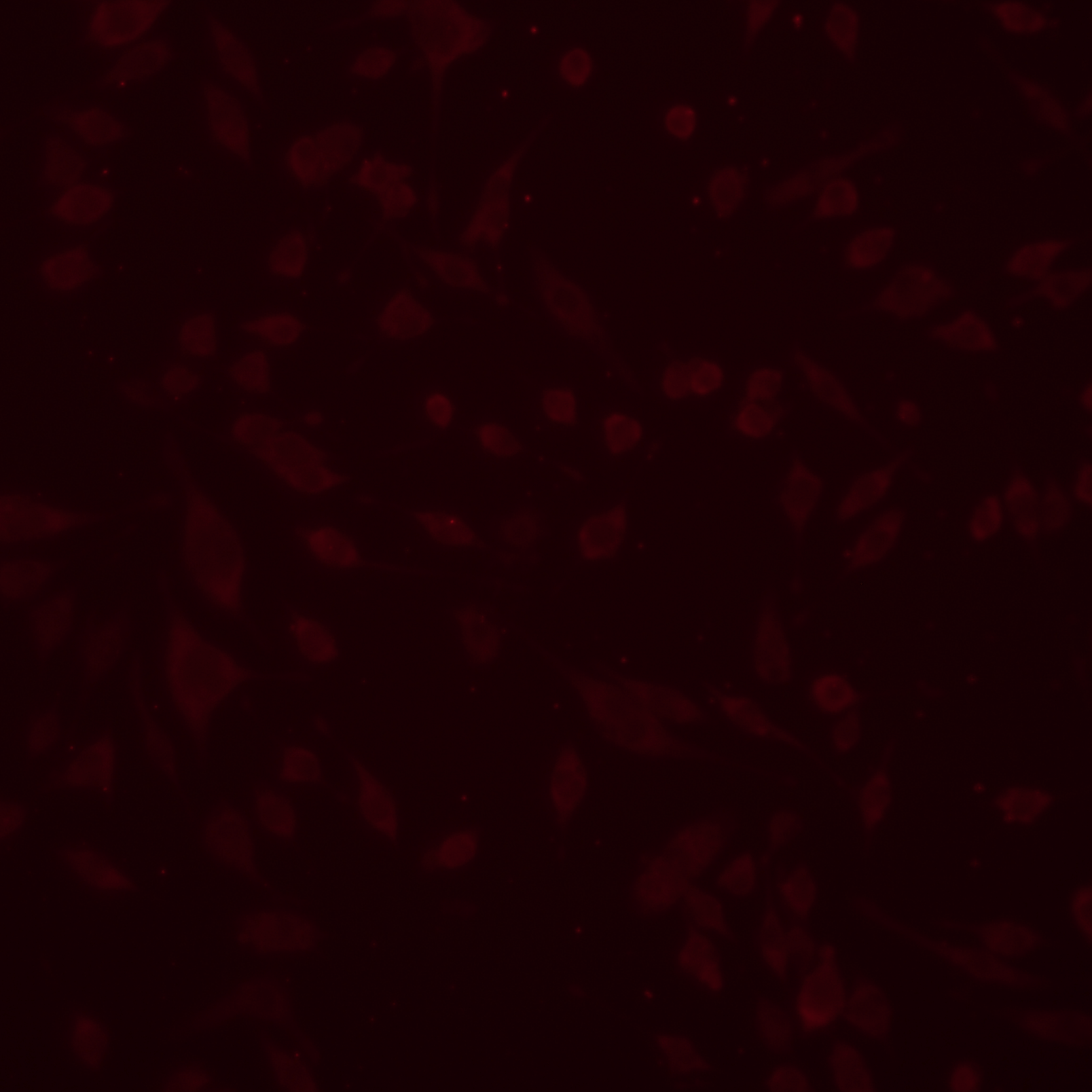

Supplement: Supplementary file 8 [file Data_Sheet_8.ZIP › original images of TREM2 Immunofluorescence staining/BV2-TAK242-2.tif]

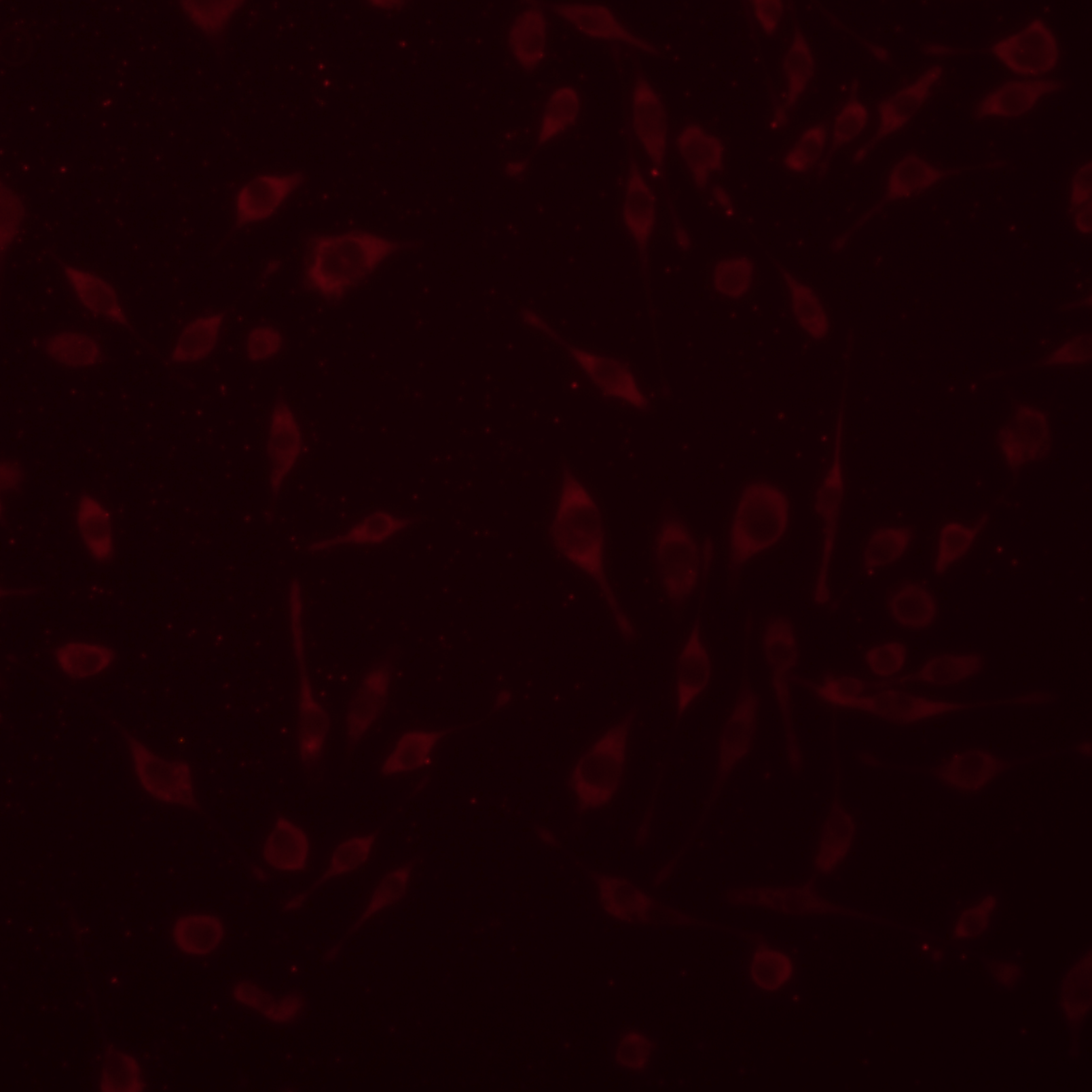

Supplement: Supplementary file 8 [file Data_Sheet_8.ZIP › original images of TREM2 Immunofluorescence staining/BV2-TAK242-3.tif]

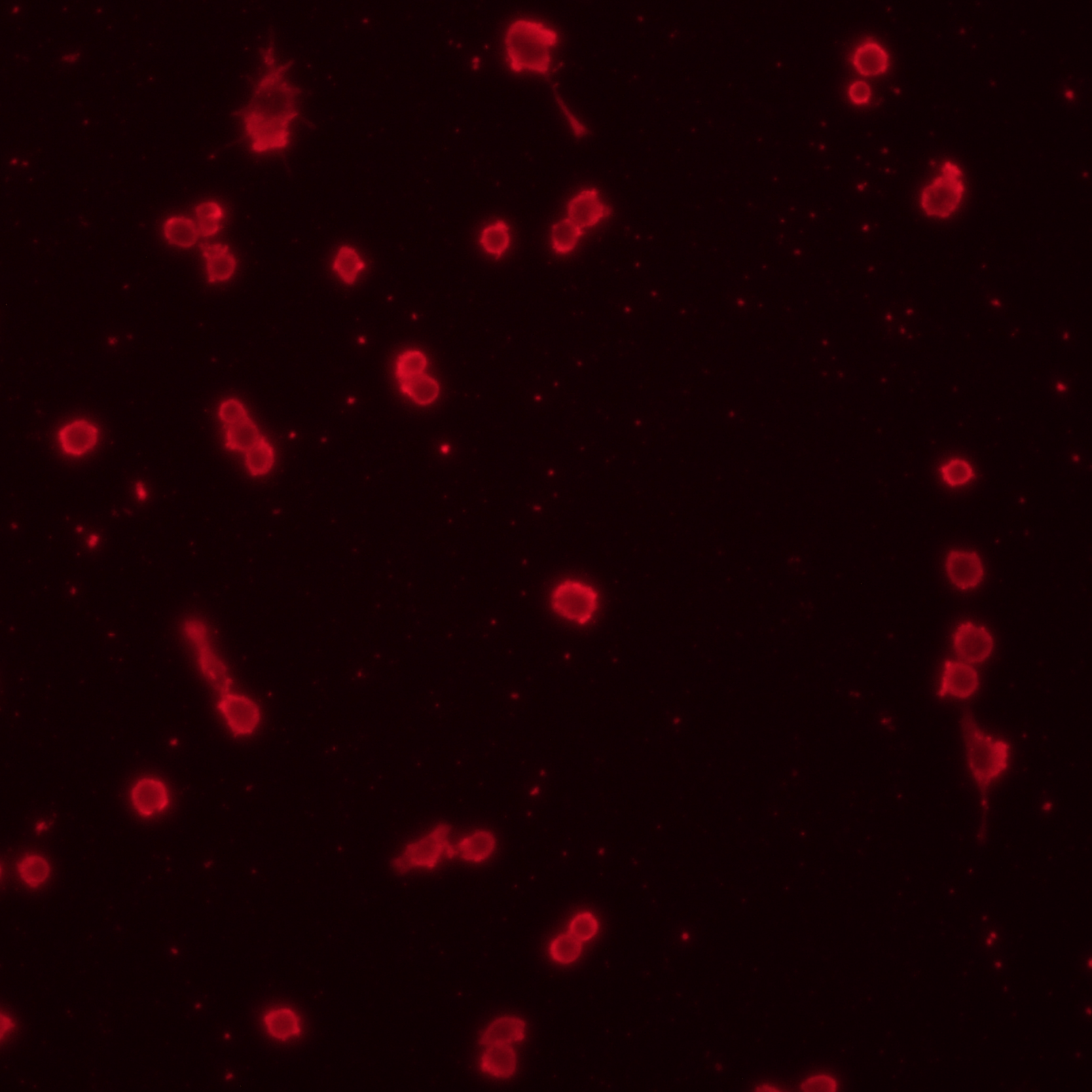

Supplement: Supplementary file 8 [file Data_Sheet_8.ZIP › original images of TREM2 Immunofluorescence staining/LV-TREM2-control-2.tif]

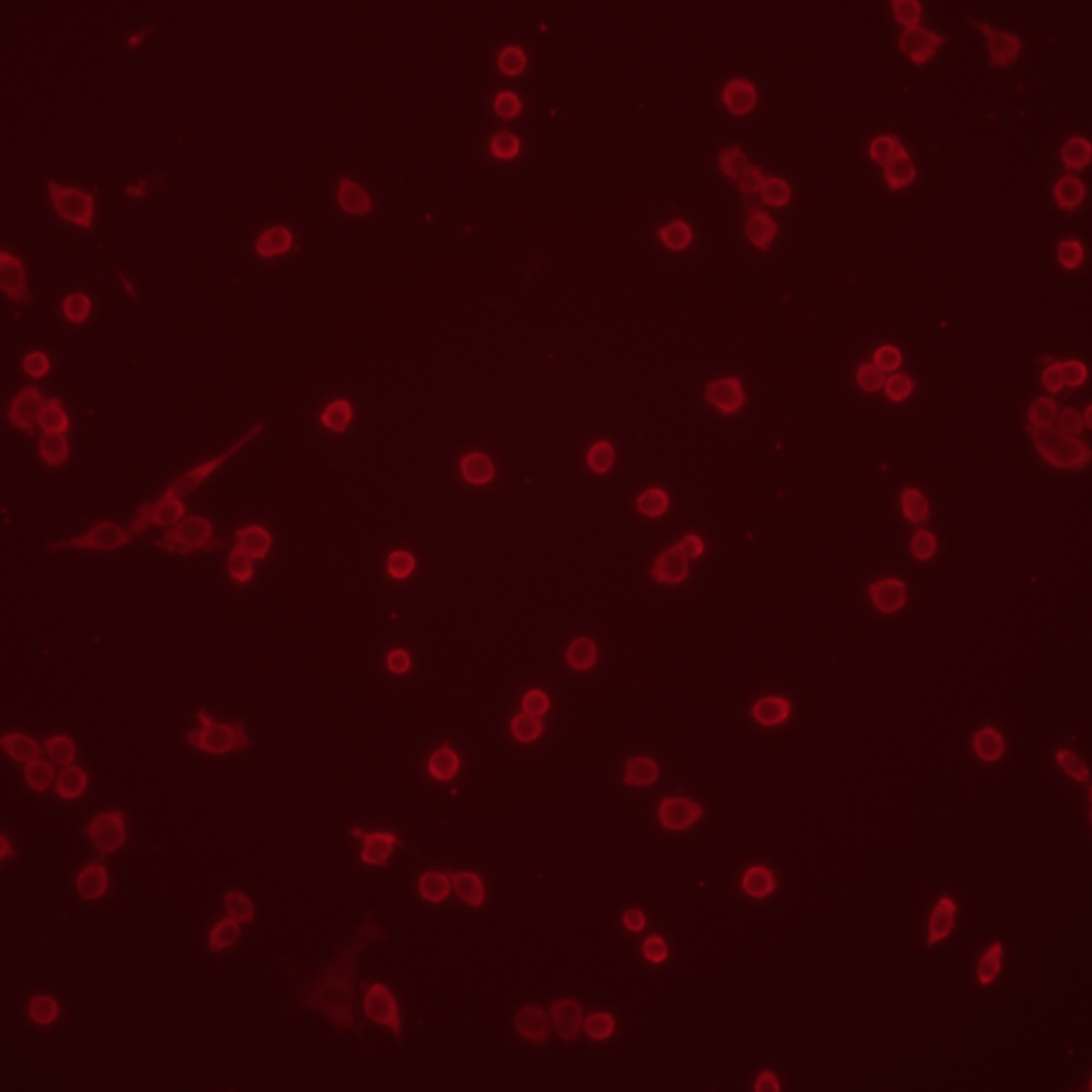

Supplement: Supplementary file 8 [file Data_Sheet_8.ZIP › original images of TREM2 Immunofluorescence staining/LV-TREM2-control-3.tif]

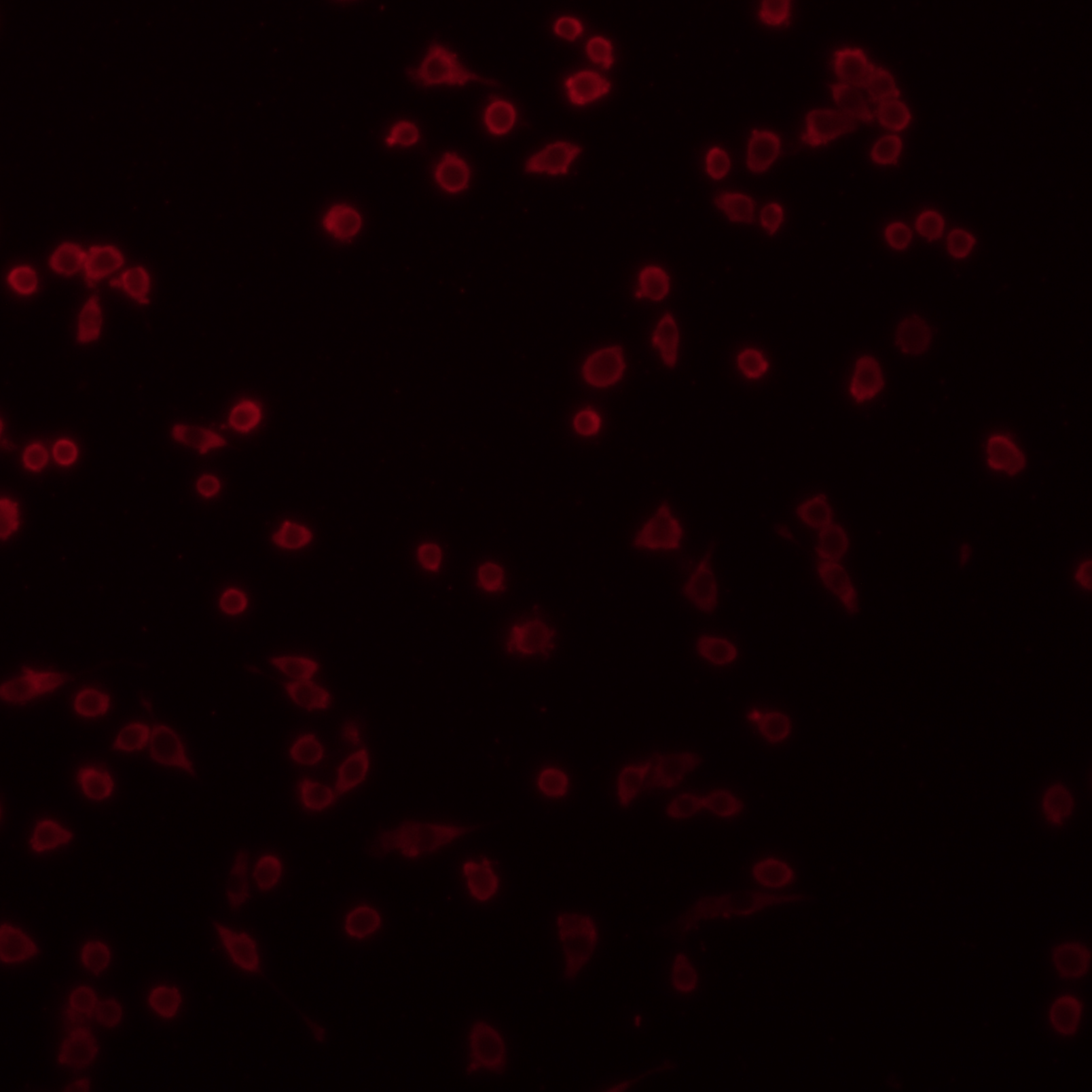

Supplement: Supplementary file 8 [file Data_Sheet_8.ZIP › original images of TREM2 Immunofluorescence staining/LV-TREM2-oxyhb-2.tif]

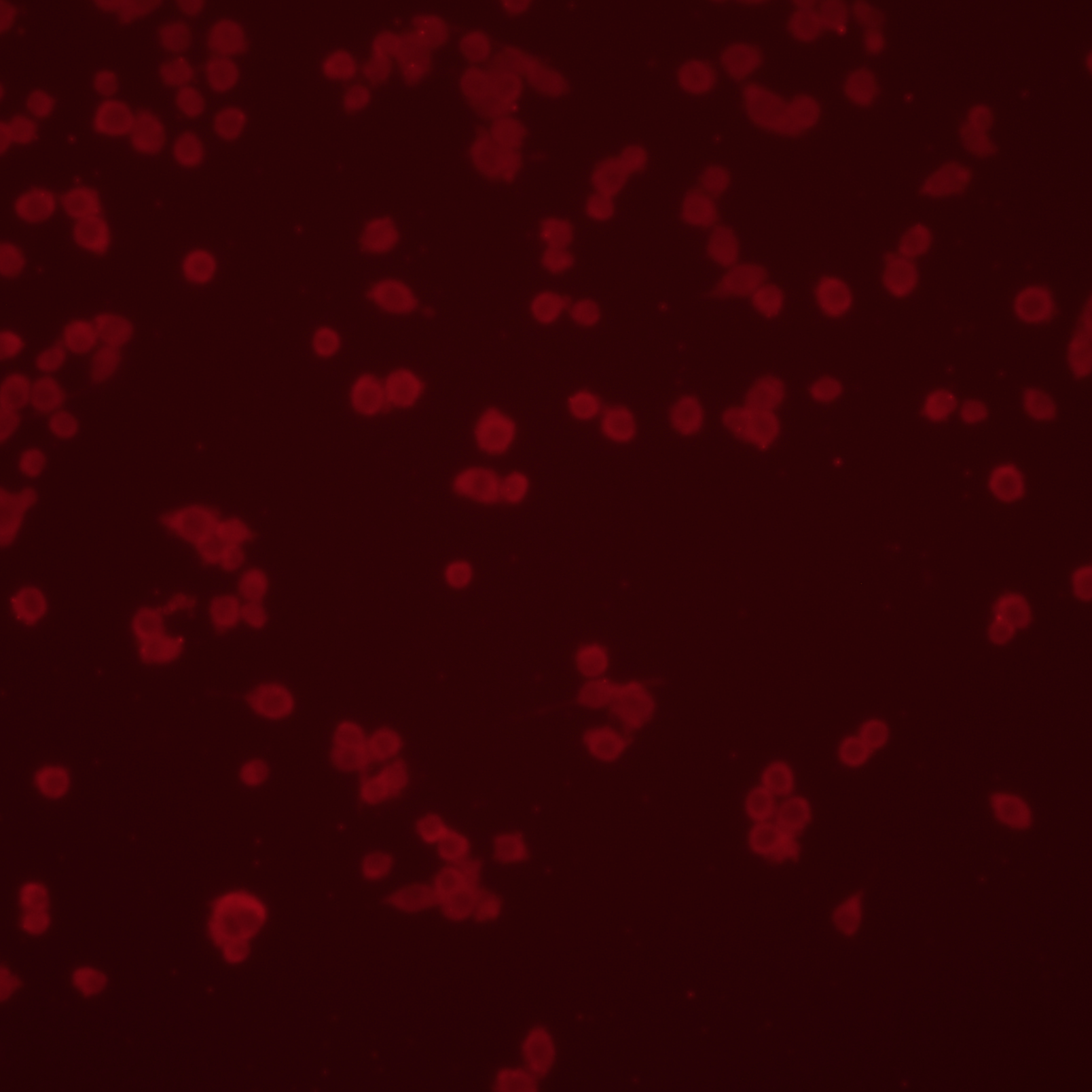

Supplement: Supplementary file 8 [file Data_Sheet_8.ZIP › original images of TREM2 Immunofluorescence staining/LV-TREM2-oxyhb-3.tif]

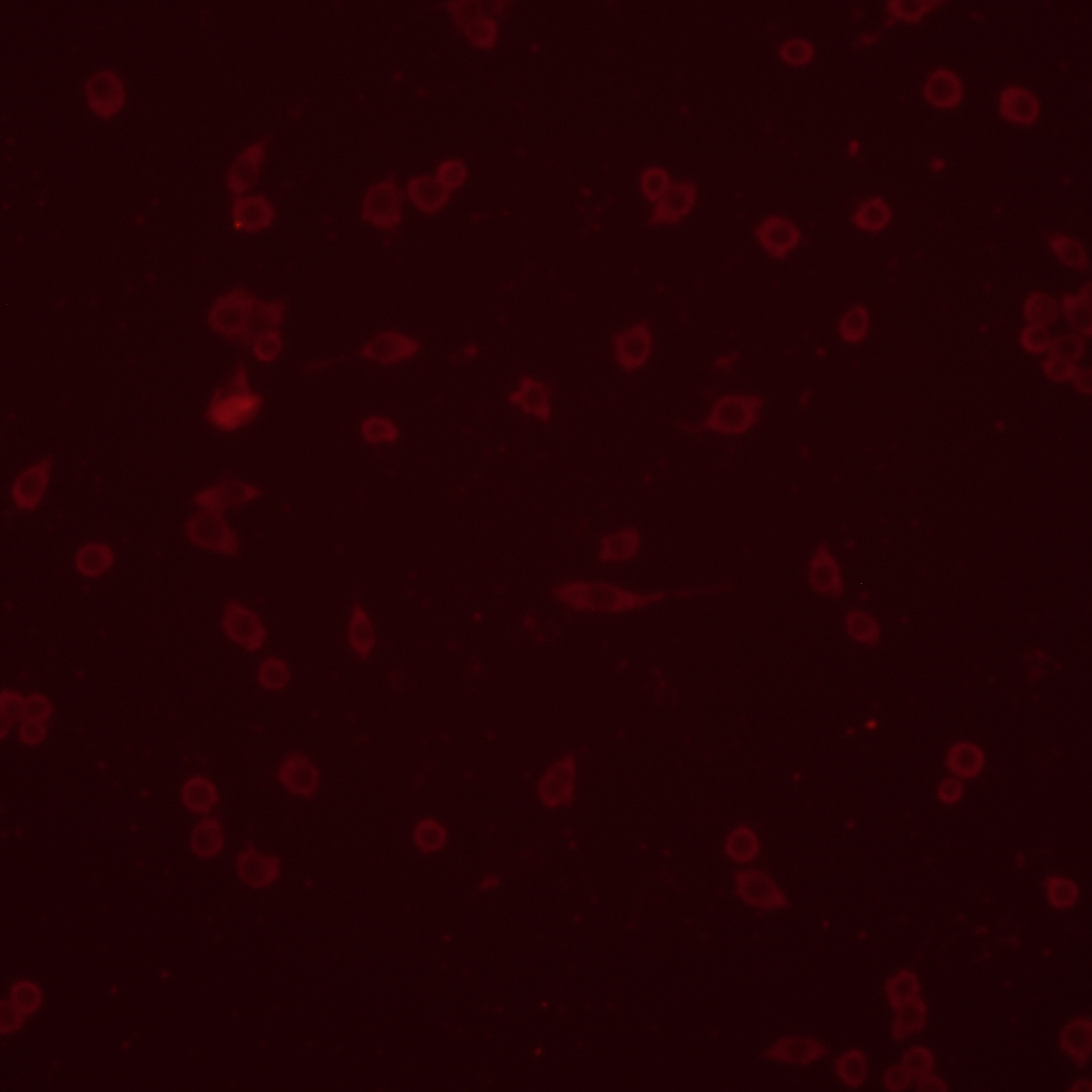

Supplement: Supplementary file 8 [file Data_Sheet_8.ZIP › original images of TREM2 Immunofluorescence staining/LV-TREM2-TAK242-2.tif]

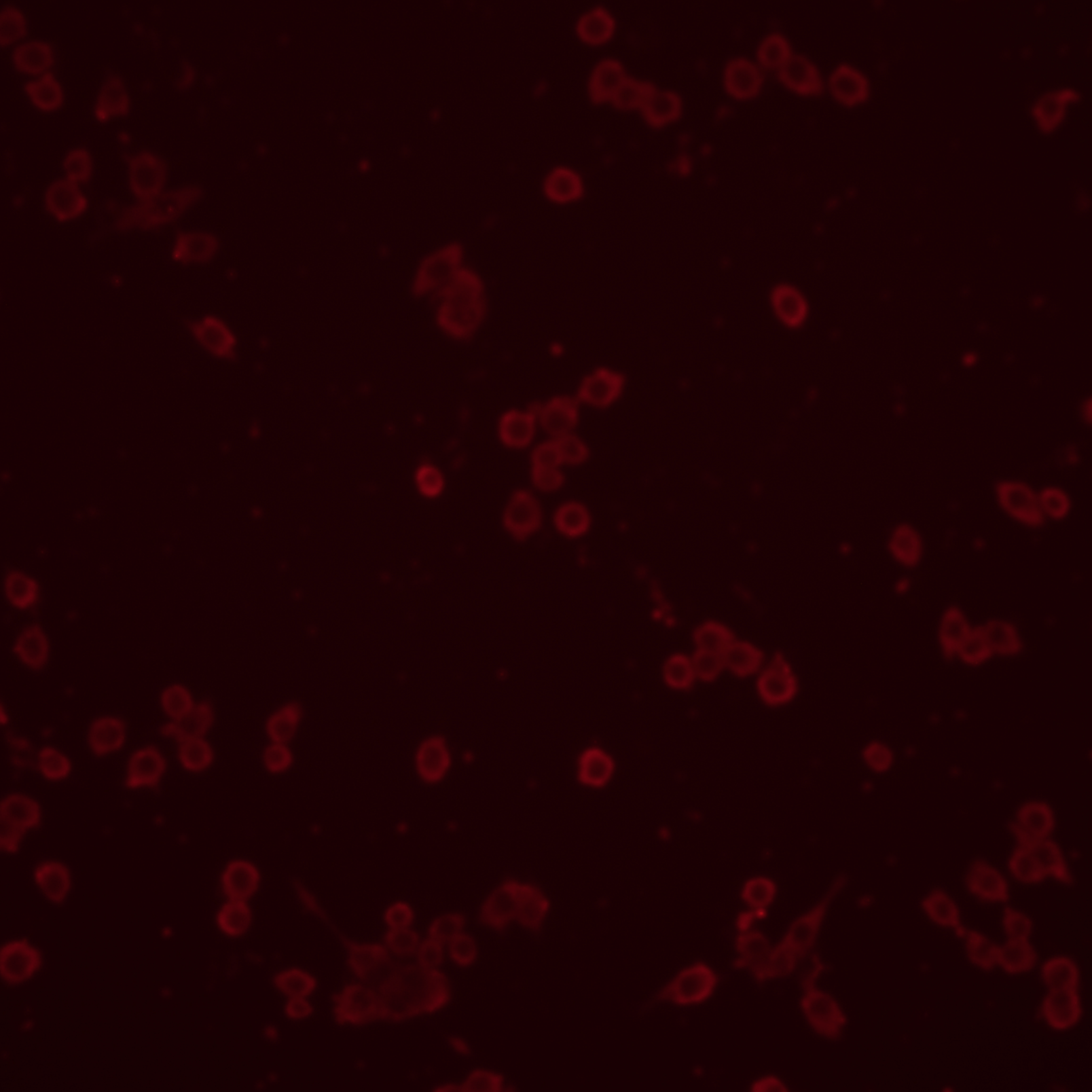

Supplement: Supplementary file 8 [file Data_Sheet_8.ZIP › original images of TREM2 Immunofluorescence staining/LV-TREM2-TAK242-3.tif]
